# ADAR2 induces the differentiation of osteosarcoma cells by editing activity on IGFBP7: new implications for therapy

**DOI:** 10.1038/s41413-026-00516-6

**Published:** 2026-04-03

**Authors:** Michela Rossi, Federica Scotto di Carlo, Jacopo Di Gregorio, Sharon Russo, Laura Di Giuseppe, Giulia Battafarano, Sara Terreri, Olivia Pagliarosi, Domenico Alessandro Silvestris, Marco Corona, Adriano Barra, Marco Pezzullo, Cristiano De Stefanis, Simone Pelle, Pier Francesco Costici, Salvatore Minisola, Jessica Pepe, Franco Locatelli, Fernando Gianfrancesco, Angela Gallo, Andrea Del Fattore

**Affiliations:** 1 Bone Physiopathology Research Unit, Translational Pediatric and Clinical Genetic Research Division, Bambino Gesù Children’s Hospital, IRCCS, Rome, Italy; 2 Institute of Genetics and Biophysics “Adriano Buzzati-Traverso”, National Research Council of Italy, Naples, Italy; 3 Department of Biotechnological and Applied Clinical Sciences, University of L’Aquila, L’Aquila, Italy; 4 Department of Medical and Cardiovascular Sciences, Sapienza University, Rome, Italy; 5 Unit of Genetic and Epigenetics of pediatric tumors, Oncohaematology Research Division, Bambino Gesù Children’s Hospital, IRCCS, Rome, Italy; 6 Research Laboratories, Bambino Gesù Children’s Hospital, IRCCS, Rome, Italy; 7 “Polo Sanitario San Feliciano—Villa Aurora” Clinic, Rome, Italy; 8 Department of General Surgery and Medical Surgical Specialties, Orthopedics Unit, Bambino Gesù Children’s Hospital, IRCCS, Rome, Palidoro Italy; 9 Department of Life Sciences and Public Health, Catholic University of the Sacred Heart, Rome, Italy

**Keywords:** Bone cancer, Pathogenesis

## Abstract

Osteosarcoma is a highly malignant bone tumor which primarily affects the juvenile population and is characterized by high rate of recurrence and metastasis. RNA editing has emerged as a key process in cancer progression. Herein, we investigated the role of RNA editing enzyme ADAR2 (Adenosine Deaminase Acting on RNA 2) in osteosarcoma. We demonstrated that ADAR2 expression increases during osteoblast differentiation and inversely correlates with the aggressiveness of osteosarcoma cells. Interestingly, the overexpression of ADAR2 in osteosarcoma cell lines reduces their tumoral properties and promotes their differentiation in osteoblast-like cells, as shown by gene expression analysis and mineralization assays. These results were also confirmed by in vivo experiments; indeed, intratibial injection of ADAR2-overexpressing osteosarcoma cells in NSG mice resulted in less aggressive tumors compared to mice injected with pEmpty or pInactive ADAR2 E/A vector-transfected cells. To elucidate the mechanisms by which ADAR2 overexpression induces osteogenic terminal differentiation of osteosarcoma cells, we performed *RNA-seq* analysis of Saos-2 cells and identified *IGFBP7* (Insulin-like Growth Factor Binding Protein 7) as the most highly edited transcript in ADAR2-overexpressing cells. We showed that the editing activity of ADAR2 on IGFBP7 abolishes its proliferative effect on osteosarcoma cells and triggers terminal differentiation. Overall, our results indicate that ADAR2 acts as a tumor suppressor in osteosarcoma and may represent a novel therapeutic target for this aggressive pediatric tumor.

## Introduction

Osteosarcoma (OS) is a highly malignant skeletal tumor characterized by neoplastic mesenchymal cells that deposit immature osteoid matrix. Although rare, OS is the most common primary bone malignancy in children, adolescents and young adults.^1,2^ Distant metastases are frequent in this type of tumor, with a predominant tropism for the lung.^1^ Over the past three decades, several studies have explored the cytogenetic and molecular features of OS, but their diagnostic and prognostic value remains limited.^2^ Current therapeutic strategies for OS include chemotherapy and surgery aimed at removing affected bone segment and micro-metastases. Dose-intensification chemotherapy has increased the 5-year survival rate of patients with localized tumor to ~65%, compared to about 20% of patients with metastases.^3^ Thus, the identification of new therapeutic targets and strategies remains a critical unmet need, particularly for patients with chemoresistance, local relapses, or metastases.

RNA editing has emerged as an important post-transcriptional regulatory mechanism; it alters nucleotide sequences in transcripts, contributing to RNA/protein diversification in eukaryotes.^4^ One of the most common forms of RNA editing in human cells converts Adenosine (A) residues to Inosine (I) in double-stranded RNA, through hydrolytic deamination mediated by ADAR (Adenosine Deaminase Acting on RNA) enzymes; inosine is recognized as guanosine by the cellular transcriptional machinery and alters the codon meaning.^5^ This modification has a relevant impact on cell biology, by modifying RNA sequences and potentially changing protein function. Furthermore, the substitution of a single nucleotide can modulate pre-mRNA splicing sites,^5^ or it can affect RNA molecules influencing folding properties, half-life, transport, and interaction with other nucleic acids or proteins. Moreover, RNA editing can modify sequence of miRNA target sites.^6,7^ Thus, RNA editing can improve prediction, diagnosis, and possible treatment of several cancers, enabling more precise and preventive clinical care.

Three ADAR proteins are expressed in mammals (ADAR1, ADAR2 and ADAR3) and their editing activity is a powerful regulatory mechanism in tumor development and progression.^4^ Scientific evidence suggests that ADAR2 exhibits antitumor properties^8^; indeed, its reduced expression has been related to a worse prognosis, while its overexpression counteracts the neoplastic phenotype in several cancers, including high-grade gliomas, astrocytomas, esophageal squamous cell carcinoma, hepatocellular carcinoma and gastric cancer.^8–12^ The role of ADAR proteins in bone is still under investigation; it has been reported that ADAR1 regulates osteoblast differentiation by, at least in part, modulating osterix expression.^13^ Here, we characterized, for the first time, the osteogenic role of ADAR2 and we highlighted its tumor suppressor activity in OS.

## Results

### ADAR2 regulates physiological osteogenesis and bone formation

Since the role of ADAR1 has been already investigated^13^ and the expression of ADAR3 is undetectable in bone marrow-derived mesenchymal stromal cells (MSC), osteoblasts and OS cells lines (Saos-2 and 143B cells) (data not shown), we explored the role of ADAR2 in bone formation and OS progression.

**Fig. 1:**
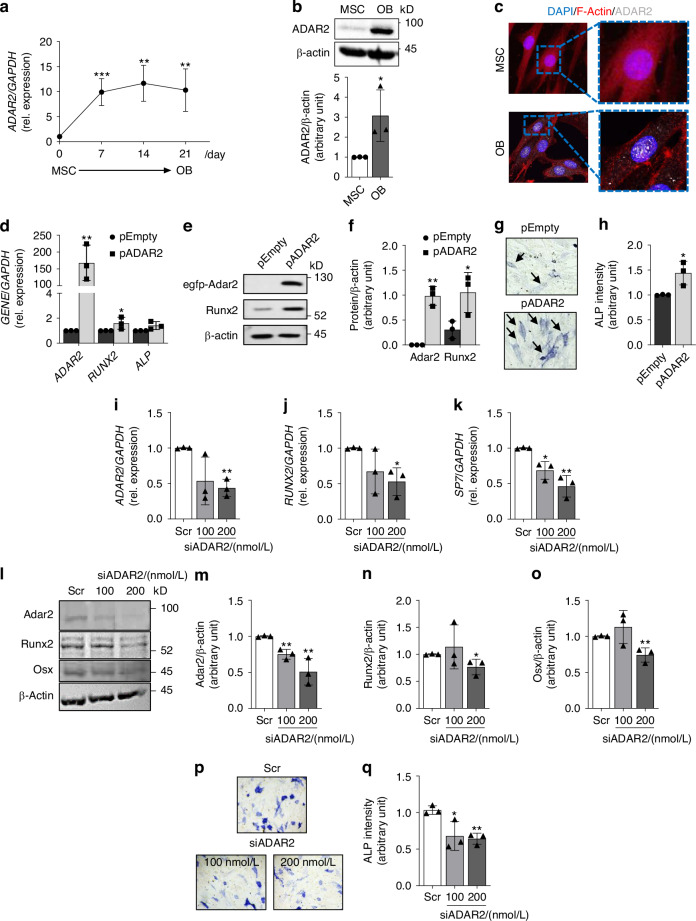
Expression of ADAR2 in mesenchymal stromal cells and osteoblasts. **a** Real-Time RT-PCR expression analysis of *ADAR2* during osteoblastogenesis. RNA was extracted from MSC (Mesenchymal Stromal Cells) at Time 0, and after stimulation with osteogenic medium (10^−7^ mol/L dexamethasone, 50 μg/mL L-ascorbic acid and 5 mmol/L β-glycerophosphate) for 7-14-21 days (osteoblasts, OB). **b** Western Blot analysis of ADAR2 in MSC and differentiated OB. *Upper panel*: representative blots. *Lower panel*: densitometric analysis. **c** Confocal microscope analysis of ADAR2 in MSC (*upper panel*) and OB (*lower panel*). Original magnification: 40X. **d** Real-Time RT-PCR expression of *ADAR2*, *RUNX2* and *ALP* of MSC nucleofected with vector overexpressing ADAR2 (pADAR2) and pEmpty vector. **e** Representative blots and **f** densitometric analysis of Adar2 and Runx2 in MSC nucleofected with pADAR2 and pEmpty vectors. **g** Representative pictures of Alkaline Phosphatase staining (black arrows indicate ALP positive areas) and **h** densitometric analysis of MSC nucleofected with pADAR2 and pEmpty vectors. **i**–**q** Healthy donor osteoblasts were treated with siRNA directed to *ADAR2* transcript (siADAR2) or with relative control siRNA (scrambled sequence, Scr). Real-Time RT-PCR expression of **i** *ADAR2*, **j** *RUNX2* and **k** *SP7* in siRNA treated osteoblasts. **l**–**o** Western Blot analysis of siRNA-treated osteoblasts. **l** Representative blots and **m**–**o** densitometric analysis of **m** Adar2, **n** Runx2 and **o** Osx. **p** Representative pictures and **q** densitometric analysis of ALP staining. Results are expressed as mean ± sd and are reported as individual data points of independent experiments. *P < 0.05; **P < 0.01; ***P < 0.001

We first assessed its expression in bone cells and its relevance in bone formation activity. Real-Time PCR (RT-PCR) and Western Blot analyses revealed low levels of ADAR2 in bone marrow-derived MSC; notably, its expression significantly increased during their differentiation into osteoblasts (Fig. 1a, b). These results were corroborated by immunofluorescence staining, which showed the characteristic nucleolar localization of ADAR2 in osteoblasts (Fig. 1c).

Overexpression of ADAR2 in MSC enhanced the expression of osteogenic marker Runx2 (Runt-related transcription factor 2) (Fig. 1d–f) and ALP (Alkaline Phosphatase) activity (Fig.1g, h).

Conversely, in healthy donor-derived osteoblasts the downregulation of ADAR2 by small interfering RNA (siRNA) reduced ADAR2 expression by about 60% (Fig. 1i, l, m) leading to decreased expression of transcription factors Runx2 (Fig. 1j, l, n) and Osx (Sp7, Osterix) (Fig. 1k, l, o), and ALP activity (Fig. 1p–q).

The results demonstrated that ADAR2 is required for osteogenic differentiation and bone formation activity in healthy cells.

### ADAR2 downregulation is associated with poor prognosis in osteosarcoma

**Fig. 2:**
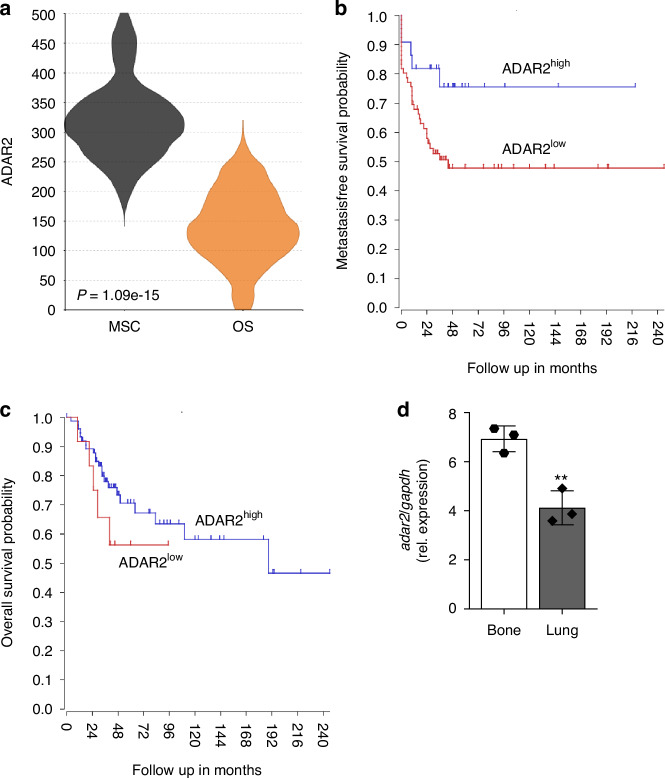
ADAR2 expression in human osteosarcoma samples from datasets. **a** MegaSampler analysis performed across public datasets (R2-http://hgserver1.amc.nl) showing the expression of ADAR2 (probes 203865_s_at) in 30 samples mesenchymal stromal cells (GSE7637) and 27 fresh frozen human osteosarcoma specimens (GSE14827). Kaplan-Meier survival analysis curves showing the **b** metastasis free survival and **c** overall survival probabilities of osteosarcoma patients depending on *ADAR2* expression (R2-http://hgserver1.amc.nl, GSE42352). **d** *ADAR2* expression in human osteosarcoma Well5 cell line in lung metastasis and bone primary tumor. Well5 cells were injected in the proximal tibia of NOD/SCID mice and RNA was extracted from bone tumor and lung metastasis. Data obtained from NCBI GEO database (GSE85537). Results are expressed as mean ± sd. **P < 0.01

Since OS arises from disrupted MSC differentiation into osteoblasts, we evaluated ADAR2 expression in OS samples and cell lines. We performed Megasampler analysis across public datasets (https://hgserver1.amc.nl/, GSE14827 and GSE7637) showing a significant reduction of ADAR2 expression in 27 human OS specimens compared to healthy donor-derived MSC (Fig. 2a). Moreover, in silico Kaplan-Meier survival analysis of high-grade OS biopsies from 84 treatment-naïve patients (GEO accession number GSE42352) revealed that low ADAR2 expression is significantly associated with reduced metastases-free and overall survival probabilities (Fig. 2b, c). Correlation analysis between ADAR2 and known prognostic gene signatures^14–16^ showed moderate positive correlations with *IGF2*, *VCAM1*, *ZFP90* and *FBXL5* expression (Fig. S1a–d). To confirm the inverse relationship between ADAR2 expression and tumor aggressiveness, we analyzed the GSE85537 dataset (NCBI GEO database) of Well5 OS cells after injection in the proximal tibias of mice^17^; interestingly, Well5 cells migrating to the lung showed a 40% decrease of *ADAR2* expression compared to cells remaining in bone (Fig. 2d). These data suggested that reduced levels of ADAR2 are associated with increased tumor aggressiveness.

### ADAR2 overexpression triggers terminal differentiation of OS cells

**Fig. 3:**
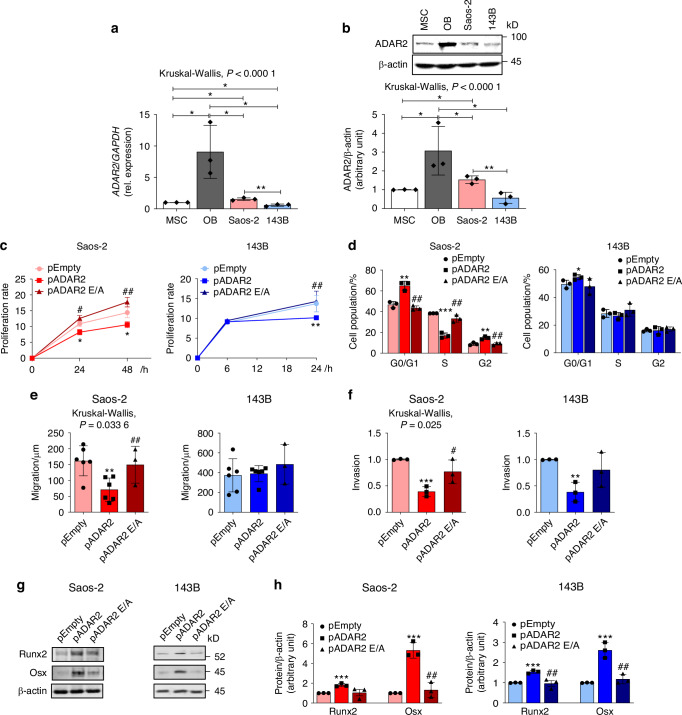
Effects of ADAR2 overexpression in osteosarcoma cell lines. **a** Real-Time RT-PCR and **b** Western Blot analysis of ADAR2 expression in MSC, osteoblasts (OB) and osteosarcoma cell lines Saos-2 and 143B. In **b** *upper panels*: representative blots; *lower panel*: densitometric analysis. **c** FACS analysis of the proliferative rate evaluated by CMAC staining and **d** cell cycle analysis of Saos-2 (*left panel*) and 143B (*right panel*) cells transfected with ADAR2-pEGFP-C3 (pADAR2), ADAR2 E/A-pEGFP-C3 (pADAR2 E/A) or Empty-pEGFP-C3 (pEmpty) vectors. ADAR2 E/A vector was generated by a single mutation in the catalytic domain of ADAR2. **e** Migration ability of transfected Saos-2 (*left panel)* and 143B (*right panel)* cells. **f** Transwell invasion assay of transfected Saos-2 (*left panel*) and 143B cells (*right panel*). **g** Representative blot and **h** densitometric analysis of Runx2 and Osx in transfected Saos-2 (*left panel)* and 143B cells (*right panel*). Results are expressed as mean ± sd and are reported as individual data points of independent experiments. *P < 0.05; **P < 0.01; ***P < 0.001; vs pEmpty transfected cells. ^#^*P* < 0.05; ^##^*P* < 0.01 vs pADAR2 transfected cells

To investigate whether ADAR2 could be involved in the terminal differentiation of OS, we took advantage of two OS cell lines with different characteristics: Saos-2, with osteoblast-like features and the ability to differentiate toward the osteogenic lineage, and 143B, very aggressive cells unable to differentiate into osteoblasts.^18–20^ Both OS cell lines displayed reduced ADAR2 expression when compared to osteoblasts; moreover, Saos-2 cells expressed increased levels of ADAR2 compared to MSC, while very low levels were detected in the most aggressive cell line 143B (Fig. 3a, b).

Indeed, to confirm that ADAR2 levels could be inversely correlated with tumor aggressiveness, Saos-2 and 143B cell lines were transfected with pEGFP vectors to overexpress the wild-type (WT) ADAR2 enzyme (pADAR2), an editing activity-deficient ADAR2 (pADAR2 E/A), harboring a Glu-to-Ala point mutation in the deaminase domain, or an empty vector (pEmpty) as control (Fig. S2a–f).

Overexpression of ADAR2 in Saos-2 and 143B cell lines reduced the proliferation rate and increased the percentage of cells in the G0/G1 cell cycle phase compared to Empty and ADAR2 E/A vector-transfected cells (Fig. 3c, d). In Saos-2 cells, ADAR2 overexpression resulted in the reduction of cells in the S phase, together with an increased cell population in the G2 phase (Fig. 3d, *left panel*). Conversely, ADAR2 E/A-overexpressing cells showed no significant alteration in the proliferation or cell cycle distribution, acting similarly to pEmpty-transfected cells (Fig. 3c, d).

Scratch assays revealed that ADAR2-overexpressing Saos-2 cells are characterized by reduced ability to close the wound gap (Fig. 3e, *left panel*). No alteration of migration was detected in 143B cells overexpressing ADAR2 (Fig. 3e, *right panel*), maybe due to the higher metastatic features of this cell line compared to Saos-2. Moreover, transwell invasion assays displayed that ADAR2 overexpression inhibited the invasive capacity of both OS cell lines (Fig. 3f).

The progressive reduction of the tumoral features of OS cells can culminate in the terminal osteoblast differentiation and in the embedding of the cells within the bone matrix they have released.

Indeed, Saos-2 and 143B cells overexpressing ADAR2 expressed higher levels of osteoblast transcription factors Runx2 and Osx (Fig. 3g, h).

**Fig. 4:**
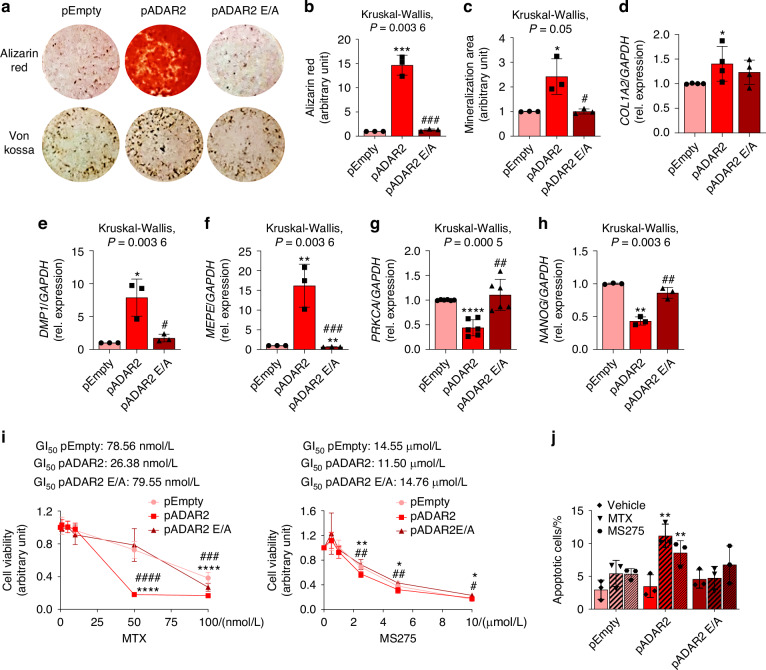
Terminal osteogenic differentiation and increased drugs susceptibility in pADAR2-transfected Saos-2 cells. **a**–**c** Mineralization assay of Saos-2 cells transfected with pADAR2, pADAR2 E/A or pEmpty vectors. **a** *Upper panels*: Alizarin Red staining; *lower panels*: Von Kossa staining. **b** Absorbance analysis of Alizarin Red staining. **c** Densitometric analysis of Von Kossa-stained area. **d**–**h** Real-Time RT-PCR expression analysis of **d** *COL1A2*, **e** *DMP1*, **f** *MEPE*, **g** *PRKCA* and **h** *NANOG*. In (**b**–**h**) results are expressed as mean ± sd and are reported as individual data points of independent experiments. **i** Cell viability analysis of transfected Saos-2 cells treated for 6 days with increasing concentrations of MTX (0, 1, 5, 10, 50 and 100 nmol/L, *left panel*) and of MS275 (0, 0.5, 1, 2.5, 5 and 10 μmol/L, *right panel*) for 2 days. The concentration of drugs able to reduce by 50% (GI_50_) cell viability is reported in the upper part of each graph. Results are expressed as mean ± sd of at least three independent experiments. *P < 0.05; **P < 0.01; ***P < 0.001; ****P < 0.000 1 vs pEmpty transfected cells. ^#^*P* < 0.05; ^##^*P* < 0.01; ^###^*P* < 0.001; ^####^*P* < 0.000 1 vs pADAR2 transfected cells. **j** FACS analysis of apoptosis of transfected Saos-2 cells treated with the GI_50_ calculated for pEmpty transfected Saos-2, or with Vehicle. Results are expressed as mean ± sd and are reported as individual data points of independent experiments. **P < 0.01 vs Vehicle treated cells

Moreover, taking advantage of the osteoblast-like features of Saos-2 cells, we found that ADAR2 overexpression in Saos-2 stimulated the production of mineralized matrix, as shown by Alizarin Red and Von Kossa stainings, revealing the formation of mineralized nodules (Fig. 4a–c). This effect was not observed in 143B cells (data not shown), as they are unable to initiate the extracellular matrix mineralization process. Consistent with terminal osteogenic differentiation, ADAR2-overexpressing Saos-2 cells showed increased expression of *Collagen type I alpha-2 chain* (*COL1A2*) (Fig. 4d), osteocyte markers such as *Dentin matrix protein 1* (*DMP1*) (Fig. 4e), and *Matrix extracellular phosphoglycoprotein* (*MEPE*) (Fig. 4f); additionally, reduced levels of *Protein Kinase Cα* (*PRKCA*) (Fig. 4g) and *NANOG homeobox* (*NANOG*) (Fig. 4h), that is involved in maintaining cell stemness, were detected when compared to pEmpty- and pADAR2 E/A-transfected cells. These results suggest that ADAR2 could be used as a therapeutic target to induce the terminal differentiation of OS cells.

However, as it has been demonstrated that differentiation therapy seems to account for long-term disease remission only when combined with standard cytotoxic agents,^21^ we tested whether ADAR2 overexpression could have a synergistic effect with chemotherapy drugs to improve complete remission and cure rates. First, we evaluated alterations in drug susceptibility in transfected Saos-2 cells. We performed in vitro tests with four different drugs, Methotrexate (MTX), MS275, PXD101 and FK228, already reported to inhibit OS cells;^22–25^ we observed that ADAR2-overexpressing cells were more susceptible to MTX (Fig. 4i, *left panel*) and MS275 (Fig. 4i, *right panel*) treatments, compared to pADAR2 E/A- and pEmpty-transfected cells, as shown by reduced cell viability. Moreover, MTX and MS275 treatments increased the percentage of apoptotic cells in ADAR2-overexpressing cells compared to Vehicle-treated cells (Fig. 4j). Although PXD101 and FK228 reduced cell viability in pADAR2-overexpressing cells compared to pEmpty-transfected cells, no significant alterations were detected between pADAR2- and pADAR2 E/A-transfected cells following treatments (Fig. S3a, b).

### ADAR2 overexpression reduces tumorigenicity and metastatic ability of OS cells in intratibially-injected mice

**Fig. 5:**
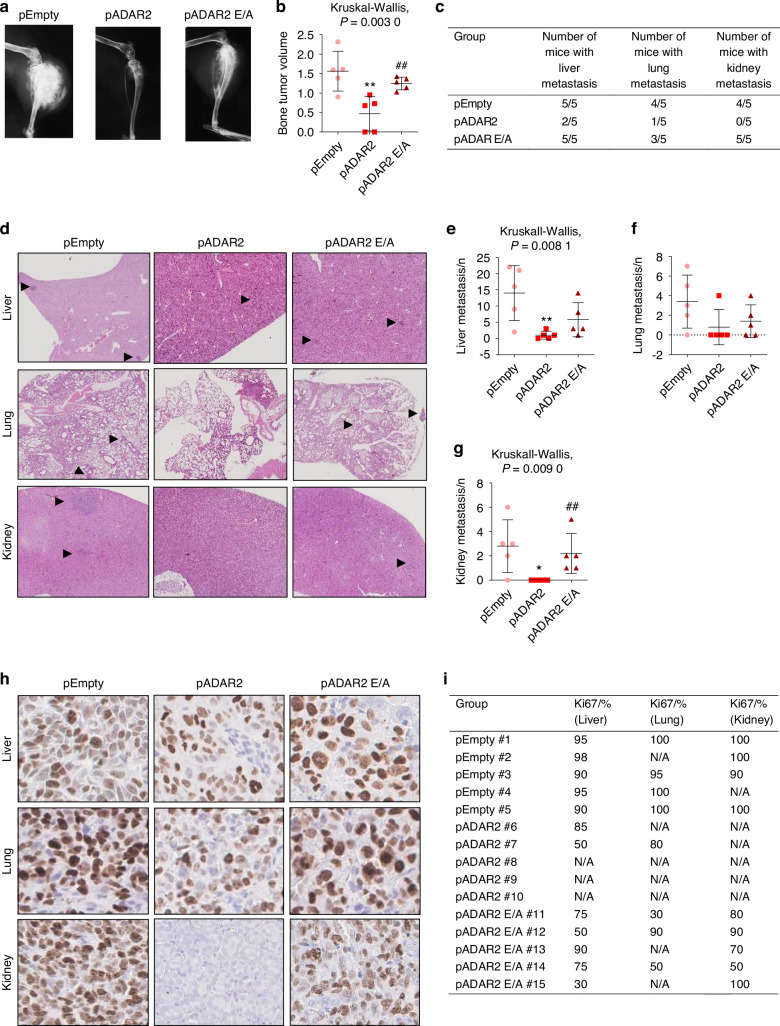
In vivo experiments. Seven-weeks-old NSG male mice were intratibially injected with Saos-2 cells transfected with pADAR2, pADAR2 E/A or pEmpty vectors; after 12 weeks animals were sacrificed. **a** Representative X-Ray pictures of primary bone tumors. **b** Quantification of the tumor volume. **c** Number of animals with metastases in liver, lungs and kidneys at sacrifice. **d** Hematoxylin/Eosin staining of liver, lungs and kidney metastases. Nodules were indicated by black arrowheads. Number of metastases in **e** liver, **f** lungs and **g** kidneys. **h** Representative pictures of immunohistochemistry and **i** quantification of Ki67 in liver, lungs and kidneys metastases. Results are expressed as mean ± sd. *P < 0.05; **P < 0.01 vs pEmpty cells injected mice. ^##^*P* < 0.01 vs pADAR2 cells injected animals

To address the relevance of ADAR2 in tumor growth in vivo, transfected Saos-2 cells were injected into the tibial medullary cavity of 7-week-old male NSG (NOD scid gamma) immunodeficient mice. After 12 weeks, X-ray analysis showed that ADAR2-overexpressing cells either failed to form tumors or developed ones that were significantly smaller and less invasive when compared to bone tumors observed in mice injected with pEmpty- or pADAR2 E/A-transfected cells (Fig. 5a–c). Interestingly, histopathological analysis revealed a reduction of liver and lung metastases in pADAR2 Saos-2-injected mice compared to the other two groups (Fig. 5c–f), while kidney metastases were not detected (Fig. 5c, d, g). Consistent with the reduced proliferation/invasive ability of ADAR2-overexpressing cells, metastatic nodules derived from these cells showed reduced Ki67 expression, as assessed by immunohistochemical analysis (Fig. 5h, i). No significant differences in tumor size or metastases formation were detected between animals injected with pEmpty- and pADAR2 E/A-transfected cells (Fig. 5), suggesting that the reduced tumorigenic capabilities depend on ADAR2-editing activity. Interestingly, less aggressive tumors characterized by reduced metastatic potential and lower Ki67 expression were also observed following in vivo intra-tibial injection of ADAR2-overexpressing 143B cells in 7-week-old male NSG immunodeficient mice, despite no differences in bone tumor formation (Figs. S4, 5).

### ADAR2 induces the osteogenic differentiation of osteosarcoma cells via editing activity on IGFBP7

**Fig. 6:**
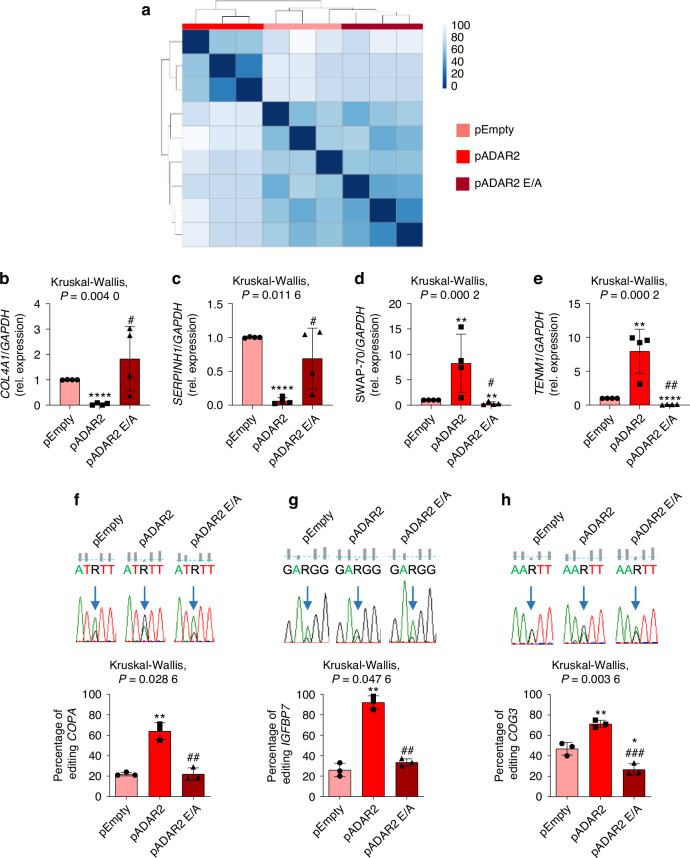
*RNA-seq* analysis. **a** Gene expression based heatmap showing the unique clusterization of ADAR2 transfected Saos-2 cells. Real-Time RT-PCR expression analysis of **b** *COL4A1*, **c** *SERPINH1*, **d** *SWAP-70* and **e** *TENM1* for transcriptional validation. **f**–**h** Editing analysis. *Upper panels*: Sequence chromatograms of the transcripts and editing levels of *COPA*, *IGFBP7* and *COG3*. Arrows indicate editing positions. *Lower panels*: percentage of editing in **f** *COPA*, **g** *IGFBP7* and **h** *COG3* transcripts. Results are expressed as mean ± sd and are reported as individual data points of independent experiments. *P < 0.05; **P < 0.01; ****P < 0.000 1 vs pEmpty transfected cells. ^#^*P* < 0.05; ^##^*P* < 0.01; ^###^*P* < 0.001 vs pADAR2 transfected cells

To deeply investigate the molecular mechanisms by which ADAR2 induces differentiation and inhibits proliferation, migration, and invasion of Saos-2 cells, we performed an *RNA-seq* analysis. ADAR2 overexpression induced a unique expression profile, whereas Saos-2 cells transfected with pEmpty and pADAR2 E/A vectors showed similar hierarchical clustering patterns (Fig. 6a). Bioinformatic analysis, performed using DESeq2 and a log_2_ FoldChange cut-off |2.0| and a statistical significance of *P* < 0.05, highlighted 633 downregulated and 386 upregulated genes between pADAR2-Saos-2 and pEmpty-cells (Tables S1, 2), and 610 downregulated and 557 upregulated genes between pADAR2- and pADAR2 E/A-transfected cells (Tables S3, 4). To validate the gene expression profiling results, comparative Real-Time RT-PCR analysis was performed on a selected subset of the most modulated genes in pADAR2-overexpressing cells (Fig. 6b–e). We confirmed the downregulation of *Collagen Type IV Alpha 1 Chain* (*COL4A1*) and *Serpin Family H Member 1* (*SERPINH1*), both previously reported to inversely correlate with tumor progression.^26,27^ Moreover, we confirmed the upregulation of *Switch-Associated Protein 70* (*SWAP-70*) and *Teneurin transmembrane protein 1* (*TENM1*) in pADAR2 cells, which either exert a pro- or an anti-tumoral effect, depending on cancer type.^28–30^

Gene ontology analysis revealed that the downregulated genes were associated with the regulation of bone mineralization in bone maturation (GO:1900157), collagen metabolic process (GO:0032963), and collagen fibril organization (GO:0030199) (Tables S5, 6), whereas the upregulated transcripts were involved in the collagen-activated tyrosine kinase receptor signaling pathway (GO:0038063) and collagen-activated signaling pathway (GO:0038065) (Tables S7, 8).

**Fig. 7:**
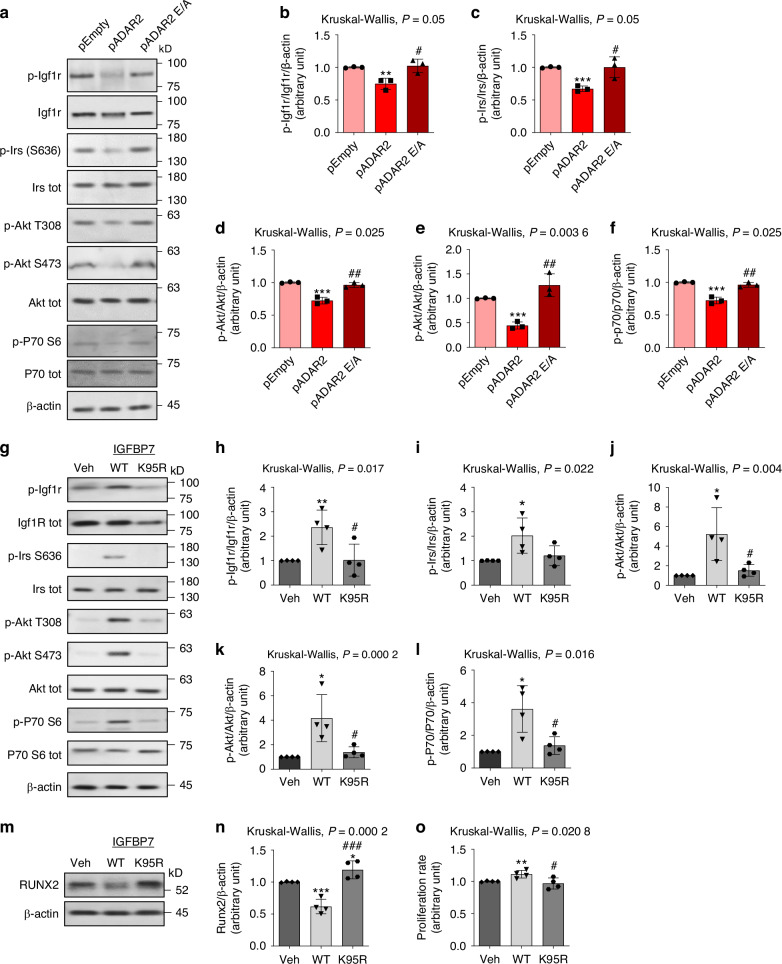
IGF1R pathway analysis. **a**–**f** Investigation of IGF1R pathway in Saos-2 cells transfected with pADAR2, pADAR2 E/A or pEmpty vectors. **a** Representative plots and **b**–**f** densitometric analysis of **b** p-Igf1r, **c** p-Irs, **d** p-Akt (T308), **e** p-Akt (S473) and **f** p-p70. Results are expressed as mean ± sd and are reported as individual data points of independent experiments. **P < 0.01; ***P < 0.00 1 vs pEmpty transfected cells. ^#^*P* < 0.05; ^##^*P* < 0.01 vs pADAR2 transfected cells. **g**–**o** Effects of the treatment with WT- or K95R-IGFBP7 on Saos-2 cell line. **g**–**l** Analysis of IGF1R pathway in Saos-2 cells treated with 2 μg/ml of WT- or K95R-IGFBP7 compared to vehicle (Veh) treated cells. **g** Representative plots and **h**–**l** densitometric analysis of **h** p-Igf1r, **i** p-Irs, **j** p-Akt (T308), **k** p-Akt (S473) and **l** p-p70. **m**, **n** Western blot analysis of Runx2 in Saos-2 cells treated with WT- or K95R-IGFBP7 compared to vehicle (Veh) treated cells. **m** Representative blot and **n** densitometric analysis. **o** Proliferation rate of Saos-2 treated with WT- or K95R-IGFBP7 compared to vehicle (Veh) treated cells. Results are expressed as mean ± sd and are reported as individual data points of independent experiments. *P < 0.05; **P < 0.01; ***P < 0.001 vs Vehicle treated Saos-2 cells. ^#^*P* < 0.05; ^###^*P* < 0.001 vs WT-IGFBP7 treated cells

Since all the results pointed toward the direct involvement of ADAR2-mediated RNA editing in the decrease of proliferation and malignancy of OS, we performed a de novo transcriptome-wide analysis for RNA editing sites using a dedicated and well-established bioinformatics pipeline based on REDItools.^31,32^ Overall, our analysis revealed 1 376 significantly edited sites in ADAR2 overexpressing cells, most of them located in 3’UTR (Untranslated region), 5’UTR, intergenic, and intronic regions. We focused on the genes that were edited in non-synonymous exonic regions, revealing ADAR2-mediated RNA editing at the following recoding sites: *COPI Coat Complex Subunit Alpha* (*COPA*, I/V), *Insulin Like Growth Factor Binding Protein 7* (*IGFBP7*, K/R) and *Component Of Oligomeric Golgi Complex 3* (*COG3*, I/V) (Fig. 6f–h, *lower panels*). Editing activity was also validated by Sanger sequencing (Fig. 6f–h, *upper panels*). Interestingly, the most edited target was *IGFBP7*, resulting in the K95R-IGFBP7 variant (Fig. 6g). Moreover, *IGFBP7* transcript was also significantly edited in ADAR2-overexpressing 143B cells as shown in Fig. S6. Alterations of IGFBP7 expression and its edited form have been associated with several cancers, as it binds to IGF1R (Insulin-like growth factor 1 Receptor) and enhances the Akt pathway.^12,33^ Interestingly, we observed reduced activation of IGF1R in ADAR2-overexpressing cells (Fig. 7a, b) and, consequently, decreased phosphorylation of IRS, Akt, and p70 S6 kinase (Fig. 7a, c–f). To elucidate whether these effects could be mediated by the activity of the K95R-IGFBP7 variant, we treated Saos-2 cells with recombinant WT and K95R-IGFBP7 for 24 h. Cells treated with WT-IGFBP7 showed activation of the IGF1R signaling pathway (Fig. 7g–l), reduced Runx2 expression (Fig. 7m, n), and increased proliferation (Fig. 7o). In contrast, K95R-IGFBP7 failed to stimulate IGF1R signaling or induce cell proliferation (Fig. 7g–l, o). Furthermore, Saos-2 cells treated with K95R-IGFBP7 were characterized by enhanced Runx2 expression compared to WT-IGFBP7 or Vehicle-treated cells (Fig. 7m, n). These results indicate that reduced tumoral features and terminal osteogenic differentiation induced by ADAR2 overexpression in OS cells could be mediated by the edited form of IGFBP7.

## Discussion

Differentiation therapy represents a novel therapeutic approach in cancer, aimed at reprogramming tumoral cells towards maturation by reactivating their differentiation program. This approach allows to transform an aggressive cancer with poor prognosis into a more treatable condition, employing agents that often display lower toxicity than conventional chemotherapy.^21^ Since OS derives from disrupted osteoblast differentiation and is characterized by the persistence of cells in undifferentiated stages, the stimulation of osteogenic differentiation could represent a promising therapeutic approach to treat this rare tumor. In this study, for the first time, we evaluated how ADAR2 overexpression rescues the osteogenic program in OS cells. Cells transfected with a vector overexpressing the active form of ADAR2 exhibited terminal osteogenic differentiation and markedly reduced tumoral properties. Indeed, ADAR2-overexpressing OS cells showed increased expression of osteogenic markers and a strong reduction in proliferation and invasiveness.

The role of ADAR2 in osteoblast differentiation has not previously been investigated, although ADAR1 has been studied in this context. In 2013, Yu et al. demonstrated that ADAR1 acts as a regulator of bone mass; post-natal conditional ADAR1 knockout mice displayed reduced bone volume/tissue volume, trabecular and cortical thickness due to impaired osteoblastogenesis.^13^ In this study, we demonstrate that ADAR2 is also required for physiological osteoblast differentiation. Indeed, we showed that ADAR2 overexpression alone in heathy-donor derived MSCs is sufficient to drive osteogenic differentiation and that a 50% reduction of ADAR2 expression in human osteoblasts is enough to reduce the expression of Runx2, Osx and ALP. Previous studies provided insights into the role of ADAR2 in cell differentiation; Ye et al. demonstrated that ADAR2 may also be important for stimulus-mediated differentiation of MSC into chondrocytes; indeed, they showed that *Atractylodes lancea* volatile oil promotes chondrogenic differentiation through a mechanism involving ADAR2-editing activity on the miR-181a-5p precursor. Moreover, it has been reported that ADAR2 silencing significantly attenuates the effect of MSC chondrogenic differentiation.^34^

ADAR2 expression resulted downregulated in human OS samples compared to healthy MSC and inversely correlated with tumor aggressiveness, displaying a moderate correlation with prognostic gene signatures. Consistently, ADAR2 overexpression in OS cells induced their terminal osteogenic differentiation, leading to decreased proliferation, migration, and invasion, and increased susceptibility to standard chemotherapeutic agents. Interestingly, in vivo experiments confirmed the ability of ADAR2 to counteract tumoral progression, as shown by reduced tumor formation and metastatic ability of ADAR2-overexpressing cells.

Global analysis and comparison of RNA editing profiles between epithelial and mesenchymal phenotypes of primary tumors across multiple cancer types revealed that knockdown of ADAR2 in epithelial cells induced an elongated spindle-like mesenchymal morphology, with loss of epithelial markers (E-cadherin and γ-Catenin) and gain of mesenchymal marker Vimentin; these results suggest that ADAR2 deficiency can promote the epithelial-to-mesenchymal transition^35^ and supporting the hypothesis of an ADAR2 regulation of metastatic process. To dissect the molecular mechanisms by which ADAR2 is involved in OS progression, we performed an *RNA-seq* analysis that revealed alterations of biological processes related to bone formation activity and bone cell differentiation. At the same time, the most modulated genes found in ADAR2-overexpressing cells were *COL4A1*, *SERPINH1*, *SWAP-70* and *TENM1*. The roles of *COL4A1*, *SWAP-70* and *TENM1* in OS have not been previously investigated. Regarding SERPINH1, both in vitro and in vivo experiments have demonstrated that it promotes proliferation and invasion of OS cells;^27^ these results are in line with the reduced expression of *SERPINH1* observed in ADAR2-overexpressing cells and their reduced tumoral features. Moreover, the *RNA-seq* analysis revealed increased ADAR2 editing activity at three recoding sites in *COPA*, *COG3 and IGFBP7*, with this last being the most edited transcript. These genes have already been described as targets of ADAR2 activity.

Although the involvement of COPA and COG3 in OS has not been described yet, in other tumors the edited COPA and COG3 isoforms exert tumor-promoting or tumor-suppressive effects depending on cancer type.^36^ In hepatocellular carcinoma, COPA switches from tumor-promoting WT-COPA to cancer suppressive I164V-COPA.^37^ At the same time, the editing of COG3 in glioblastoma cell lines significantly enhanced their invasive/proliferative behavior compared to the unedited form.^36^ Moreover, COG3 is highly edited in glioblastoma samples with poor prognosis,^36^ and reduced levels of I635V-COG3 have been found in patients with Acute Myeloid Leukemia.^38^

Regarding the role of IGFBP7 in bone, it has been demonstrated that it regulates osteogenic differentiation of MSCs *via* the Wnt/β-catenin signaling pathway, and that its overexpression in MSC accelerated bone healing in a rat tibial osteotomy model.^39^ In cancer, IGFBP7 has been reported to play different roles, acting through IGF1-dependent or independent mechanisms.^40^ For instance, in breast cancer, the overexpression of IGFBP7 inhibits cell proliferation and migration, whereas it promotes tumor progression in colon cancer. The role of K95R edited form of IGFBP7 has been poorly investigated in cancer. Chen et al. reported that ADAR2-mediated IGFBP7 editing exerts tumor-suppressive activity in esophageal squamous cell carcinoma.^12^ Moreover, they reported that K95R editing protects IGFBP7 from matriptase proteolysis; indeed, wild type IGFBP7 is partially cleaved at the 27 kD C-terminus and the 8 kD N-terminus, whereas reduced cleavage is observed for the K95R-IGFBP7.^12^ Moreover, Evdokimova et al. showed that full-length IGFBP7, and not the truncated C-terminus, binds to IGF1R and inhibits downstream Akt signaling in mouse embryonic fibroblasts.^41^ Herein, we demonstrated for the first time that K95R-IGFBP7 promotes the expression of the key osteogenic transcriptional factor Runx2. Indeed, we reported that IGFBP7 stimulates Saos-2 cells proliferation by inducing IGF1R signaling with the activation of Irs, Akt and p70, and reduces the expression of Runx2. Interestingly, compared to the WT protein, K95R-IGFBP7 treatment failed to stimulate proliferation, and enhanced the expression of Runx2. These results are in line with the alterations observed in ADAR2-overexpressing cells, in which we detected the highest percentage of IGFBP7 editing.

In conclusion, this study demonstrated that ADAR2 negatively regulates OS progression, by promoting terminal osteogenic differentiation and reducing metastatic ability, through a mechanism that involves IGFBP7 editing. Thus, ADAR2 could act as tumor suppressor in OS and the induction of its expression may represent a promising therapeutic approach also in combination with chemotherapy drugs.

## Materials and methods

### Osteoblast differentiation

MSC were purchased from Lonza (Basel, Switzerland) and plated in non-coated 75 cm^2^ tissue culture flasks at a density of 160 000/cm^2^ in complete culture medium: Dulbecco’s Modified Essential Medium (DMEM) supplemented with 10% Fetal Bovine Serum (FBS), 50 U/mL penicillin, 50 mg/mL streptomycin and 2 mmol/L L-glutamine (complete medium). Cultures were maintained at 37 °C in a humidified atmosphere, containing 5% CO_2_. After 48 h adhesion, non-adherent cells were removed, and culture medium was replaced twice a week. The osteogenic differentiation was performed by incubating cells with complete medium supplemented with 10^−7^ mol/L dexamethasone, 50 μg/mL L-ascorbic acid and 5 mmol/L β-glycerophosphate. After 3 weeks of culture, osteoblast differentiation was evaluated for ALP activity (kit #86, Sigma-Aldrich, St.Louis, MO, USA) and expression of osteoblast markers.

### Nucleofection of MSC

MSC purchased from Lonza were prepared for non-viral transfection procedures performed by 4D-Nucleofector System. Cells were electroporated following the manufacturer’s instructions. Briefly, 5 × 10^5^ cells were washed with Phosphate Buffered Solution (PBS) and resuspended in P1 Primary Cell Nucleofector^TM^ Solution (Lonza) and 2 μg of pEGFP-C3-ADAR2 (pADAR2) or control pEGFP-C3 (pEmpty) vectors. FF-104 program (4D-Nucleofector, Lonza) was used for nucleofection and electroporated cells were plated in complete medium. After 48 h cells, RNA and protein extraction or ALP stainings were performed.

### RNA interference in human osteoblasts

siGENOME^TM^ SMART pool siRNA specific for human ADAR2 and the relative control siRNA (scrambled sequence, siRNA-Scr) were designed and purchased from Dharmacon (Horizon Discovery, Cambridge, UK). Human osteoblasts were plated in 12-well plates or in 96-well plates. At ~60% confluence, cells were transfected with siRNA-ADAR2 (100 and 200 nmol/L) and the relative control siRNA-Scr (200 nmol/L) using Lipofectamine 2000 (ThermoFisher Scientific, Waltham, MA, USA) in DMEM. After 48 h, RNA and proteins were extracted and ALP activity was evaluated.

### In silico analyses

OS patients’ datasets were obtained using the software “R2: Genomics Analysis and Visualization Platform” (https://hgserver1.amc.nl/). MegaSampler analysis was performed on 30 samples of MSCs (GSE7637) and 27 human OS specimens (GSE14827). Kaplan-Meier survival and correlation analyses were evaluated in 84 and 127 OS patients, respectively (GSE42352). The expression of ADAR2 in Well5 was obtained by analyzing the NCBI GEO database GSE85537.

### Osteosarcoma cell cultures and stable transfection

Commercially available human OS cell lines Saos-2 (HTB-85) and 143B (CRL-8303) were purchased from American Type Culture Collection (ATCC, Washington, NW, USA). Saos-2 and 143B cells were cultured in complete medium at 37 °C in a humidified atmosphere (5% CO_2_). 3 × 10^5^ OS cell lines were cultured in 6-well plates for 24 h and then cells were starved (starvation medium: DMEM with 50 U/mL penicillin, 50 mg/mL streptomycin and 0.25% Bovine Serum Albumin) for 48 h before RNA and protein extraction. 3 × 10^5^ OS cell lines were cultured in 6-well plates for 24 h, then cells were transfected with 0.8 μg of pEGFP-C3-ADAR2 (pADAR2) and pEGFP-C3-ADAR2 E/A (pADAR2 E/A, the inactive form as described in Cenci et al.^9^) vectors and pEGFP-C3 (pEmpty) vector as control with Lipofectamine 2000 (ThermoFisher Scientific, Waltham, MA, USA). Cells were maintained in Geneticin antibiotic (G-418, LifeTechnologies, Carlsbad, CA, USA) selection (700 μg/mL) and sorted by BD FACSAria Cell Sorter (BD Biosciences, Franklin Lakes, NJ, USA) according to their EGFP expression to generate stable cell lines. Immunofluorescence for Fibrillarin (Abcam, Cambridge, UK. Dilution: 1:250) was performed to evaluate the nucleolar localization of ADAR2.

### Real Time RT-PCR expression analysis

Total RNA was extracted using the TriPure isolation reagent (Sigma-Aldrich) following the manufacturer’s protocol. 1 μg was reverse transcribed by SensiFAST cDNA synthesis kit (Bioline, UK) and the equivalent of 25 ng was used for Real-Time RT-PCR performed using QuantStudio 7 Pro Real-Time PCR System (ThermoFisher Scientific). The fold change in gene expression was calculated with the delta-delta-cycle threshold method.^42^ Primer sequences are listed in Table S9.

### Mineralization assay

1 × 10^5^ Saos-2 cells were seeded in 24-well plates and cultured in complete medium supplemented with 10^−7^ mol/L dexamethasone, 50 μg/mL L-ascorbic acid and 5 mmol/L β-glycerophosphate. Medium was changed twice a week, and after 3 weeks Von Kossa (Bio-Optica, Milan, Italy) and Alizarin Red (Sigma-Aldrich) stainings were performed. Densitometric analysis of mineralized area was performed by ImageJ software.

### Cell cycle analysis

Transfected Saos-2 and 143B cells were cultured for 48 h in starvation medium. Then, cells were fixed for 1 h in a cold solution of acetone/methanol (Sigma-Aldrich), washed with PBS and treated with 20 μg/mL of RNAase (ThermoFisher Scientific) and 100 μg/mL of propidium iodide (PI, Sigma-Aldrich) for 30 min at 37 °C. Cells samples were analyzed by BD FACSCanto^TM^ II (BD Biosciences) cell analyzer.

### Migration and invasion assays

1 × 10^5^ OS cell lines were cultured in a 24-well plate and the day after, a scratch in the cell monolayer was made using a sterile tip. The wound closure was measured at time 0 and after 24 h analyzing all the scratches. The wound closure was calculated as the difference between the wound dimension at time 0 and at the final time.

The invasion assays were performed using transwell insert (cellQART, Northeim, Germany). Briefly, serum-free cell suspension (75 × 10^3^ cells) was added to the upper chamber of the transwell plates coated with 200 μg/mL of Matrigel (Corning, AZ, USA). In the lower chamber, complete medium was added. After 24 h, transwells were cleaned from cells in the upper chamber, followed by staining with 0.5% crystal violet (Sigma-Aldrich) for 15 min at room temperature. Then, cells were lysed in 33% acetic acid solution and the absorbance was measured at 590 nm by SINERGY H1 microplate reader (BioTek, Lonza).

### Proliferation assay

1 × 10^5^ Saos-2 and 143B cells were seeded in 6-well plates for 24 h; then complete medium was removed, cells were washed with PBS and stained with 20 μmol/L of CellTracker Blue CMAC (chloromethyl-amino-coumarin, ThermoFisher Scientific) for 30 min at 37 °C. Cells were washed with PBS and time 0 was analyzed by BD LSRFortessa X-20 cell analyzer or treated with starvation medium for time course analysis. For Saos-2 experiments, cells were analyzed at 24 and 48 h; for 143B the analysis was performed after 6 h and 24 h.

### Pharmacological treatment

Transfected Saos-2 were treated with methotrexate, PXD101, MS275 and FK228 drugs and cell viability was assessed using XTT colorimetric assay (Roche, Switzerland). XTT absorbance values were normalized to vehicle treated cells and the concentration of every drug able to induce 50% reduction of cell viability for each experimental condition (GI_50_) was calculated from dose-response curves non-linear regression analysis by GraphPad Prism version 10.

Apoptosis and cell cycle analyses were performed using the GI_50_ obtained for pEmpty-Saos-2 cells. For apoptosis, Saos-2 overexpressing cells were seeded at 2 × 10^5^ in 12-well plates and treated with GI_50_ of pEmpty transfected cells calculated for each drug. Cells were collected after 24 h, washed twice with PBS, resuspended in 1x Binding Buffer (BD Biosciences) and stained with ANNEXIN V-APC (BD Biosciences) and Propidium Iodide before flow cytometry analyses by BD LSRFortessa X-20 cell analyzer.

### In vivo experiments and histological analyses

Animal experiments were performed in compliance with Italian laws and all procedures were IACUC approved (Protocol #125-2021-PR). Seven-week old male NSG mice were purchased by Charles River Laboratories (Wilmington, MA, USA) and maintained under controlled conditions. 1.5 × 10^6^ Saos-2 and 1 × 10^6^ 143B transfected cells were intra-tibially injected in a total volume of 10 μL of PBS. Procedures were performed in sterile conditions, with mice anesthetized with intraperitoneal mixture of ketamine (100 mg/kg) and xilazina (10 mg/kg) and cells were injected in the left tibia using a 26-G needle.

Mice were monitored every week for general symptoms and tumor development. For 143B in vivo experiments, animals were sacrificed after 3 weeks. For Saos-2 experiments, mice were sacrificed after 3 months by carbon dioxide inhalation; tumor volume was measured and calculated with the formula XY^2^/2 where X is the long axis and Y is the short axis of the tumor. Soft tissue and bones were dissected and fixed with 4% formaldehyde in PBS pH 7.4 for 48 h. X-rays were performed on the left leg after samples fixation using Faxitron MX-20 Specimen Radiography System (Faxitron X-ray Corp, IL, USA) set at 24-25 kV for 6–8 s with Kodak MIN-R2000 18 × 24 films. Soft tissues were paraffin-embedded and three-micron-thick sections were used for Hematoxylin-Eosin (H&E) staining to detect the presence of metastases and for immunoistochemical staining with Ki67 (RTU, Dako) with antigen retrieval using EDTA at pH 9. The analysis was carried out on the entire tumor represented in the section according to Nielsen et al.^43^ Slides were scanned using the NanoZoomer S60 Digital slide scanner C13210-01 (Hamamatsu Photonics, Japan) and viewed with Hamamatsu Photonics’s image viewer software (NDP.view2 Viewing software U12388–01).

### Transcriptomic and RNA editing analyses

RNA from transfected-Saos-2 cells was used for *RNA-seq* conducted by Genomix4Life Srl (Baronissi, Italy). The quality and quantity of RNA were assessed using a Qubit fluorometer (Thermo Fisher Scientific) and a TapeStation 4200 (Agilent Technologies, CA, USA), respectively.

The libraries were prepared from 50 ng of purified RNA employing the Illumina Stranded Total RNA with Ribo-Zero Plus Kit (Illumina, CA, USA) according to the manufacturer’s instructions and the Illumina NovaSeq6000 System was used to sequence the mRNA obtaining paired-end 2 × 75 bp, ~80 000 000 reads/sample.

In the preprocessing step, the raw reads in fastq format were inspected and cleaned using FASTP.^44^ The mean quality *per* base was fixed at a phred-score of 20 and reads with more than 30% of unqualified bases were removed as well as reads shorter than 55 bases. Cleaned reads were aligned with STAR (2.7.9a)^45^ using the ENCODE standard options onto a consensus version of the reference (GRCh38) human genome. The 1 000 Genomes Project VCF file with consensus SNVs and InDels were provided at the genome generation stage and the alternative alleles in this VCF have been inserted to the reference genome to create a “transformed” genome. Then, the reads were mapped to the transformed genome and the alignments were transformed back to the original coordinates. Gene expression was quantified with featureCounts^46^ using the Gencode (release 39) reference gene annotation taking advantage of the strand-oriented nature of the reads. The row counts were normalized with DESeq2^47^ and a batch effect correction was introduced with the R limma package.^48^ Differential gene expression analysis was conducted with DESeq2.

Candidate RNA editing sites were detected using a slightly modified version of Lo Giudice et al.^31^ based on the REDItools^32^ python suite and the use of a differential filtering scheme with RNA editing candidates in repetitive non-Alu regions and non-repetitive regions undergoing more stringent filters compared with those applied for sites in Alu repetitive elements. Briefly, after the first round of the REDItoolDnaRna script and subsequent filtering (sites reported in dbSNP v.151 were removed), reads harboring the variations were extracted and re-aligned using PBLAT.^49^ The putative editing sites obtained, were further subjected to a second run of REDItool excluding duplicated reads and sequences known to align on multiple genome locations by Blat. The remaining A-to-G variants were finally annotated using ANNOVAR and Gencode genes. Differential editing analysis was performed by comparing editing levels in pEmpty-Saos-2 and in cells transfected with pADAR2 E/A with unpaired Welch’s *t*-test (considered significantly edited with a *P* ≤ 0.05).

### RNA editing analysis by Sanger sequencing

For editing analysis, cDNA from transfected OS cells were used for PCR reactions for *COPA*, *COG3* and *IGFBP7* genes using the primers listed in Table S10. Products were purified using NucleoSpin Gel and PCR Clean-up (Macherey-Nagel, Germany), quantified with Nanodrop2000 (ThermoFisher Scientific) and subjected to bidirectional sequencing using BigDye Terminator v3.1 (AppliedBiosystem, CA, USA). After purification with Nucleoseq (Macherey Nagel), capillary sequencing was done on a 3500 Genetic analyzer (AppliedBiosytem). Sequences were analyzed using FinchTV (Geospiza, WA, USA). Three different samples for each condition were analyzed.

### Treatments with IGFBP7 recombinant proteins

Saos-2 cells were seeded for protein and FACS analysis in 24-well and for proliferation assay in 96-well plates. To minimize the effect of endogenous IGFBP7, cells were directly seeded in starvation medium supplemented with (i) vehicle, (ii) 2 μg/mL of commercial recombinant human IGFBP7-WT protein (R&D Systems, MN, USA) or (iii) 2 μg/mL of commercial recombinant human IGFBP7-K95R protein (R&D Systems, MN, USA). FACS and protein analyses were performed after 24 h; proliferation assay was evaluated after 96 h.

### Protein expression analysis

Cells seeded for protein analysis were washed with cold PBS and lysed using radioimmunoprecipitation assay buffer (RIPA, Sigma-Aldrich) with protease inhibitor for 30 min on ice. Proteins were resolved by SDS-Polyacrylamide gel electrophoresis (PAGE) and transferred to nitrocellulose or PVDF membranes (Biorad, CA, USA). Blots were probed with the primary antibody against Adar2 (1:250, SantaCruz Biotech, TX, USA), Osterix (1:250, Novus Biological, CO, USA) and all the other antibodies (1:1 000, Cell Signaling Technology, MA, USA). Membranes were incubated overnight at 4 °C, then washed and incubated with the appropriate horseradish peroxidase (HRP)-conjugated secondary antibody (1:5 000, Biorad) at room temperature for 1 h. Protein bands were revealed by ECL detection (Biorad) according to manufacturer’s instruction and revealed by iBright Imaging System (ThermoFisher Scientific). Densitometric analysis was performed by ImageJ software.

### Statistical analyses

Data were expressed as the mean ± sd of at least three independent experiments or 5 animals/group. Statistical analysis was performed by one-way analysis of variance, followed by the unpaired Student’s *t* test or the Mann-Whitney *U* test. A *P* ≤ 0.05 was considered statistically significant.

### Ethical approval

All procedures were performed in accordance with the guidelines approved by the Italian Ministry of Health (Protocol #125-2021-PR) and were approved by the Institute of Genetics and Biophysics (IGB) Institutional Animal Care and Use Committee (IACUC). All mice were housed in a pathogen-free barrier environment.

## Supplementary information

Supplementary Figure 1

Supplementary Figure 2

Supplementary Figure 3

Supplementary Figure 4

Supplementary Figure 5

Supplementary Figure 6

Supplementary Tables

Supplementary figure Legend

## Data Availability

The data used or analyzed during this study are included in the article and available from the corresponding author upon reasonable request.
