# Supplementary figures and images for: ADAR2 induces the differentiation of osteosarcoma cells by editing activity on IGFBP7: new implications for therapy
Source: Bone Res. 2026 Apr 3;14:38. doi: 10.1038/s41413-026-00516-6 (PMC13046735; doi:10.1038/s41413-026-00516-6)

Supplementary Figure 1

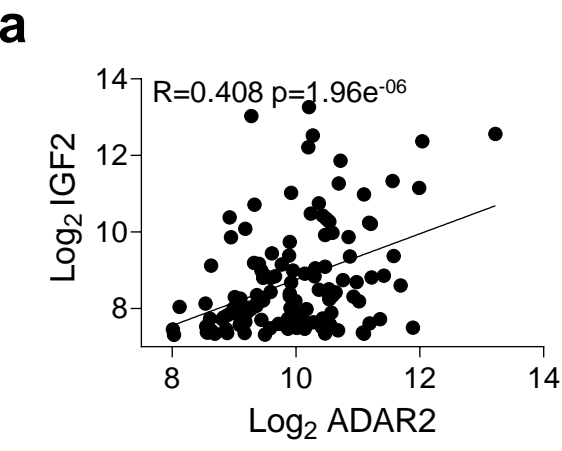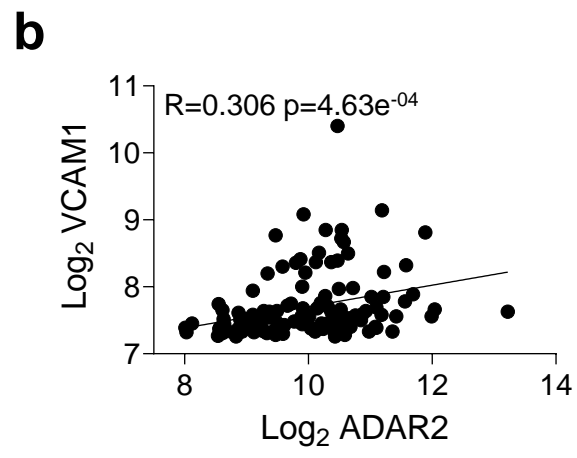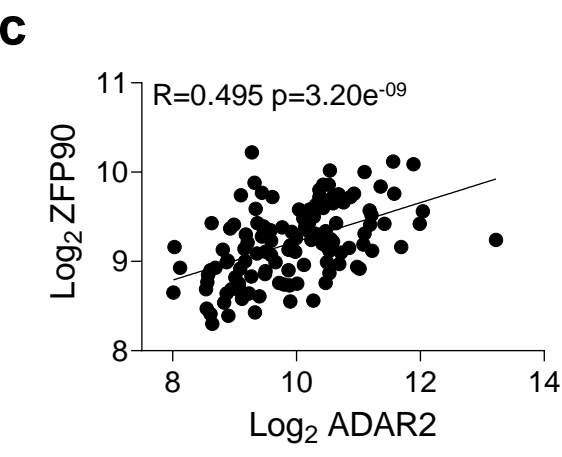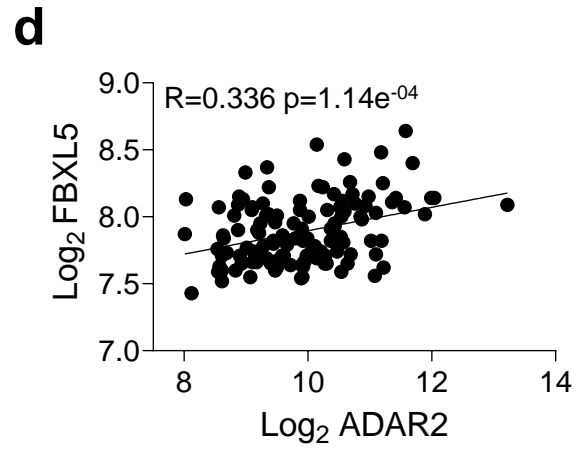

Supplement: Supplementary file 1 — Supplementary Figure 1 [file 41413_2026_516_MOESM1_ESM.pdf]

Supplementary Figure 2

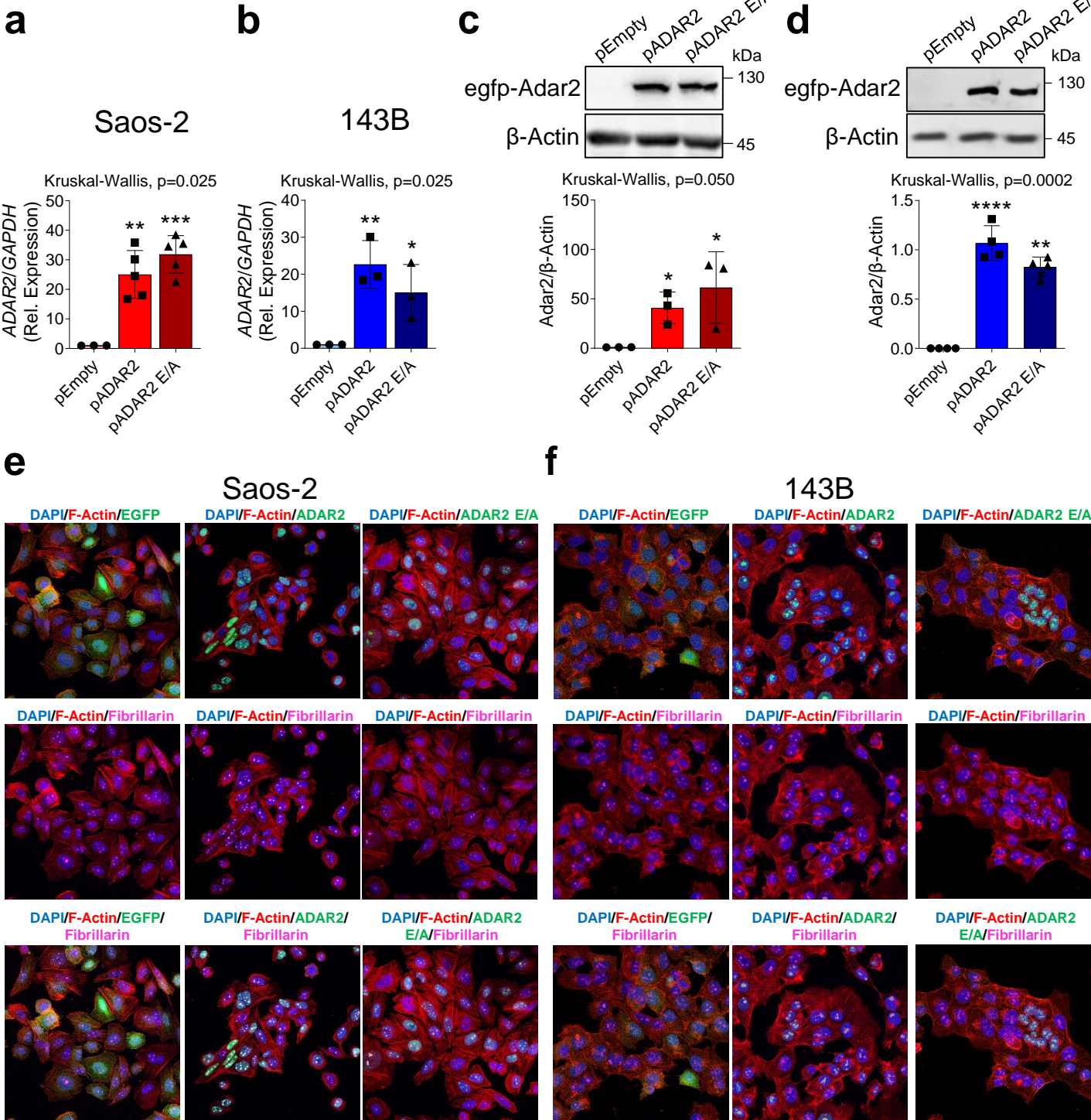

Supplement: Supplementary file 2 — Supplementary Figure 2 [file 41413_2026_516_MOESM2_ESM.pdf]

Supplementary Figure 3

**a**

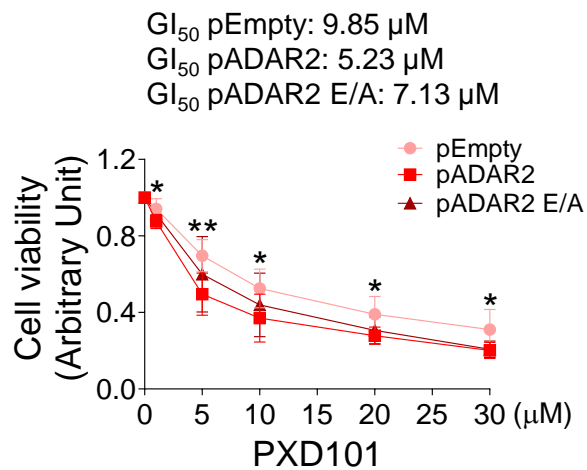

**b**

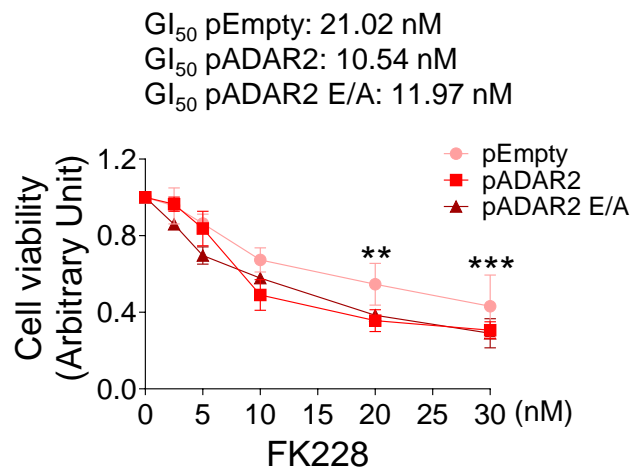

Supplement: Supplementary file 3 — Supplementary Figure 3 [file 41413_2026_516_MOESM3_ESM.pdf]

Supplementary Figure 4

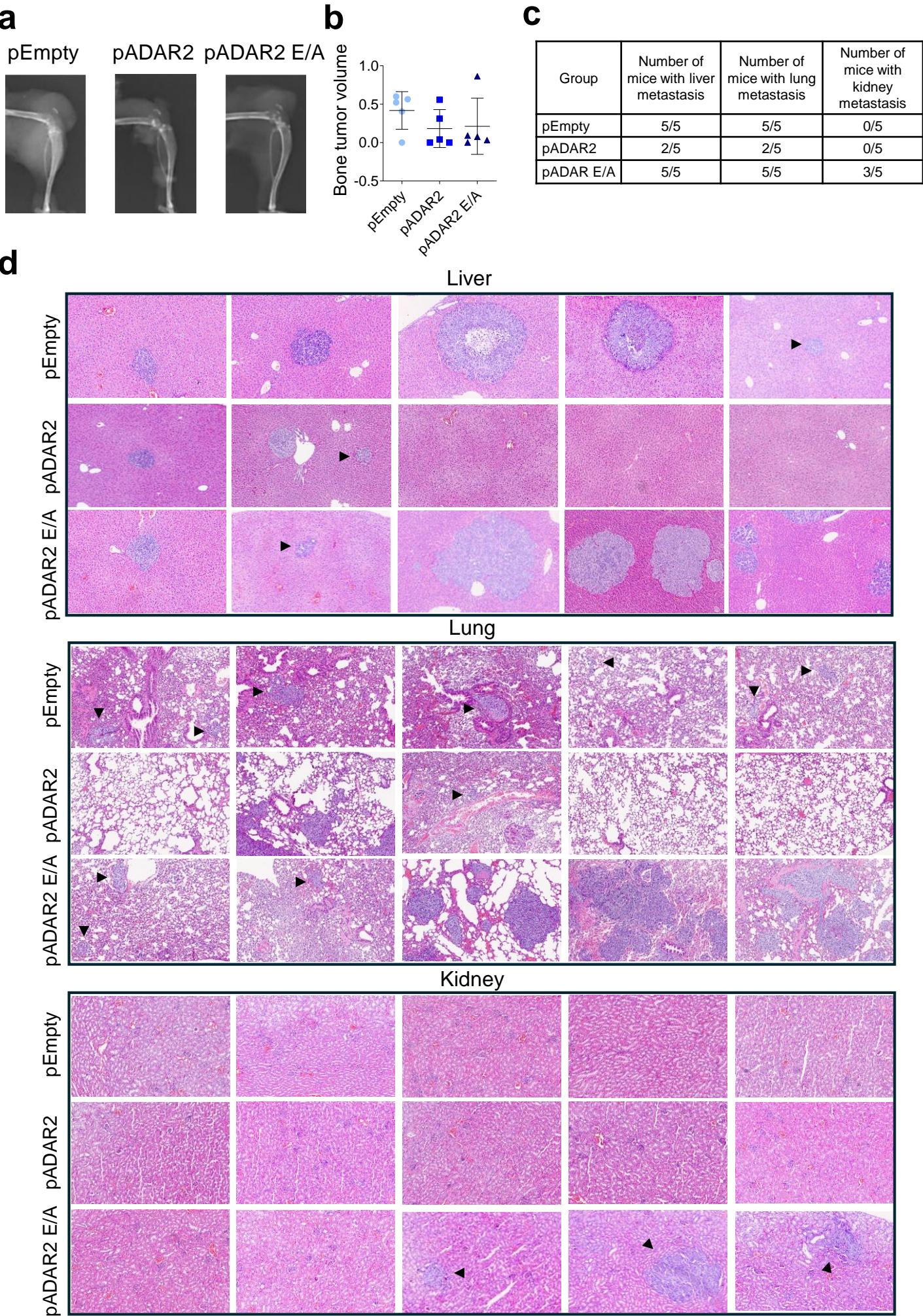

Supplement: Supplementary file 4 — Supplementary Figure 4 [file 41413_2026_516_MOESM4_ESM.pdf]

Supplementary Figure 5

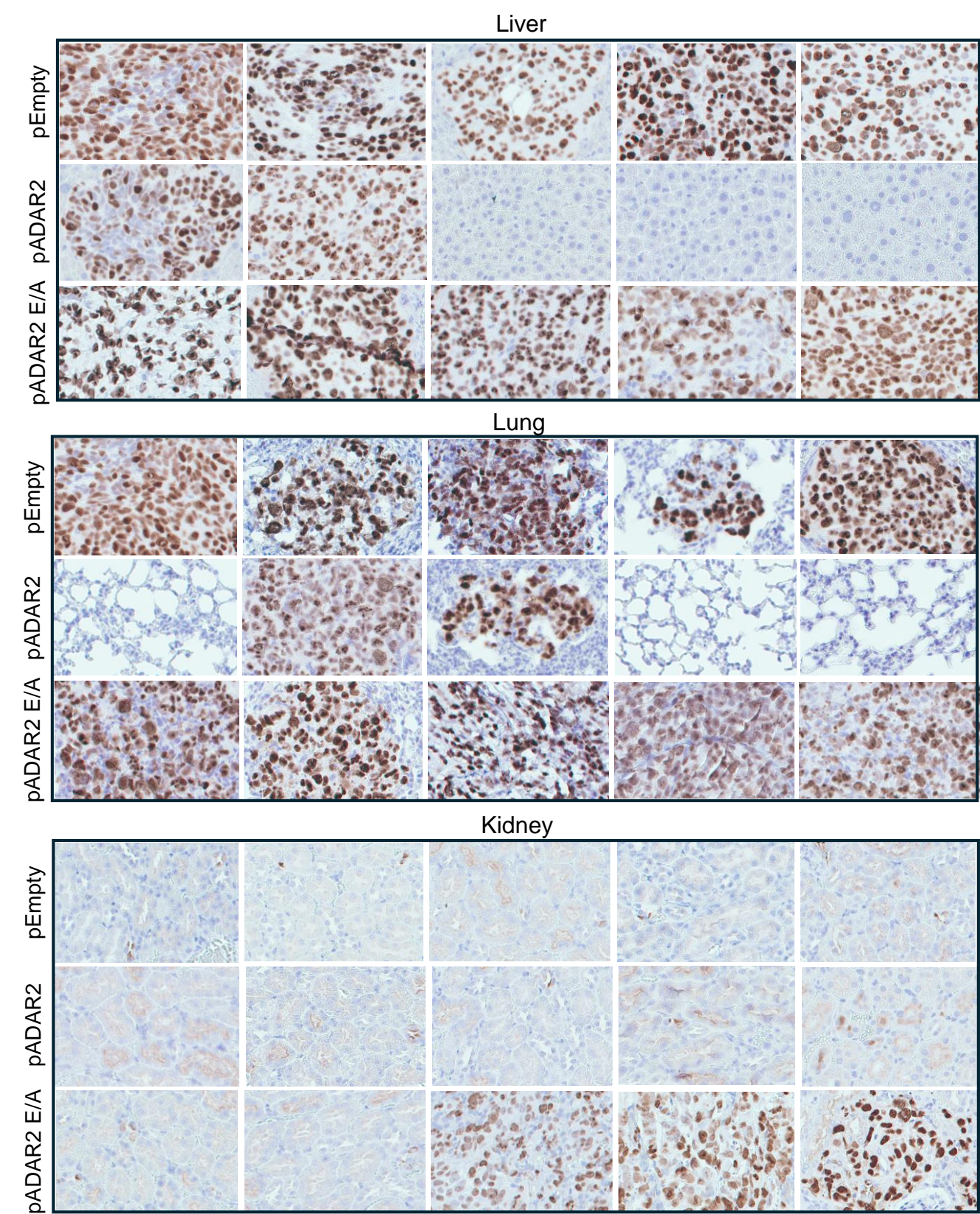

Supplement: Supplementary file 5 — Supplementary Figure 5 [file 41413_2026_516_MOESM5_ESM.pdf]

Supplementary Figure 6

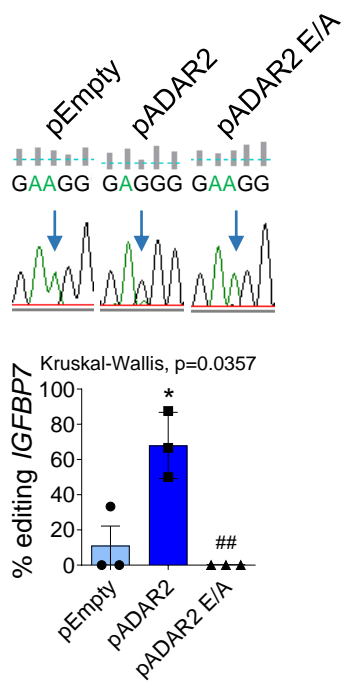

Supplement: Supplementary file 6 — Supplementary Figure 6 [file 41413_2026_516_MOESM6_ESM.pdf]
