# Supplementary material for: ADAR2 induces the differentiation of osteosarcoma cells by editing activity on IGFBP7: new implications for therapy
Source: Bone Res. 2026 Apr 3;14:38. doi: 10.1038/s41413-026-00516-6 (PMC13046735; doi:10.1038/s41413-026-00516-6)
Supplement: Supplementary file 7 — Supplementary Tables [file 41413_2026_516_MOESM7_ESM.docx]

**Supplementary Table 1. Significantly downregulated genes in Saos-2-ADAR2 compared to Saos2-Empty cells.**

| **Gene name** | **Fold Change** | **padj** |
| --- | --- | --- |
| COL4A1 | -12,084 | 8,40E-14 |
| PXDN | -11,292 | 1,70E-13 |
| PAGE2 | -10,961 | 2,86E-09 |
| ZMYND11 | -10,952 | 1,27E-23 |
| PAXIP1-AS2 | -10,892 | 5,09E-16 |
| C22orf31 | -10,892 | 3,61E-17 |
| VSIR | -10,041 | 3,48E-07 |
| FMO3 | -9,709 | 3,42E-13 |
| PLD5 | -9,552 | 3,47E-10 |
| TUBBP5 | -9,218 | 2,55E-11 |
| SELENBP1 | -9,177 | 1,21E-07 |
| PAGE5 | -9,069 | 1,67E-07 |
| HMX1 | -9,041 | 2,86E-05 |
| SERPINH1 | -8,886 | 1,54E-83 |
| BLK | -8,841 | 3,94E-07 |
| LCNL1 | -8,720 | 7,49E-03 |
| NKX2-5 | -8,691 | 1,68E-05 |
| LINC01287 | -8,456 | 2,70E-07 |
| ENSG00000287424 | -8,411 | 2,27E-07 |
| IRX2 | -8,280 | 5,61E-09 |
| GPR158 | -8,262 | 4,93E-09 |
| TRDN | -8,258 | 5,52E-08 |
| COL4A2 | -8,254 | 4,04E-14 |
| VAT1L | -8,212 | 5,90E-07 |
| DGKG | -8,174 | 5,86E-05 |
| CSMD2 | -8,161 | 2,03E-08 |
| PAXIP1 | -7,928 | 1,32E-86 |
| DMBT1 | -7,925 | 1,88E-07 |
| GPX1 | -7,909 | 2,08E-35 |
| ZNF91 | -7,815 | 1,56E-08 |
| SH2D4A | -7,744 | 1,39E-06 |
| LYPD1 | -7,731 | 1,08E-07 |
| SH3BP1 | -7,617 | 3,18E-07 |
| LINC01115 | -7,540 | 1,27E-05 |
| PTGDS | -7,501 | 2,45E-05 |
| SSX1 | -7,434 | 3,98E-05 |
| CECR7 | -7,399 | 2,54E-05 |
| MIR924HG | -7,394 | 3,34E-06 |
| TREML4 | -7,364 | 4,38E-07 |
| MIR3667HG | -7,348 | 1,16E-07 |
| ZNF429 | -7,315 | 8,80E-06 |
| ARHGAP36 | -7,314 | 6,80E-10 |
| ZNF718 | -7,221 | 2,16E-06 |
| SNED1 | -7,218 | 3,86E-43 |
| C5orf38 | -7,191 | 1,05E-05 |
| GRIP2 | -7,181 | 3,48E-09 |
| FBXL21P | -7,120 | 1,06E-05 |
| PAGE2B | -7,115 | 4,65E-05 |
| ENSG00000251511 | -7,070 | 4,06E-05 |
| MAGEB1 | -7,050 | 3,39E-22 |
| DIP2C | -6,977 | 1,96E-93 |
| NOX4 | -6,952 | 6,73E-04 |
| ENSG00000260073 | -6,949 | 3,04E-05 |
| PROK1 | -6,937 | 1,40E-05 |
| LRRN2 | -6,932 | 8,09E-05 |
| PLEKHA6 | -6,930 | 1,90E-14 |
| RPS6KA2 | -6,835 | 2,45E-11 |
| ENSG00000229588 | -6,813 | 3,71E-06 |
| AEBP1 | -6,809 | 6,86E-07 |
| LRRC37A6P | -6,792 | 4,63E-04 |
| CCN4 | -6,773 | 6,83E-119 |
| TBX1 | -6,750 | 1,68E-05 |
| NKAIN4 | -6,737 | 5,69E-05 |
| PPARGC1A | -6,714 | 9,10E-10 |
| NAA11 | -6,693 | 1,34E-04 |
| SSX5 | -6,670 | 5,31E-04 |
| TFAP2B | -6,659 | 1,16E-04 |
| ENSG00000286257 | -6,630 | 2,44E-05 |
| PCDHGA11 | -6,599 | 7,10E-92 |
| SYNDIG1 | -6,589 | 7,22E-20 |
| IGFN1 | -6,418 | 2,20E-33 |
| COLEC12 | -6,416 | 8,84E-39 |
| LINC00355 | -6,411 | 2,11E-07 |
| SLC46A2 | -6,407 | 7,22E-04 |
| CHRDL2 | -6,404 | 1,86E-03 |
| TRABD2B | -6,393 | 6,85E-04 |
| IGHVIII-38-1 | -6,370 | 4,57E-04 |
| TMPRSS15 | -6,351 | 1,23E-05 |
| C11orf16 | -6,346 | 8,61E-05 |
| CPPED1 | -6,344 | 4,34E-04 |
| MYBPH | -6,313 | 2,93E-04 |
| OR7E104P | -6,282 | 6,89E-03 |
| CSMD1 | -6,278 | 2,71E-04 |
| PROC | -6,278 | 4,59E-05 |
| TRIP4 | -6,271 | 6,34E-04 |
| IGSF1 | -6,241 | 4,71E-13 |
| COL3A1 | -6,228 | 1,03E-11 |
| PAXIP1-DT | -6,214 | 2,02E-21 |
| EMSLR | -6,180 | 1,06E-03 |
| PLAAT1 | -6,147 | 5,11E-06 |
| ENSG00000260850 | -6,137 | 5,60E-04 |
| SOX1 | -6,131 | 1,71E-03 |
| IRAG1 | -6,128 | 2,39E-35 |
| ENSG00000273183 | -6,127 | 1,95E-04 |
| MX2 | -6,114 | 8,25E-05 |
| CXCL13 | -6,091 | 2,24E-13 |
| OR56A3 | -6,072 | 2,29E-03 |
| LINC00237 | -6,070 | 7,89E-04 |
| GPX1P1 | -6,057 | 3,16E-03 |
| FIBIN | -6,004 | 1,14E-25 |
| GDA | -6,003 | 7,12E-07 |
| DEFA9P | -5,999 | 1,05E-03 |
| SSX6P | -5,988 | 3,56E-09 |
| GJB6 | -5,960 | 1,40E-03 |
| ENSG00000287046 | -5,934 | 3,26E-03 |
| RAB26 | -5,927 | 1,76E-03 |
| LIX1 | -5,912 | 6,31E-08 |
| ENSG00000286617 | -5,909 | 4,77E-03 |
| LINC02616 | -5,873 | 7,52E-06 |
| FLJ36000 | -5,852 | 6,07E-03 |
| SV2B | -5,833 | 1,62E-02 |
| NGF | -5,830 | 3,38E-13 |
| CHST8 | -5,812 | 9,51E-04 |
| ENSG00000272180 | -5,809 | 5,37E-04 |
| CAPN6 | -5,766 | 2,47E-03 |
| KANK4 | -5,759 | 3,08E-06 |
| LINC00670 | -5,756 | 4,98E-04 |
| ARMC3 | -5,755 | 9,65E-04 |
| CNTNAP2 | -5,737 | 1,28E-02 |
| ENSG00000254194 | -5,731 | 1,41E-03 |
| SAGE1 | -5,722 | 1,53E-15 |
| GRAMD2A | -5,699 | 3,69E-55 |
| ADRA2A | -5,696 | 2,58E-10 |
| NFYAP1 | -5,646 | 9,86E-03 |
| CD200 | -5,641 | 5,99E-03 |
| ENSG00000273203 | -5,639 | 6,56E-03 |
| FEZF2 | -5,637 | 3,64E-03 |
| CACNB2 | -5,598 | 3,42E-13 |
| ENSG00000238117 | -5,579 | 1,78E-02 |
| NHS | -5,571 | 4,55E-14 |
| ST8SIA2 | -5,570 | 5,90E-07 |
| GPR87 | -5,570 | 2,08E-03 |
| TSHZ2 | -5,564 | 2,76E-02 |
| CYP4F11 | -5,502 | 2,00E-04 |
| TGM2 | -5,501 | 5,44E-04 |
| ATP13A5 | -5,493 | 2,96E-02 |
| GRIN2A | -5,469 | 7,03E-03 |
| CLDN16 | -5,468 | 8,85E-03 |
| TMPRSS11B | -5,466 | 3,30E-02 |
| CHI3L1 | -5,457 | 1,80E-02 |
| EGFL6 | -5,451 | 2,63E-02 |
| ENSG00000280011 | -5,442 | 3,51E-03 |
| ENSG00000250038 | -5,435 | 1,38E-02 |
| OGN | -5,414 | 3,82E-03 |
| GUCY1A1 | -5,398 | 1,77E-07 |
| ENSG00000248837 | -5,390 | 3,37E-05 |
| ENSG00000235726 | -5,387 | 2,15E-02 |
| GPR39 | -5,385 | 2,70E-05 |
| LINC02909 | -5,353 | 7,34E-03 |
| FAM78B | -5,336 | 1,81E-64 |
| ENSG00000286280 | -5,334 | 3,22E-02 |
| ENSG00000235111 | -5,323 | 2,92E-02 |
| ARHGEF4 | -5,322 | 1,45E-02 |
| LINC00840 | -5,312 | 6,70E-03 |
| TMEM130 | -5,296 | 1,62E-05 |
| FNDC11 | -5,296 | 1,05E-02 |
| CSPG4 | -5,288 | 3,39E-22 |
| COPG2IT1 | -5,281 | 3,82E-02 |
| NPR3 | -5,266 | 2,52E-22 |
| EEF1AKMT1 | -5,242 | 1,90E-17 |
| ASB4 | -5,237 | 3,16E-02 |
| MAOB | -5,234 | 1,44E-02 |
| CHRDL1 | -5,207 | 1,45E-15 |
| ENSG00000261103 | -5,200 | 1,41E-03 |
| ENSG00000286523 | -5,170 | 7,73E-03 |
| DGCR5 | -5,169 | 2,56E-02 |
| LGALS9 | -5,161 | 2,02E-02 |
| LINC02154 | -5,154 | 1,28E-08 |
| ENSG00000248975 | -5,132 | 2,95E-06 |
| ELN | -5,128 | 6,02E-04 |
| ALDH1A1 | -5,112 | 1,82E-06 |
| TRAPPC13P1 | -5,077 | 4,17E-03 |
| CBFA2T3 | -5,053 | 1,21E-05 |
| ENSG00000287996 | -5,006 | 6,47E-03 |
| LINC02404 | -5,005 | 3,96E-02 |
| NPBWR1 | -5,000 | 9,41E-03 |
| BEGAIN | -4,992 | 3,17E-41 |
| ADORA1 | -4,990 | 1,06E-09 |
| LINC02387 | -4,980 | 1,56E-02 |
| LTF | -4,954 | 1,88E-02 |
| ENSG00000276627 | -4,952 | 3,44E-02 |
| CXCL14 | -4,946 | 1,94E-04 |
| IL6-AS1 | -4,936 | 3,95E-02 |
| NSUN5 | -4,887 | 5,10E-50 |
| SORCS2 | -4,851 | 1,33E-03 |
| PNMA8B | -4,848 | 5,51E-03 |
| MFAP2 | -4,841 | 4,02E-04 |
| PARD6G | -4,829 | 2,16E-22 |
| RGS16 | -4,812 | 3,72E-06 |
| ENSG00000256237 | -4,787 | 3,00E-02 |
| STK32B | -4,786 | 1,67E-12 |
| LINC01694 | -4,784 | 3,97E-07 |
| LINC02904 | -4,757 | 2,66E-02 |
| GGT5 | -4,732 | 5,00E-12 |
| ECEL1 | -4,703 | 1,10E-04 |
| DSG4 | -4,703 | 4,44E-03 |
| JAKMIP2-AS1 | -4,699 | 9,00E-03 |
| PRDX2 | -4,696 | 6,66E-70 |
| ENSG00000236098 | -4,695 | 4,85E-02 |
| SLC16A6 | -4,672 | 8,06E-07 |
| ENSG00000258592 | -4,662 | 1,39E-02 |
| GPR183 | -4,645 | 5,71E-22 |
| COL1A2 | -4,637 | 1,41E-38 |
| RSPO3 | -4,610 | 8,98E-05 |
| EMILIN1 | -4,598 | 6,45E-06 |
| CTSZ | -4,585 | 7,11E-14 |
| ENSG00000278000 | -4,583 | 1,36E-03 |
| C1QL3 | -4,577 | 6,69E-05 |
| ADCY2 | -4,573 | 3,21E-09 |
| EPS15P1 | -4,565 | 7,30E-08 |
| TMEM63C | -4,544 | 5,44E-04 |
| ENSG00000234692 | -4,523 | 4,60E-02 |
| SCARA5 | -4,521 | 2,65E-25 |
| GPR50-AS1 | -4,516 | 1,82E-03 |
| LINC02082 | -4,516 | 3,54E-04 |
| MLPH | -4,505 | 3,46E-02 |
| C1QL2 | -4,505 | 6,74E-05 |
| SH2D2A | -4,501 | 1,10E-02 |
| ENSG00000285722 | -4,470 | 1,41E-02 |
| TGFBI | -4,468 | 1,97E-05 |
| CMBL | -4,457 | 1,41E-02 |
| GRID2 | -4,448 | 5,51E-04 |
| ENSG00000223812 | -4,410 | 7,77E-06 |
| TEK | -4,402 | 1,33E-12 |
| SSX8P | -4,384 | 2,32E-02 |
| MAGEC1 | -4,375 | 6,30E-05 |
| PTPRE | -4,351 | 9,23E-18 |
| CLEC1A | -4,348 | 2,22E-02 |
| ZNF737 | -4,343 | 4,53E-02 |
| HMCN2 | -4,338 | 2,96E-08 |
| APCDD1L | -4,329 | 6,07E-04 |
| COL26A1 | -4,323 | 1,72E-09 |
| CHRM2 | -4,318 | 7,11E-37 |
| KCNE4 | -4,306 | 8,38E-09 |
| NKD2 | -4,303 | 6,42E-13 |
| ABI3BP | -4,303 | 1,03E-05 |
| CRLF1 | -4,300 | 1,09E-10 |
| PDGFB | -4,292 | 5,20E-09 |
| GPRC5A | -4,284 | 1,52E-05 |
| TAGAP | -4,243 | 2,19E-09 |
| MT1M | -4,228 | 4,67E-02 |
| EHBP1L1 | -4,216 | 2,04E-12 |
| ENSG00000289104 | -4,208 | 2,47E-04 |
| RAB42 | -4,204 | 4,06E-03 |
| CCND1 | -4,179 | 2,58E-21 |
| PLPP2 | -4,172 | 4,95E-03 |
| LINC01667 | -4,166 | 1,18E-02 |
| SLCO2A1 | -4,149 | 2,06E-14 |
| FOXN1 | -4,143 | 2,20E-02 |
| LMOD1 | -4,134 | 7,69E-08 |
| LINC01681 | -4,121 | 3,89E-02 |
| MIR325HG | -4,099 | 1,53E-05 |
| OLFML2B | -4,097 | 7,57E-15 |
| SUSD2 | -4,088 | 9,76E-11 |
| TRIM29 | -4,083 | 4,45E-02 |
| POSTN | -4,075 | 8,43E-24 |
| GRIN3B | -4,066 | 7,92E-13 |
| GZMB | -4,056 | 1,09E-04 |
| MDGA2 | -4,050 | 1,12E-21 |
| HSPB1 | -4,048 | 4,41E-41 |
| SUSD4 | -4,017 | 1,16E-16 |
| THBD | -4,010 | 1,34E-19 |
| LINC02864 | -4,008 | 1,54E-02 |
| ASIC2 | -4,004 | 1,05E-09 |
| ZBED2 | -3,992 | 1,76E-02 |
| DIRAS1 | -3,984 | 2,71E-04 |
| ENSG00000284616 | -3,976 | 6,14E-06 |
| GPR68 | -3,970 | 3,74E-09 |
| SCUBE1 | -3,965 | 3,05E-20 |
| ENTPD3 | -3,965 | 3,22E-12 |
| ZNF469 | -3,954 | 5,89E-05 |
| MYOM1 | -3,943 | 2,15E-56 |
| ENSG00000247134 | -3,924 | 1,49E-05 |
| ST8SIA6 | -3,924 | 8,28E-09 |
| CALHM5 | -3,921 | 1,20E-06 |
| ALDH1A3 | -3,911 | 9,66E-12 |
| ARHGAP29 | -3,903 | 1,08E-03 |
| SLC6A9 | -3,898 | 1,25E-06 |
| NLRP11 | -3,897 | 8,56E-07 |
| INSRR | -3,892 | 9,53E-13 |
| TCEAL2 | -3,878 | 2,93E-03 |
| CCR3 | -3,850 | 1,14E-04 |
| EDNRB | -3,847 | 1,60E-06 |
| ENSG00000229962 | -3,846 | 3,02E-03 |
| GALNT16 | -3,843 | 1,82E-18 |
| LSAMP | -3,842 | 2,93E-11 |
| ENSG00000223751 | -3,842 | 1,77E-04 |
| FYB2 | -3,832 | 3,23E-03 |
| SEMA3C | -3,829 | 4,21E-04 |
| JAKMIP3 | -3,824 | 1,68E-06 |
| ENSG00000287354 | -3,822 | 8,04E-03 |
| CACNG4 | -3,821 | 4,05E-13 |
| ENSG00000232053 | -3,820 | 2,46E-03 |
| FHDC1 | -3,800 | 2,33E-12 |
| HMX3 | -3,796 | 1,23E-05 |
| GDNF | -3,796 | 1,52E-17 |
| HTR1D | -3,788 | 1,20E-07 |
| IP6K3 | -3,787 | 1,90E-11 |
| MEST | -3,782 | 3,49E-03 |
| ENSG00000258919 | -3,782 | 1,14E-02 |
| ENSG00000271709 | -3,780 | 2,96E-03 |
| LINC02915 | -3,774 | 3,35E-03 |
| RGCC | -3,765 | 4,62E-06 |
| RASGEF1C | -3,763 | 1,04E-09 |
| CCR1 | -3,762 | 5,21E-03 |
| BANCR | -3,746 | 3,83E-04 |
| GRASLND | -3,738 | 5,27E-03 |
| ITGA9 | -3,699 | 2,58E-02 |
| C4B | -3,687 | 5,33E-03 |
| ARPIN | -3,677 | 1,21E-04 |
| COL22A1 | -3,669 | 4,37E-02 |
| IKZF3 | -3,652 | 3,37E-07 |
| ENSG00000248927 | -3,638 | 5,91E-03 |
| SPOCK1 | -3,637 | 6,84E-19 |
| ENSG00000269729 | -3,634 | 4,91E-02 |
| FAT2 | -3,621 | 2,53E-04 |
| DRD2 | -3,600 | 1,03E-02 |
| CT45A10 | -3,596 | 9,31E-03 |
| AFAP1L2 | -3,564 | 1,67E-12 |
| DTX1 | -3,562 | 6,27E-05 |
| C4A | -3,562 | 1,15E-04 |
| ENSG00000261549 | -3,559 | 1,29E-02 |
| ENSG00000243144 | -3,554 | 3,51E-02 |
| CDO1 | -3,550 | 8,44E-03 |
| CAMKV | -3,550 | 7,08E-04 |
| APOBEC3G | -3,541 | 4,88E-04 |
| BEAN1 | -3,536 | 3,08E-10 |
| KCNK10 | -3,536 | 2,04E-03 |
| GPR37 | -3,532 | 1,72E-02 |
| CEND1 | -3,529 | 5,08E-03 |
| GPR50 | -3,525 | 1,98E-03 |
| PDE8B | -3,525 | 3,99E-12 |
| MTMR11 | -3,520 | 1,73E-07 |
| PARD6G-AS1 | -3,510 | 1,05E-02 |
| NTRK1 | -3,509 | 4,00E-21 |
| AOAH | -3,507 | 6,99E-06 |
| ZSCAN23 | -3,505 | 6,31E-06 |
| RMDN2-AS1 | -3,500 | 5,87E-04 |
| DIPK1C | -3,500 | 1,39E-03 |
| RASGRF2 | -3,500 | 2,07E-04 |
| PRRX1 | -3,497 | 2,16E-21 |
| TP73 | -3,496 | 1,25E-11 |
| CEMIP | -3,468 | 3,75E-02 |
| GDNF-AS1 | -3,451 | 3,28E-06 |
| CDH13 | -3,443 | 1,03E-03 |
| SNTG2-AS1 | -3,436 | 6,57E-03 |
| LINC02381 | -3,430 | 3,71E-15 |
| HTRA1 | -3,421 | 1,03E-08 |
| ENSG00000229425 | -3,407 | 6,71E-05 |
| HTR1A | -3,403 | 2,22E-03 |
| TH | -3,397 | 4,74E-10 |
| CNTN1 | -3,394 | 9,11E-05 |
| PRKD1 | -3,390 | 1,28E-30 |
| TUBAP13 | -3,384 | 3,37E-02 |
| EFNA2 | -3,383 | 1,28E-02 |
| ENSG00000282024 | -3,382 | 3,83E-02 |
| RPLP0P2 | -3,374 | 7,30E-08 |
| SLN | -3,369 | 9,54E-04 |
| ANKS1B | -3,366 | 9,29E-14 |
| CSF1 | -3,355 | 1,55E-04 |
| MEGF6 | -3,349 | 6,26E-18 |
| ASB2 | -3,345 | 1,33E-08 |
| LINC00239 | -3,331 | 3,95E-05 |
| HAPLN3 | -3,327 | 1,24E-03 |
| WNT7B | -3,326 | 1,52E-02 |
| RAB7B | -3,322 | 8,53E-06 |
| ITGB4 | -3,318 | 2,92E-04 |
| WNT5B | -3,315 | 1,43E-11 |
| GAGE2A | -3,305 | 1,08E-02 |
| MAGEC3 | -3,294 | 4,68E-02 |
| LRCOL1 | -3,288 | 2,62E-02 |
| RBM24 | -3,285 | 6,24E-05 |
| CILP | -3,278 | 4,11E-07 |
| ACVR1C | -3,278 | 2,86E-32 |
| WFDC21P | -3,275 | 1,01E-02 |
| EPAS1 | -3,258 | 6,25E-18 |
| IGF1 | -3,254 | 7,43E-03 |
| TNFSF11 | -3,242 | 9,97E-05 |
| ENSG00000228061 | -3,236 | 3,63E-03 |
| ANGPTL4 | -3,235 | 1,93E-03 |
| PTPRZ1 | -3,232 | 2,13E-03 |
| ENSG00000269993 | -3,222 | 3,79E-02 |
| SERPINA1 | -3,220 | 7,55E-04 |
| HIC1 | -3,205 | 8,03E-04 |
| ATP6V1C2 | -3,197 | 1,79E-02 |
| SPATA4 | -3,192 | 2,34E-06 |
| FGD5 | -3,189 | 1,59E-03 |
| SGPP2 | -3,183 | 2,60E-09 |
| ENSG00000286616 | -3,173 | 2,00E-03 |
| PRSS56 | -3,164 | 2,39E-02 |
| NTMT2 | -3,143 | 2,94E-03 |
| FAM27C | -3,141 | 3,38E-05 |
| P2RX6 | -3,132 | 6,60E-10 |
| NKAIN3 | -3,127 | 2,94E-09 |
| IFITM1 | -3,106 | 3,20E-15 |
| LINC01500 | -3,102 | 5,81E-05 |
| ENSG00000235010 | -3,100 | 3,29E-02 |
| FAP | -3,075 | 1,25E-04 |
| EHF | -3,044 | 4,65E-07 |
| ALDH1L1 | -3,044 | 7,02E-19 |
| CASP10 | -3,034 | 2,58E-03 |
| EFEMP1 | -3,031 | 3,96E-11 |
| ARL9 | -3,026 | 1,37E-02 |
| GGT8P | -3,013 | 2,30E-03 |
| SPDYE18 | -3,005 | 1,12E-04 |
| HCN2 | -2,993 | 2,89E-13 |
| RPRM | -2,981 | 1,60E-12 |
| SULT1A1 | -2,979 | 2,63E-03 |
| CAMK1 | -2,978 | 6,91E-03 |
| CEACAM1 | -2,968 | 2,60E-03 |
| GJB2 | -2,961 | 3,20E-02 |
| ENSG00000272482 | -2,960 | 2,01E-02 |
| ELF3 | -2,937 | 3,03E-02 |
| RAB36 | -2,926 | 1,20E-07 |
| RBP1 | -2,913 | 3,90E-04 |
| SFRP2 | -2,901 | 3,88E-03 |
| ARSG | -2,898 | 2,23E-04 |
| ENSG00000250644 | -2,897 | 3,14E-02 |
| RBP7 | -2,893 | 7,08E-05 |
| CRACDL | -2,891 | 2,37E-02 |
| AKR1B10 | -2,888 | 1,27E-02 |
| CDH12 | -2,881 | 1,02E-21 |
| CACNA1C | -2,871 | 7,51E-16 |
| APCDD1L-DT | -2,869 | 2,94E-15 |
| LDB3 | -2,867 | 3,32E-17 |
| LINC02511 | -2,863 | 1,25E-02 |
| INHBB | -2,860 | 3,27E-05 |
| MXRA5 | -2,859 | 3,37E-02 |
| SPANXA1 | -2,855 | 3,03E-02 |
| TNFRSF21 | -2,839 | 1,53E-12 |
| HLA-F | -2,828 | 3,41E-30 |
| FILIP1 | -2,826 | 6,24E-11 |
| ICAM1 | -2,822 | 1,27E-02 |
| UMODL1-AS1 | -2,820 | 6,27E-04 |
| LINC01939 | -2,818 | 7,73E-03 |
| SHISA7 | -2,814 | 1,83E-03 |
| TLE2 | -2,812 | 9,71E-10 |
| ACKR3 | -2,802 | 3,97E-07 |
| LINC02302 | -2,799 | 2,20E-02 |
| METTL27 | -2,799 | 1,04E-07 |
| SPON2 | -2,797 | 8,15E-04 |
| CALHM3 | -2,796 | 5,51E-05 |
| MYOCD | -2,794 | 9,06E-11 |
| SVIL | -2,790 | 5,97E-07 |
| GUCY1A2 | -2,790 | 9,43E-10 |
| S100A5 | -2,786 | 1,50E-02 |
| PHLDA1 | -2,777 | 9,21E-06 |
| STAB1 | -2,775 | 5,92E-03 |
| RASD1 | -2,766 | 1,55E-05 |
| ENSG00000253520 | -2,758 | 3,48E-02 |
| SHANK1 | -2,757 | 1,04E-03 |
| SIM2 | -2,754 | 2,05E-02 |
| THBS2 | -2,749 | 8,65E-18 |
| CAPG | -2,747 | 2,35E-03 |
| WNT11 | -2,747 | 1,07E-13 |
| HTATIP2 | -2,740 | 7,97E-37 |
| WNT9A | -2,732 | 7,00E-09 |
| ITGA11 | -2,732 | 1,00E-07 |
| IFITM10 | -2,729 | 3,25E-06 |
| FOXS1 | -2,728 | 3,39E-02 |
| SFTA3 | -2,723 | 3,90E-05 |
| GREM1 | -2,721 | 1,45E-11 |
| NTN1 | -2,715 | 1,40E-07 |
| PDE7B | -2,711 | 2,27E-07 |
| TMEM59L | -2,705 | 1,07E-09 |
| SLC22A23 | -2,691 | 4,14E-38 |
| ENSG00000250697 | -2,684 | 2,54E-02 |
| LIPE | -2,679 | 2,04E-07 |
| ELFN1 | -2,675 | 3,18E-13 |
| CPA4 | -2,660 | 2,01E-02 |
| ABLIM3 | -2,658 | 3,10E-08 |
| BMERB1 | -2,650 | 5,25E-42 |
| GPRACR | -2,648 | 3,88E-02 |
| ENSG00000253282 | -2,644 | 5,37E-03 |
| PITPNC1 | -2,637 | 5,97E-18 |
| POU6F2 | -2,637 | 2,65E-04 |
| OMD | -2,636 | 1,13E-11 |
| RPP25 | -2,634 | 1,71E-14 |
| HEPHL1 | -2,633 | 6,50E-03 |
| RPE65 | -2,632 | 9,79E-04 |
| IL15RA | -2,626 | 7,25E-04 |
| SDK2 | -2,615 | 1,01E-03 |
| BMP7 | -2,609 | 3,95E-06 |
| PCDHB15 | -2,589 | 3,50E-09 |
| TMEM158 | -2,580 | 5,82E-04 |
| IGFL3 | -2,575 | 2,49E-02 |
| GAS6-DT | -2,575 | 1,31E-03 |
| SIPA1L2 | -2,574 | 2,30E-32 |
| DHRS3 | -2,574 | 7,40E-03 |
| BACE2 | -2,573 | 1,52E-04 |
| LRRN1 | -2,570 | 9,31E-19 |
| ENSG00000288658 | -2,563 | 4,56E-13 |
| EGFLAM-AS2 | -2,562 | 5,31E-03 |
| ENSG00000280241 | -2,562 | 1,31E-03 |
| FCMR | -2,557 | 4,92E-03 |
| RASL12 | -2,552 | 2,30E-03 |
| CPNE7 | -2,551 | 2,20E-02 |
| PLXNB3 | -2,550 | 1,70E-18 |
| F2RL3 | -2,544 | 6,88E-03 |
| CD40 | -2,541 | 3,74E-02 |
| MTUS2 | -2,539 | 1,12E-06 |
| FAM9C | -2,539 | 3,16E-02 |
| SERPINE1 | -2,539 | 3,48E-12 |
| GIPC3 | -2,535 | 2,16E-26 |
| RTN4RL1 | -2,534 | 2,81E-10 |
| NRARP | -2,532 | 7,36E-05 |
| CFAP221 | -2,531 | 4,72E-02 |
| CH25H | -2,523 | 9,86E-05 |
| CT75 | -2,517 | 4,19E-20 |
| IGFBPL1 | -2,517 | 9,14E-05 |
| MOXD1 | -2,516 | 1,31E-02 |
| PLIN2 | -2,516 | 5,98E-04 |
| NUAK2 | -2,510 | 8,96E-08 |
| BAALC | -2,509 | 1,08E-19 |
| SYNJ2 | -2,507 | 1,05E-44 |
| GRIK2 | -2,506 | 6,33E-05 |
| MIR1-1HG-AS1 | -2,501 | 1,73E-04 |
| TMEM273 | -2,492 | 9,54E-05 |
| TNFRSF1B | -2,490 | 2,46E-04 |
| AP3B2 | -2,480 | 5,73E-06 |
| HHIPL1 | -2,479 | 1,33E-04 |
| PTGER2 | -2,473 | 3,46E-13 |
| MYT1 | -2,472 | 4,85E-08 |
| ENSG00000244560 | -2,472 | 3,72E-03 |
| NFASC | -2,460 | 8,02E-04 |
| ABCA13 | -2,427 | 1,34E-03 |
| CRYZL2P | -2,425 | 2,14E-04 |
| RIPOR2 | -2,420 | 8,31E-04 |
| KCNJ2 | -2,415 | 1,44E-11 |
| NOTCH3 | -2,409 | 3,88E-08 |
| SIM1 | -2,409 | 9,88E-11 |
| ENSG00000287642 | -2,400 | 2,68E-02 |
| WASF3 | -2,398 | 1,81E-04 |
| ATP1A2 | -2,397 | 3,31E-02 |
| IHH | -2,394 | 5,54E-05 |
| NHLH1 | -2,391 | 4,21E-02 |
| SERPINA5 | -2,391 | 1,41E-03 |
| LGALS3 | -2,387 | 1,08E-07 |
| MMP17 | -2,385 | 6,65E-12 |
| ADAMTS2 | -2,376 | 4,93E-02 |
| ANXA3 | -2,373 | 6,77E-04 |
| CA12 | -2,372 | 1,30E-06 |
| SLC17A7 | -2,369 | 3,57E-14 |
| CTF1 | -2,369 | 1,00E-03 |
| PPP1R9A-AS1 | -2,368 | 2,02E-03 |
| FOXQ1 | -2,364 | 6,45E-04 |
| BMP2 | -2,362 | 1,18E-08 |
| PPP2R5CP | -2,360 | 4,75E-02 |
| FBXL22 | -2,360 | 4,93E-05 |
| PMEPA1 | -2,359 | 6,32E-12 |
| MUC1 | -2,355 | 2,74E-11 |
| IL6R | -2,351 | 5,73E-06 |
| PDGFRB | -2,347 | 4,06E-02 |
| P3H2 | -2,346 | 6,44E-04 |
| MYO7A | -2,345 | 1,95E-03 |
| PPARG | -2,344 | 1,08E-04 |
| ENSG00000238279 | -2,323 | 2,96E-02 |
| SOD3 | -2,320 | 5,14E-14 |
| SLC39A12 | -2,320 | 1,24E-03 |
| CA3 | -2,314 | 5,02E-04 |
| IL1R1 | -2,311 | 1,53E-03 |
| PAPLN | -2,296 | 1,74E-06 |
| ENSG00000287419 | -2,293 | 3,07E-02 |
| GSPT2 | -2,282 | 8,32E-03 |
| NFE2 | -2,281 | 2,96E-03 |
| ADAMTS15 | -2,277 | 4,90E-04 |
| NME4 | -2,272 | 7,07E-09 |
| EHD2 | -2,263 | 3,92E-09 |
| IFITM2 | -2,263 | 1,10E-14 |
| PLXNA2 | -2,262 | 1,33E-12 |
| RASIP1 | -2,257 | 1,47E-04 |
| GNLY | -2,245 | 3,94E-02 |
| ENSG00000276851 | -2,244 | 1,22E-02 |
| STAT4 | -2,243 | 1,45E-04 |
| CD101 | -2,242 | 2,20E-03 |
| QPRT | -2,236 | 4,35E-02 |
| ST6GALNAC5 | -2,226 | 5,94E-07 |
| GAS6 | -2,223 | 3,67E-04 |
| CRISPLD2 | -2,222 | 2,87E-07 |
| FAM189A2 | -2,216 | 6,22E-08 |
| ENSG00000266718 | -2,209 | 5,85E-03 |
| TENT5B | -2,203 | 1,08E-05 |
| TMEM190 | -2,199 | 8,88E-03 |
| ENSG00000274461 | -2,196 | 3,96E-02 |
| DCLK1 | -2,194 | 9,82E-08 |
| ADAMTS14 | -2,193 | 2,34E-02 |
| GCK | -2,192 | 2,44E-02 |
| ENSG00000285980 | -2,186 | 5,93E-03 |
| KSR1 | -2,179 | 3,66E-04 |
| OTUD7A | -2,179 | 6,13E-22 |
| KIT | -2,179 | 1,53E-08 |
| ENSG00000289520 | -2,176 | 8,34E-08 |
| MIR3681HG | -2,174 | 4,46E-02 |
| ENSG00000253508 | -2,173 | 4,62E-02 |
| LINC00705 | -2,172 | 3,28E-02 |
| HTR7 | -2,169 | 3,25E-04 |
| PPP2R5C | -2,169 | 1,08E-12 |
| GALNT5 | -2,166 | 1,03E-03 |
| ENSG00000270210 | -2,165 | 2,25E-03 |
| EYA2 | -2,162 | 1,74E-03 |
| WNT1 | -2,146 | 9,90E-03 |
| MIR646HG | -2,143 | 4,34E-07 |
| PRKCZ | -2,143 | 4,27E-03 |
| MIR548XHG | -2,138 | 2,91E-04 |
| PSTPIP2 | -2,118 | 6,41E-04 |
| FNDC5 | -2,112 | 2,76E-10 |
| LINC01497 | -2,109 | 2,53E-02 |
| GFPT2 | -2,104 | 4,61E-02 |
| GLP2R | -2,103 | 1,61E-02 |
| VSNL1 | -2,100 | 4,09E-09 |
| DOCK4 | -2,096 | 6,53E-10 |
| MVP | -2,083 | 2,85E-09 |
| SERPINF1 | -2,082 | 4,60E-02 |
| IL1RAPL2 | -2,081 | 3,20E-02 |
| SMPD3 | -2,078 | 1,05E-04 |
| DIRC3 | -2,077 | 2,08E-06 |
| MINDY4 | -2,074 | 4,77E-04 |
| SHISAL1 | -2,071 | 1,71E-14 |
| NCALD | -2,068 | 9,94E-14 |
| MMP24 | -2,062 | 1,19E-02 |
| ADPRHL1 | -2,061 | 4,25E-04 |
| RIPPLY3 | -2,060 | 1,28E-02 |
| ENSG00000259727 | -2,058 | 6,16E-03 |
| WSCD2 | -2,050 | 2,45E-05 |
| MIR1915HG | -2,043 | 2,55E-05 |
| SYT16 | -2,040 | 4,69E-02 |
| PPP4R4 | -2,038 | 6,24E-08 |
| CADPS2 | -2,035 | 2,67E-06 |
| MAEL | -2,035 | 1,16E-03 |
| CNR1 | -2,032 | 3,35E-06 |
| NPNT | -2,028 | 2,66E-03 |
| ENSG00000232524 | -2,025 | 1,42E-02 |
| CACNA1H | -2,013 | 3,15E-10 |

**Supplementary Table 2. Significantly upregulated genes in Saos-2-ADAR2 compared to Saos2-Empty cells.**

| **Gene Name** | **Fold Change** | **padj** |
| --- | --- | --- |
| COL4A5 | 8,984 | 8,74E-46 |
| ZNF391 | 8,711 | 1,58E-10 |
| GREB1L | 8,193 | 2,12E-09 |
| CYYR1 | 8,002 | 1,38E-17 |
| ELOVL2 | 7,973 | 4,10E-23 |
| ZNF204P | 7,781 | 2,64E-08 |
| NFIB | 7,754 | 4,19E-31 |
| PRSS16 | 7,593 | 6,26E-07 |
| BEND7 | 7,534 | 9,57E-15 |
| C4orf19 | 7,493 | 7,67E-10 |
| BCHE | 7,470 | 5,12E-99 |
| PGR | 7,212 | 4,23E-06 |
| PDZRN3 | 7,136 | 9,14E-06 |
| ANK2 | 7,110 | 1,86E-83 |
| LINC02830 | 7,026 | 2,24E-05 |
| CSMD3 | 6,979 | 3,90E-39 |
| SLC8A3 | 6,735 | 9,07E-04 |
| PRRG4 | 6,699 | 8,72E-08 |
| PKP2 | 6,693 | 2,16E-26 |
| ENSG00000219445 | 6,668 | 1,56E-04 |
| LINC01012 | 6,662 | 2,52E-05 |
| TAC3 | 6,621 | 1,28E-03 |
| LRRC53 | 6,474 | 5,97E-04 |
| OCA2 | 6,401 | 2,74E-03 |
| CPXCR1 | 6,219 | 1,01E-03 |
| PWWP3B | 6,218 | 5,54E-11 |
| LINC01788 | 6,216 | 1,47E-03 |
| DOK5 | 6,176 | 1,40E-04 |
| LINC02450 | 6,148 | 1,59E-03 |
| CERS4 | 6,113 | 1,11E-05 |
| NPY5R | 6,097 | 1,83E-03 |
| PDE3B | 6,079 | 3,27E-16 |
| SULT1B1 | 6,002 | 6,81E-04 |
| LRAT | 5,880 | 9,03E-03 |
| WFDC1 | 5,868 | 1,48E-12 |
| SHROOM4 | 5,757 | 7,47E-06 |
| LINC01684 | 5,748 | 3,27E-16 |
| MARCHF1 | 5,719 | 2,62E-11 |
| LINC02518 | 5,691 | 1,05E-02 |
| NPY1R | 5,655 | 2,30E-07 |
| ADAMTS12 | 5,648 | 4,50E-07 |
| ABI3 | 5,643 | 1,08E-03 |
| FGG | 5,563 | 2,21E-02 |
| SCN9A | 5,545 | 1,13E-02 |
| LGALS2 | 5,453 | 4,27E-03 |
| ENSG00000230707 | 5,408 | 3,12E-03 |
| ABCA8 | 5,390 | 2,35E-02 |
| HTR2A | 5,369 | 4,19E-02 |
| PTGER3 | 5,360 | 6,97E-08 |
| ENSG00000225606 | 5,351 | 3,81E-02 |
| RERGL | 5,285 | 1,21E-03 |
| TMEFF2 | 5,284 | 5,88E-08 |
| LRRC37A9P | 5,277 | 8,36E-04 |
| FGD3 | 5,263 | 8,27E-18 |
| PAK5 | 5,187 | 3,30E-04 |
| ENSG00000253894 | 5,164 | 1,02E-02 |
| EMB | 5,151 | 1,71E-59 |
| TMEM204 | 5,074 | 6,88E-03 |
| GABRA2 | 5,062 | 1,21E-02 |
| FLG | 5,010 | 4,39E-06 |
| ENSG00000287335 | 4,997 | 1,66E-02 |
| HPN | 4,962 | 2,94E-08 |
| TRPC4 | 4,954 | 2,12E-04 |
| MARCHF4 | 4,953 | 2,60E-02 |
| ADD2 | 4,897 | 3,67E-11 |
| ENSG00000286766 | 4,893 | 4,03E-02 |
| SMIM10L2A | 4,875 | 2,46E-04 |
| ENSG00000279668 | 4,853 | 1,27E-02 |
| NLGN1 | 4,817 | 8,16E-06 |
| LIN28B | 4,789 | 1,48E-03 |
| MMP3 | 4,788 | 2,42E-02 |
| FBLN5 | 4,786 | 1,88E-02 |
| ENSG00000236393 | 4,742 | 1,08E-02 |
| TMEM272 | 4,733 | 4,90E-02 |
| COL15A1 | 4,724 | 5,30E-03 |
| CYYR1-AS1 | 4,670 | 1,92E-02 |
| GLDC | 4,655 | 3,71E-02 |
| ADAM23 | 4,635 | 2,55E-11 |
| LBHD2 | 4,632 | 3,85E-02 |
| HMSD | 4,604 | 4,45E-02 |
| ENSG00000248538 | 4,600 | 6,67E-03 |
| NPY2R | 4,597 | 2,48E-02 |
| SLC7A3 | 4,595 | 4,52E-03 |
| LMX1B | 4,574 | 3,48E-02 |
| ENSG00000259560 | 4,559 | 2,66E-02 |
| SNORA73 | 4,555 | 3,02E-02 |
| DCAF12L2 | 4,523 | 6,65E-07 |
| ENSG00000256913 | 4,514 | 1,79E-02 |
| PCDHB17P | 4,476 | 4,86E-02 |
| LRRC70 | 4,470 | 3,73E-02 |
| ENSG00000270977 | 4,465 | 3,20E-04 |
| C14orf180 | 4,453 | 4,47E-02 |
| ENSG00000250407 | 4,453 | 3,15E-02 |
| OVAAL | 4,445 | 4,16E-02 |
| SPNS3 | 4,433 | 2,56E-05 |
| MAP3K9-DT | 4,400 | 1,33E-04 |
| HLA-DRB1 | 4,386 | 1,01E-02 |
| UGT8 | 4,379 | 3,92E-33 |
| EXOC1L | 4,373 | 4,44E-02 |
| CTNNA3 | 4,367 | 4,48E-04 |
| ENSG00000248229 | 4,338 | 4,54E-02 |
| ENSG00000230882 | 4,333 | 4,77E-03 |
| HOXB9 | 4,321 | 5,35E-07 |
| ENSG00000225867 | 4,316 | 4,51E-04 |
| GRIA3 | 4,309 | 2,06E-07 |
| CERKL | 4,302 | 2,31E-09 |
| ARHGAP15 | 4,277 | 1,45E-03 |
| EDA | 4,269 | 5,19E-18 |
| SH2D3C | 4,258 | 1,30E-05 |
| PLAG1 | 4,248 | 3,74E-26 |
| ZBTB16 | 4,207 | 2,89E-04 |
| LINC02872 | 4,196 | 1,69E-03 |
| ENSG00000227733 | 4,189 | 4,59E-02 |
| PCDH17 | 4,162 | 1,55E-24 |
| C2CD6 | 4,161 | 2,63E-06 |
| LINC02542 | 4,130 | 1,70E-04 |
| GMFG | 4,108 | 3,94E-08 |
| ENSG00000266602 | 4,102 | 2,08E-06 |
| ESR1 | 4,099 | 8,67E-03 |
| SWAP70 | 4,070 | 2,36E-13 |
| ZBED9 | 4,057 | 4,35E-25 |
| PAK3 | 4,033 | 5,43E-09 |
| ENSG00000230623 | 4,028 | 4,24E-02 |
| ADGRD1 | 4,025 | 1,22E-03 |
| CA8 | 4,017 | 7,42E-03 |
| PSG5 | 4,002 | 4,37E-02 |
| ANGPT4 | 4,001 | 9,66E-09 |
| ZNF883 | 3,976 | 1,45E-09 |
| TENM1 | 3,957 | 1,85E-55 |
| KCNMB2 | 3,935 | 9,91E-04 |
| OR8B10P | 3,935 | 5,02E-07 |
| RYR1 | 3,910 | 5,26E-04 |
| GALNT12 | 3,909 | 4,76E-10 |
| PTGIS | 3,898 | 1,63E-08 |
| AMZ1 | 3,860 | 1,36E-07 |
| TRBV26OR9-2 | 3,842 | 3,96E-08 |
| ENSG00000289591 | 3,832 | 1,32E-12 |
| LINC02211 | 3,824 | 1,38E-02 |
| SLAIN1 | 3,814 | 5,96E-10 |
| PLBD1 | 3,800 | 5,27E-20 |
| FAM107B | 3,798 | 7,14E-13 |
| ENSG00000275139 | 3,797 | 1,41E-07 |
| ENSG00000288765 | 3,748 | 5,28E-04 |
| ZFPM2 | 3,730 | 2,21E-12 |
| ENSG00000234713 | 3,729 | 6,26E-04 |
| MC4R | 3,696 | 1,92E-03 |
| DKK1 | 3,692 | 6,42E-27 |
| KCNA1 | 3,677 | 4,87E-02 |
| NOVA1 | 3,676 | 3,33E-04 |
| ENSG00000225402 | 3,660 | 2,22E-02 |
| AGGF1P1 | 3,625 | 2,88E-04 |
| CTSV | 3,610 | 3,78E-23 |
| LMO3 | 3,608 | 5,85E-03 |
| HAND2 | 3,603 | 5,42E-04 |
| CTLA4 | 3,598 | 1,58E-04 |
| HTR1F | 3,592 | 2,09E-30 |
| RDM1 | 3,544 | 6,96E-06 |
| PRELP | 3,529 | 1,86E-02 |
| ENSG00000232599 | 3,524 | 4,47E-02 |
| SCUBE3 | 3,514 | 1,28E-03 |
| GPRIN3 | 3,508 | 7,89E-03 |
| SOST | 3,505 | 6,89E-03 |
| MYL4 | 3,498 | 9,76E-05 |
| SLITRK2 | 3,487 | 1,58E-08 |
| ENSG00000253619 | 3,481 | 7,44E-04 |
| NUPR1 | 3,462 | 1,20E-09 |
| ADARB1 | 3,461 | 6,98E-23 |
| ITGBL1 | 3,450 | 2,03E-42 |
| SPARCL1 | 3,432 | 2,00E-07 |
| OR8B9P | 3,420 | 3,11E-12 |
| PDE6G | 3,417 | 1,20E-08 |
| NEGR1 | 3,415 | 1,41E-19 |
| STXBP5L | 3,411 | 7,52E-07 |
| RYR2 | 3,359 | 1,66E-04 |
| MEPE | 3,358 | 2,04E-13 |
| PCDH11X | 3,346 | 1,14E-03 |
| FUT1 | 3,340 | 1,07E-02 |
| EPB41L3 | 3,338 | 3,07E-03 |
| ENSG00000249001 | 3,324 | 2,14E-08 |
| IBSP | 3,308 | 5,11E-25 |
| SNAP91 | 3,307 | 5,22E-09 |
| LINC02152 | 3,284 | 1,53E-02 |
| ZNF804A | 3,277 | 1,14E-18 |
| BGN | 3,251 | 1,47E-02 |
| TRPV4 | 3,245 | 1,39E-02 |
| RIPOR3 | 3,242 | 3,86E-10 |
| EML5 | 3,238 | 8,44E-24 |
| ENSG00000223563 | 3,236 | 1,04E-03 |
| PARM1 | 3,230 | 1,28E-03 |
| NMU | 3,221 | 6,43E-15 |
| SCN3A | 3,218 | 3,51E-03 |
| LRRC37A4P | 3,216 | 4,49E-17 |
| TRPM6 | 3,207 | 2,14E-06 |
| ID4 | 3,206 | 4,65E-03 |
| PRKG1-AS1 | 3,201 | 2,74E-05 |
| ENSG00000229751 | 3,200 | 2,73E-02 |
| ESRP1 | 3,181 | 4,61E-02 |
| ANKFN1 | 3,179 | 1,54E-07 |
| PKHD1L1 | 3,178 | 2,10E-02 |
| ASXL3 | 3,175 | 2,05E-05 |
| SCIN | 3,175 | 8,66E-19 |
| MYOZ3 | 3,171 | 4,26E-04 |
| ZNF385B | 3,170 | 3,44E-11 |
| DNAH10 | 3,158 | 9,93E-07 |
| MAPK4 | 3,152 | 2,39E-04 |
| ENSG00000260947 | 3,131 | 1,56E-25 |
| RDH12 | 3,122 | 1,49E-02 |
| COL8A1 | 3,108 | 1,68E-14 |
| PDE11A | 3,096 | 4,36E-08 |
| ANKRD18DP | 3,094 | 1,72E-02 |
| ENSG00000287063 | 3,088 | 8,67E-03 |
| LINC02824 | 3,082 | 1,89E-02 |
| H3C1 | 3,078 | 4,07E-02 |
| RYR3 | 3,075 | 7,32E-06 |
| PTENP1-AS | 3,074 | 3,67E-11 |
| LNCOG | 3,063 | 4,38E-02 |
| GPC3 | 3,045 | 3,92E-09 |
| ENSG00000239828 | 3,030 | 1,14E-08 |
| SFRP1 | 3,017 | 7,05E-09 |
| ENSG00000250049 | 3,012 | 3,90E-12 |
| CLIC6 | 3,007 | 1,53E-14 |
| L1CAM | 2,993 | 1,29E-02 |
| LYPD6B | 2,991 | 3,57E-12 |
| MBL2 | 2,980 | 3,80E-09 |
| ENSG00000260949 | 2,974 | 2,77E-02 |
| ENSG00000254039 | 2,973 | 4,80E-02 |
| SERPINB7 | 2,955 | 2,86E-02 |
| PLBD1-AS1 | 2,940 | 1,21E-04 |
| PGAP4 | 2,928 | 5,49E-11 |
| OASL | 2,927 | 4,36E-06 |
| ENSG00000285561 | 2,921 | 1,31E-03 |
| GATA6 | 2,916 | 4,34E-04 |
| ADGRL3 | 2,888 | 9,66E-09 |
| SLC8A1-AS1 | 2,878 | 2,50E-04 |
| KRT18P10 | 2,871 | 3,65E-02 |
| TEX15 | 2,842 | 2,35E-05 |
| LINC00402 | 2,837 | 1,11E-02 |
| ENSG00000276384 | 2,830 | 1,09E-02 |
| CFHR3 | 2,816 | 9,43E-03 |
| CDH7 | 2,816 | 3,89E-05 |
| TMEM30B | 2,795 | 1,17E-02 |
| SYK | 2,792 | 5,07E-19 |
| ENSG00000235070 | 2,790 | 3,86E-02 |
| KCNJ8 | 2,778 | 5,69E-11 |
| ENSG00000254366 | 2,761 | 5,61E-03 |
| C9orf153 | 2,761 | 3,25E-02 |
| WNK2 | 2,752 | 9,95E-05 |
| SLITRK3 | 2,745 | 1,08E-07 |
| RBP4 | 2,732 | 1,21E-06 |
| ENSG00000253164 | 2,721 | 1,10E-02 |
| CGB5 | 2,719 | 8,75E-03 |
| LHX8 | 2,715 | 3,58E-16 |
| COL4A6 | 2,710 | 2,39E-04 |
| SHISA9 | 2,708 | 3,39E-02 |
| LAMB1 | 2,703 | 1,25E-12 |
| PCDHB3 | 2,695 | 2,37E-06 |
| GUSBP5 | 2,694 | 1,69E-03 |
| ENSG00000268926 | 2,677 | 3,14E-03 |
| INHBE | 2,672 | 3,31E-06 |
| KCND3 | 2,668 | 9,51E-03 |
| ENSG00000285407 | 2,664 | 1,67E-03 |
| DCHS2 | 2,661 | 5,24E-11 |
| CYP4F26P | 2,651 | 6,60E-03 |
| JAM2 | 2,645 | 1,07E-13 |
| IGF2BP1 | 2,644 | 1,89E-14 |
| ENSG00000286541 | 2,641 | 1,13E-02 |
| CCDC198 | 2,622 | 8,26E-10 |
| KIF5C | 2,616 | 4,28E-15 |
| PPP1R14C | 2,611 | 7,64E-03 |
| PCP4L1 | 2,611 | 1,16E-04 |
| HOXB13 | 2,606 | 3,28E-08 |
| TRIM58 | 2,569 | 2,49E-02 |
| ANO1 | 2,559 | 2,82E-03 |
| PERP | 2,555 | 3,13E-05 |
| ERG | 2,553 | 1,93E-08 |
| MPZL3 | 2,548 | 1,85E-03 |
| KISS1 | 2,543 | 1,63E-08 |
| ASNSP1 | 2,541 | 2,80E-07 |
| LINC02232 | 2,534 | 1,24E-02 |
| LRRC63 | 2,531 | 1,40E-02 |
| MAPT | 2,522 | 1,66E-03 |
| JAK3 | 2,509 | 1,44E-03 |
| S1PR3 | 2,492 | 2,34E-06 |
| PTPRO | 2,488 | 1,35E-03 |
| FAM162B | 2,487 | 2,10E-02 |
| BEND4 | 2,480 | 2,52E-06 |
| PRSS12 | 2,477 | 8,84E-10 |
| CHAC1 | 2,477 | 1,23E-08 |
| FGL1 | 2,465 | 8,17E-04 |
| MAK | 2,461 | 2,42E-03 |
| HTRA3 | 2,457 | 3,10E-02 |
| CHGA | 2,443 | 1,18E-02 |
| NAALAD2 | 2,443 | 1,11E-12 |
| ENSG00000286677 | 2,429 | 4,46E-02 |
| ITGA2 | 2,413 | 6,39E-08 |
| MCOLN2 | 2,403 | 9,62E-06 |
| DCLK3 | 2,402 | 3,28E-02 |
| ENSG00000267260 | 2,399 | 4,68E-03 |
| GSC | 2,398 | 4,94E-03 |
| FXYD5 | 2,394 | 3,13E-13 |
| KRT18 | 2,390 | 1,37E-28 |
| NUDT10 | 2,383 | 3,92E-04 |
| ENSG00000280604 | 2,379 | 3,88E-03 |
| DHRS2 | 2,377 | 1,97E-02 |
| PRL | 2,376 | 7,31E-03 |
| HOOK1 | 2,370 | 7,08E-24 |
| CORT | 2,369 | 1,78E-05 |
| BCYRN1 | 2,354 | 2,47E-02 |
| ENSG00000227482 | 2,353 | 4,35E-02 |
| DLX3 | 2,352 | 2,80E-05 |
| MATN4 | 2,327 | 6,18E-03 |
| S1PR1 | 2,320 | 9,87E-06 |
| KLHL4 | 2,315 | 2,99E-27 |
| NR5A2 | 2,314 | 4,00E-05 |
| A2M-AS1 | 2,311 | 3,87E-05 |
| THNSL2 | 2,309 | 2,29E-04 |
| PTPRN | 2,308 | 4,13E-09 |
| RNF180 | 2,308 | 1,46E-02 |
| HAS2 | 2,304 | 1,13E-05 |
| ENSG00000261815 | 2,303 | 3,99E-02 |
| ENSG00000256615 | 2,292 | 1,69E-03 |
| METTL24 | 2,291 | 5,51E-03 |
| ENSG00000232936 | 2,287 | 1,92E-02 |
| ADCY5 | 2,285 | 7,72E-05 |
| DKKL1 | 2,278 | 4,65E-02 |
| DIO2-AS1 | 2,271 | 3,29E-02 |
| MTARC1 | 2,259 | 5,42E-03 |
| LRRC37A7P | 2,257 | 4,62E-02 |
| ZNF521 | 2,255 | 4,84E-06 |
| QRFPR | 2,252 | 4,99E-03 |
| ULBP1 | 2,250 | 2,08E-08 |
| ENSG00000231829 | 2,244 | 4,86E-05 |
| ROBO3 | 2,243 | 9,80E-05 |
| SP9 | 2,238 | 3,58E-02 |
| ENSG00000265702 | 2,235 | 5,49E-03 |
| THAP5P1 | 2,230 | 5,14E-03 |
| IL21R | 2,229 | 1,41E-02 |
| INPP5D | 2,222 | 2,88E-02 |
| MAP3K9 | 2,213 | 1,43E-07 |
| TSPAN33 | 2,212 | 2,48E-04 |
| PTENP1 | 2,211 | 2,39E-04 |
| DCX | 2,201 | 2,40E-07 |
| MB | 2,190 | 2,61E-04 |
| ENSG00000229116 | 2,183 | 3,82E-02 |
| NCAM2 | 2,182 | 1,39E-20 |
| PLA2R1 | 2,182 | 1,59E-03 |
| NFATC1 | 2,180 | 2,65E-07 |
| CNTN5 | 2,174 | 3,15E-03 |
| ENSG00000282849 | 2,168 | 1,20E-05 |
| ENSG00000236461 | 2,160 | 1,81E-02 |
| TP63 | 2,148 | 2,36E-03 |
| GLUD2 | 2,147 | 1,17E-06 |
| PANX3 | 2,146 | 3,90E-09 |
| ENSG00000255446 | 2,140 | 2,40E-06 |
| SEPTIN4-AS1 | 2,140 | 2,76E-02 |
| KCNK5 | 2,134 | 1,78E-04 |
| ENSG00000283064 | 2,129 | 1,00E-02 |
| CALB2 | 2,120 | 2,28E-03 |
| LINC02901 | 2,119 | 2,70E-03 |
| ZNF695 | 2,114 | 2,18E-03 |
| ENSG00000258168 | 2,108 | 1,21E-02 |
| MYH7 | 2,106 | 2,56E-02 |
| PURG | 2,105 | 6,40E-03 |
| NMNAT3 | 2,105 | 1,79E-04 |
| LINC01549 | 2,104 | 1,03E-02 |
| ICAM2 | 2,094 | 1,14E-02 |
| MSX2 | 2,093 | 1,66E-03 |
| ENSG00000279692 | 2,090 | 3,22E-04 |
| CYP27C1 | 2,079 | 1,44E-02 |
| COL12A1 | 2,076 | 1,69E-09 |
| ZNF578 | 2,072 | 2,16E-06 |
| MS4A4A | 2,066 | 4,27E-03 |
| ENSG00000286449 | 2,064 | 3,29E-04 |
| ENSG00000232667 | 2,063 | 4,36E-02 |
| HLA-DRA | 2,056 | 7,32E-03 |
| CACHD1 | 2,055 | 4,38E-05 |
| ENSG00000287097 | 2,052 | 7,75E-03 |
| JAM3 | 2,047 | 5,16E-03 |
| ADM2 | 2,047 | 5,53E-04 |
| DNER | 2,041 | 4,49E-02 |
| PKDCC | 2,034 | 1,33E-04 |
| LINGO2 | 2,024 | 2,66E-04 |
| LPL | 2,015 | 1,78E-05 |
| ENSG00000237807 | 2,014 | 1,09E-05 |
| KRT19P2 | 2,005 | 4,70E-02 |
| GDF6 | 2,002 | 1,33E-03 |

**Supplementary Table 3. Significantly downregulated genes in Saos-2-ADAR2 compared to Saos2-E/A cells.**

| **Gene Name** | **Fold Change** | **Padj** |
| --- | --- | --- |
| COL4A1 | -13,419 | 3,92E-17 |
| GABRB2 | -13,311 | 1,29E-24 |
| ZMYND11 | -11,524 | 2,51E-26 |
| VSIR | -11,351 | 3,53E-09 |
| PAXIP1-AS2 | -11,077 | 1,06E-16 |
| PXDN | -10,542 | 6,72E-12 |
| BLK | -10,374 | 8,49E-10 |
| LCNL1 | -10,060 | 1,17E-03 |
| PRPH | -9,679 | 6,10E-05 |
| SERPINH1 | -9,613 | 5,86E-98 |
| ZNF91 | -9,603 | 7,56E-13 |
| TRDN | -9,398 | 2,34E-10 |
| ZNF429 | -9,370 | 2,01E-09 |
| NKX2-5 | -9,158 | 3,77E-06 |
| SELENBP1 | -9,137 | 1,15E-07 |
| GPX1 | -8,970 | 6,57E-46 |
| COL4A2 | -8,888 | 1,66E-16 |
| DGKG | -8,847 | 7,95E-06 |
| TRIP4 | -8,769 | 2,16E-07 |
| ZNF718 | -8,732 | 2,34E-09 |
| CXCL14 | -8,725 | 5,42E-13 |
| GPR158 | -8,601 | 7,21E-10 |
| LYPD1 | -8,515 | 2,29E-09 |
| MYBPH | -8,437 | 2,48E-07 |
| PAGE2 | -8,435 | 9,67E-06 |
| PAGE5 | -8,403 | 1,37E-06 |
| SH2D4A | -8,398 | 8,66E-08 |
| NOX4 | -8,277 | 2,14E-05 |
| AEBP1 | -8,175 | 7,50E-10 |
| LINC01115 | -8,127 | 1,41E-06 |
| PTGDS | -8,104 | 3,03E-06 |
| TREML4 | -8,040 | 1,67E-08 |
| SSX1 | -8,030 | 5,38E-06 |
| ENSG00000287424 | -7,964 | 9,87E-07 |
| PAXIP1 | -7,954 | 2,41E-87 |
| ZNF737 | -7,747 | 1,08E-05 |
| CCN4 | -7,651 | 1,92E-152 |
| ST8SIA2 | -7,589 | 9,87E-13 |
| LINC01287 | -7,583 | 5,13E-06 |
| SYNDIG1 | -7,539 | 2,60E-26 |
| PPARGC1A | -7,492 | 3,44E-12 |
| CSMD1 | -7,382 | 7,40E-06 |
| FBXL21P | -7,361 | 3,61E-06 |
| DIP2C | -7,352 | 6,28E-104 |
| SH3BP1 | -7,339 | 8,17E-07 |
| GPX1P1 | -7,322 | 1,43E-04 |
| CLEC1A | -7,318 | 5,82E-06 |
| LIX1 | -7,286 | 5,00E-12 |
| TGM2 | -7,284 | 6,38E-07 |
| MX2 | -7,218 | 1,23E-06 |
| MIR3667HG | -7,202 | 1,87E-07 |
| ZNF730 | -7,174 | 3,29E-06 |
| CHI3L1 | -7,173 | 5,89E-04 |
| GRIP2 | -7,171 | 2,91E-09 |
| ENSG00000286617 | -7,117 | 2,81E-04 |
| DEFA9P | -7,048 | 4,78E-05 |
| MIR924HG | -7,027 | 1,00E-05 |
| FEZF2 | -6,999 | 1,02E-04 |
| ENSG00000284600 | -6,906 | 5,79E-04 |
| CAPN6 | -6,892 | 1,36E-04 |
| MAGEB1 | -6,863 | 3,84E-21 |
| MT1M | -6,801 | 1,04E-04 |
| IGHVIII-38-1 | -6,771 | 1,27E-04 |
| RAB26 | -6,714 | 2,06E-04 |
| LINC01238 | -6,677 | 3,88E-04 |
| SSX5 | -6,649 | 4,74E-04 |
| LINC00237 | -6,555 | 1,80E-04 |
| PROC | -6,545 | 1,47E-05 |
| CD200 | -6,543 | 7,09E-04 |
| PCDHGA11 | -6,525 | 5,82E-90 |
| LINC02387 | -6,523 | 4,30E-04 |
| COLEC12 | -6,519 | 3,45E-40 |
| RERG | -6,516 | 1,31E-04 |
| FIBIN | -6,505 | 2,12E-30 |
| PLD5 | -6,493 | 6,12E-05 |
| EMSLR | -6,479 | 4,27E-04 |
| ENSG00000256237 | -6,447 | 9,40E-04 |
| COPG2IT1 | -6,441 | 5,62E-03 |
| TMPRSS15 | -6,421 | 7,49E-06 |
| ADORA1 | -6,411 | 4,22E-16 |
| PDE9A | -6,323 | 6,14E-07 |
| CECR7 | -6,321 | 4,34E-04 |
| PAXIP1-DT | -6,287 | 4,60E-22 |
| LINC00355 | -6,287 | 3,27E-07 |
| NAA11 | -6,283 | 3,51E-04 |
| ENSG00000260073 | -6,244 | 2,22E-04 |
| EMILIN1 | -6,238 | 1,11E-10 |
| GPR39 | -6,230 | 4,99E-07 |
| DEFA8P | -6,199 | 4,36E-03 |
| LINC00670 | -6,163 | 1,23E-04 |
| PLEKHA6 | -6,159 | 1,59E-11 |
| CPPED1 | -6,115 | 6,69E-04 |
| NPBWR1 | -6,104 | 6,96E-04 |
| ARMC3 | -6,101 | 3,10E-04 |
| COL1A2 | -6,085 | 5,60E-67 |
| SORCS2 | -6,061 | 2,14E-05 |
| ADRA2A | -6,045 | 1,11E-11 |
| ZNF93 | -6,039 | 1,75E-08 |
| NFYAP1 | -6,034 | 4,09E-03 |
| COL22A1 | -6,018 | 8,33E-05 |
| MLPH | -6,016 | 1,53E-03 |
| IGHV3-38 | -6,012 | 6,18E-03 |
| ARHGAP36 | -5,994 | 7,93E-07 |
| ENSG00000268560 | -5,992 | 1,22E-03 |
| ECEL1 | -5,975 | 2,28E-07 |
| MXRA5Y | -5,946 | 1,80E-02 |
| LINC02416 | -5,944 | 2,37E-03 |
| CSPG4 | -5,932 | 3,94E-28 |
| GUCY1A1 | -5,926 | 4,91E-09 |
| C11orf16 | -5,921 | 2,65E-04 |
| ENSG00000286257 | -5,908 | 2,11E-04 |
| RBP2 | -5,907 | 1,74E-03 |
| ENSG00000273183 | -5,907 | 3,23E-04 |
| ENSG00000250038 | -5,820 | 5,96E-03 |
| TMEM130 | -5,816 | 1,19E-06 |
| ENSG00000258081 | -5,698 | 9,40E-03 |
| CLEC7A | -5,690 | 2,96E-02 |
| IL6-AS1 | -5,689 | 1,03E-02 |
| CDCP1 | -5,680 | 1,05E-02 |
| ADGRF1 | -5,680 | 4,96E-02 |
| NLGN4X | -5,645 | 1,08E-05 |
| NSUN5 | -5,638 | 2,74E-67 |
| MXRA5 | -5,632 | 6,40E-07 |
| TAGAP | -5,632 | 7,85E-17 |
| LINC01954 | -5,616 | 3,87E-02 |
| GPRC5A | -5,578 | 3,13E-09 |
| COL3A1 | -5,572 | 1,57E-09 |
| LINC02904 | -5,553 | 5,02E-03 |
| ENSG00000284610 | -5,508 | 1,35E-02 |
| CLCNKB | -5,460 | 1,35E-02 |
| ENSG00000276627 | -5,458 | 1,32E-02 |
| FLJ36000 | -5,458 | 1,09E-02 |
| CPA6 | -5,436 | 2,93E-02 |
| CSMD2 | -5,435 | 6,38E-04 |
| ENSG00000254194 | -5,432 | 2,55E-03 |
| ZAR1 | -5,428 | 1,55E-02 |
| LTF | -5,397 | 7,41E-03 |
| ENSG00000280011 | -5,389 | 3,43E-03 |
| CYTL1 | -5,386 | 2,55E-02 |
| NKAIN4 | -5,376 | 2,28E-03 |
| ENSG00000289096 | -5,374 | 6,21E-03 |
| IRAG1 | -5,366 | 4,71E-27 |
| PRDX2 | -5,356 | 1,63E-91 |
| OGN | -5,350 | 3,80E-03 |
| LRRN2 | -5,349 | 4,21E-03 |
| LY6G6C | -5,340 | 1,75E-02 |
| PARD6G | -5,332 | 1,33E-27 |
| HSPB1P1 | -5,319 | 2,58E-03 |
| OR7E104P | -5,286 | 2,89E-02 |
| HSPB1 | -5,272 | 1,18E-70 |
| EHBP1L1 | -5,270 | 1,64E-19 |
| TRAPPC13P1 | -5,270 | 2,23E-03 |
| PAGE2B | -5,267 | 4,62E-03 |
| KCNE4 | -5,243 | 5,02E-13 |
| LINC01237 | -5,227 | 4,82E-02 |
| TGFBI | -5,209 | 2,61E-07 |
| VSIG8 | -5,204 | 2,09E-02 |
| ITGA9 | -5,203 | 4,76E-04 |
| SLC6A9 | -5,180 | 9,74E-12 |
| GPR87 | -5,158 | 4,89E-03 |
| DRD2 | -5,155 | 4,97E-05 |
| ELN | -5,126 | 5,19E-04 |
| MEST | -5,087 | 2,21E-05 |
| TBX21 | -5,086 | 5,76E-03 |
| FNDC11 | -5,079 | 1,38E-02 |
| PROK1 | -5,077 | 3,28E-03 |
| EEF1AKMT1 | -5,028 | 3,91E-16 |
| CACNG4 | -5,009 | 1,03E-22 |
| CHST8 | -4,999 | 6,20E-03 |
| GJB6 | -4,991 | 1,07E-02 |
| NLRP4 | -4,988 | 4,23E-02 |
| GATA4 | -4,965 | 4,89E-02 |
| ENSG00000269729 | -4,922 | 2,56E-03 |
| LINC00840 | -4,901 | 1,29E-02 |
| FCRL6 | -4,896 | 6,03E-03 |
| GRID2 | -4,870 | 9,53E-05 |
| ENSG00000285722 | -4,848 | 5,38E-03 |
| PNMA8B | -4,824 | 5,07E-03 |
| MAP1LC3C | -4,802 | 2,49E-03 |
| WDFY4 | -4,793 | 4,30E-02 |
| MAOB | -4,786 | 2,74E-02 |
| ENSG00000258592 | -4,783 | 9,43E-03 |
| LGALS9 | -4,781 | 3,17E-02 |
| RPS6KA2 | -4,775 | 9,46E-06 |
| IGFN1 | -4,755 | 2,66E-18 |
| SSX6P | -4,745 | 5,89E-06 |
| ENSG00000247134 | -4,698 | 6,10E-08 |
| ENSG00000278000 | -4,682 | 8,53E-04 |
| PCED1B-AS1 | -4,665 | 1,35E-04 |
| CHRNA1 | -4,641 | 9,28E-03 |
| MIR325HG | -4,632 | 4,34E-07 |
| HAPLN3 | -4,627 | 1,29E-06 |
| ENSG00000248975 | -4,600 | 3,63E-05 |
| CNTN1 | -4,599 | 1,84E-08 |
| PARD6G-AS1 | -4,574 | 2,33E-04 |
| ENSG00000234692 | -4,554 | 3,80E-02 |
| LINC02320 | -4,552 | 2,12E-02 |
| GPR183 | -4,540 | 4,10E-21 |
| CTSZ | -4,527 | 1,22E-13 |
| CXCL13 | -4,522 | 1,52E-07 |
| ENSG00000286616 | -4,499 | 1,47E-06 |
| LINC02616 | -4,497 | 1,10E-03 |
| ENSG00000251511 | -4,469 | 2,37E-02 |
| FMO3 | -4,441 | 6,08E-03 |
| NPR3 | -4,433 | 6,34E-16 |
| LINC02643 | -4,430 | 3,33E-02 |
| ENSG00000287996 | -4,423 | 1,90E-02 |
| ARHGAP29 | -4,411 | 1,23E-04 |
| PTPRZ1 | -4,406 | 5,84E-06 |
| JAKMIP3 | -4,353 | 1,99E-08 |
| VAT1L | -4,343 | 2,76E-02 |
| ANGPTL4 | -4,339 | 5,20E-06 |
| ENSG00000260850 | -4,316 | 3,08E-02 |
| ENSG00000248837 | -4,309 | 1,48E-03 |
| LINC01483 | -4,303 | 2,30E-02 |
| TBX1 | -4,294 | 1,49E-02 |
| SCUBE1 | -4,292 | 7,10E-24 |
| CCND1 | -4,291 | 1,26E-22 |
| APCDD1L | -4,287 | 6,07E-04 |
| SEMA3C | -4,280 | 4,44E-05 |
| LINC02506 | -4,279 | 3,59E-02 |
| LMOD1 | -4,248 | 2,33E-08 |
| ZSCAN23 | -4,227 | 1,09E-08 |
| WFDC21P | -4,225 | 3,33E-04 |
| ARPIN | -4,219 | 4,85E-06 |
| SUSD2 | -4,218 | 1,67E-11 |
| PTPRE | -4,216 | 8,47E-17 |
| ATP1A2 | -4,215 | 8,51E-06 |
| ENSG00000225172 | -4,206 | 1,89E-02 |
| CCR3 | -4,202 | 1,46E-05 |
| SPOCK1 | -4,177 | 4,16E-25 |
| CEACAM1 | -4,176 | 3,90E-06 |
| LINC02154 | -4,172 | 7,76E-06 |
| C1QL2 | -4,165 | 2,50E-04 |
| GAGE2A | -4,160 | 4,78E-04 |
| MTMR11 | -4,139 | 2,44E-10 |
| CXCL12 | -4,123 | 4,27E-02 |
| GDA | -4,121 | 1,39E-03 |
| ENSG00000286523 | -4,114 | 4,63E-02 |
| MAP3K14-AS1 | -4,096 | 4,89E-02 |
| WNT7B | -4,095 | 1,32E-03 |
| ENSG00000223703 | -4,087 | 2,35E-03 |
| MDGA2 | -4,068 | 5,36E-22 |
| JAKMIP2-AS1 | -4,065 | 2,76E-02 |
| GPR50-AS1 | -4,053 | 6,01E-03 |
| RSPO3 | -4,052 | 6,92E-04 |
| NLRP11 | -4,026 | 2,62E-07 |
| ZBED2 | -4,024 | 1,42E-02 |
| ENSG00000272180 | -4,019 | 3,17E-02 |
| GDNF | -4,002 | 1,17E-19 |
| THBD | -3,995 | 1,41E-19 |
| FHDC1 | -3,971 | 1,43E-13 |
| LINC02082 | -3,964 | 2,12E-03 |
| FAM78B | -3,962 | 5,60E-35 |
| PKP1 | -3,961 | 4,78E-03 |
| S100A5 | -3,958 | 1,16E-04 |
| MAGEC1 | -3,954 | 3,35E-04 |
| ABI3BP | -3,954 | 5,37E-05 |
| SSX8P | -3,946 | 4,46E-02 |
| SNED1 | -3,938 | 1,63E-12 |
| COL26A1 | -3,930 | 5,47E-08 |
| TMEM63C | -3,891 | 3,76E-03 |
| IL6 | -3,868 | 2,25E-02 |
| SLC25A48 | -3,864 | 4,73E-06 |
| TBX4 | -3,859 | 9,63E-03 |
| DIRAS1 | -3,853 | 4,09E-04 |
| RAB7B | -3,851 | 8,81E-08 |
| ENSG00000258919 | -3,847 | 8,38E-03 |
| SLCO2A1 | -3,836 | 2,07E-12 |
| ABCA13 | -3,832 | 1,74E-08 |
| CCR1 | -3,821 | 3,72E-03 |
| ENSG00000229588 | -3,820 | 2,80E-02 |
| FOXN1 | -3,814 | 3,62E-02 |
| RASGRF2 | -3,810 | 3,26E-05 |
| CEMIP | -3,805 | 1,61E-02 |
| STK32B | -3,797 | 5,29E-08 |
| GGT8P | -3,775 | 4,14E-05 |
| RAB36 | -3,772 | 5,70E-13 |
| CHRM2 | -3,771 | 7,42E-28 |
| P2RX6 | -3,741 | 2,39E-14 |
| EPS15P1 | -3,718 | 2,38E-05 |
| ANKS1B | -3,712 | 7,10E-17 |
| ZP1 | -3,702 | 2,73E-02 |
| GALNT16 | -3,664 | 6,69E-17 |
| RPRM | -3,663 | 2,56E-19 |
| ENSG00000284616 | -3,661 | 3,45E-05 |
| SHE | -3,656 | 5,81E-03 |
| NES | -3,653 | 1,23E-03 |
| LINC02882 | -3,651 | 2,43E-02 |
| NGF | -3,651 | 2,64E-05 |
| KANK4 | -3,647 | 6,57E-03 |
| ACKR3 | -3,639 | 6,87E-12 |
| ENSG00000282024 | -3,612 | 2,02E-02 |
| CAPG | -3,610 | 1,70E-05 |
| CALHM5 | -3,610 | 8,69E-06 |
| ENSG00000253520 | -3,609 | 1,92E-03 |
| TMEM158 | -3,598 | 2,47E-07 |
| GFRA1 | -3,597 | 1,79E-03 |
| GPR50 | -3,590 | 1,35E-03 |
| LINC01173 | -3,590 | 4,60E-02 |
| IHH | -3,590 | 7,05E-11 |
| LRCOL1 | -3,585 | 1,06E-02 |
| MMP24 | -3,578 | 4,72E-07 |
| MYOM1 | -3,578 | 2,08E-46 |
| FAM27C | -3,577 | 8,71E-07 |
| ALDH1A1 | -3,570 | 1,69E-03 |
| SUSD4 | -3,569 | 2,96E-13 |
| GRASLND | -3,566 | 7,68E-03 |
| SGPP2 | -3,527 | 1,68E-11 |
| LINC02915 | -3,525 | 6,37E-03 |
| ENSG00000282980 | -3,499 | 2,45E-03 |
| GALNT5 | -3,494 | 5,65E-09 |
| CACNA1B | -3,462 | 5,70E-03 |
| CDH13 | -3,457 | 8,27E-04 |
| LINC00239 | -3,441 | 1,54E-05 |
| GZMB | -3,439 | 1,38E-03 |
| ZNF469 | -3,437 | 5,92E-04 |
| SPANXA1 | -3,437 | 4,26E-03 |
| CYP4F11 | -3,414 | 4,41E-02 |
| LINC02381 | -3,407 | 4,35E-15 |
| LINC02864 | -3,399 | 4,85E-02 |
| ICOSLG | -3,394 | 4,29E-04 |
| ENSG00000243144 | -3,387 | 4,27E-02 |
| KIF25 | -3,384 | 2,89E-02 |
| CACNB2 | -3,382 | 5,69E-05 |
| RAB42 | -3,376 | 2,87E-02 |
| MYO7A | -3,372 | 7,59E-07 |
| MMP17 | -3,364 | 5,00E-24 |
| CA3 | -3,360 | 3,77E-08 |
| MFAP2 | -3,360 | 2,31E-02 |
| GIPC3 | -3,358 | 4,36E-48 |
| GDNF-AS1 | -3,339 | 6,25E-06 |
| PLXNB3 | -3,337 | 3,88E-32 |
| FAXC | -3,330 | 1,98E-03 |
| SPATA4 | -3,313 | 6,38E-07 |
| SFTA3 | -3,297 | 1,85E-07 |
| MIR1915HG | -3,296 | 2,13E-13 |
| MPP7 | -3,295 | 2,33E-06 |
| VAC14-AS1 | -3,281 | 6,57E-03 |
| LINC01694 | -3,271 | 1,33E-03 |
| FMOD | -3,265 | 3,18E-02 |
| CAMKV | -3,263 | 2,09E-03 |
| ENSG00000287642 | -3,263 | 7,25E-04 |
| BHMT2 | -3,262 | 1,21E-02 |
| QPRT | -3,256 | 8,52E-04 |
| CLIC2 | -3,249 | 3,78E-06 |
| PADI1 | -3,247 | 2,74E-02 |
| TSPOAP1 | -3,233 | 6,62E-05 |
| PDGFB | -3,232 | 2,52E-05 |
| LINC01500 | -3,228 | 1,96E-05 |
| CALCRL | -3,215 | 3,78E-04 |
| RIPPLY3 | -3,208 | 1,26E-05 |
| ENSG00000248927 | -3,204 | 1,79E-02 |
| LINC02029 | -3,202 | 1,49E-02 |
| GREM1 | -3,201 | 4,82E-16 |
| ENSG00000269993 | -3,191 | 3,54E-02 |
| ENSG00000287354 | -3,190 | 3,40E-02 |
| LTBP2 | -3,190 | 8,41E-03 |
| APOBEC3G | -3,187 | 2,00E-03 |
| ENSG00000277117 | -3,186 | 6,23E-04 |
| SLC16A6 | -3,184 | 1,95E-03 |
| ENSG00000287011 | -3,183 | 1,64E-03 |
| SLC22A23 | -3,183 | 6,34E-54 |
| LIPE | -3,182 | 1,31E-10 |
| IL6R-AS1 | -3,173 | 5,07E-03 |
| CRACDL | -3,172 | 9,19E-03 |
| PRRX1 | -3,170 | 1,17E-17 |
| NKD2 | -3,155 | 4,36E-07 |
| SUGCT | -3,150 | 5,01E-08 |
| GLRA3 | -3,145 | 1,58E-02 |
| ENSG00000250644 | -3,143 | 1,35E-02 |
| TLE2 | -3,142 | 3,44E-12 |
| LCN12 | -3,137 | 4,78E-02 |
| KCNJ2 | -3,129 | 1,55E-19 |
| RBM24 | -3,128 | 1,34E-04 |
| ACVR1C | -3,125 | 2,53E-29 |
| IGSF1 | -3,125 | 1,32E-03 |
| DKK2 | -3,124 | 2,38E-02 |
| HTATIP2 | -3,120 | 3,45E-48 |
| SFRP2 | -3,117 | 1,37E-03 |
| MTUS2 | -3,112 | 4,19E-10 |
| FAM189A2 | -3,109 | 1,45E-15 |
| FCMR | -3,096 | 2,83E-04 |
| IL6R | -3,094 | 3,69E-10 |
| CEND1 | -3,086 | 1,69E-02 |
| ENSG00000284391 | -3,082 | 4,71E-02 |
| HMX3 | -3,081 | 6,69E-04 |
| HTR1D | -3,078 | 2,79E-05 |
| HMCN2 | -3,070 | 2,22E-04 |
| SSTR1 | -3,070 | 1,24E-02 |
| METTL27 | -3,058 | 2,77E-09 |
| ANXA3 | -3,057 | 3,19E-06 |
| LINC01615 | -3,057 | 3,53E-09 |
| NUAK2 | -3,051 | 1,79E-11 |
| GRIN3B | -3,046 | 2,05E-07 |
| RBP7 | -3,036 | 2,05E-05 |
| ENSG00000253282 | -3,034 | 7,26E-04 |
| TCEAL2 | -3,033 | 2,84E-02 |
| MEGF6 | -3,030 | 8,57E-15 |
| ENSG00000250378 | -3,029 | 6,86E-03 |
| IGFBPL1 | -3,023 | 9,33E-07 |
| CACNA1E | -3,013 | 2,58E-02 |
| SPDYE18 | -3,009 | 9,07E-05 |
| RBP1 | -2,976 | 2,26E-04 |
| HHIPL1 | -2,971 | 1,33E-06 |
| WNT16 | -2,950 | 1,36E-02 |
| THBS2 | -2,949 | 1,36E-20 |
| TMEM236 | -2,943 | 2,33E-02 |
| CRLF1 | -2,940 | 3,26E-05 |
| CRYZL2P | -2,935 | 2,11E-06 |
| ENSG00000285090 | -2,932 | 1,25E-02 |
| PAPLN | -2,923 | 2,17E-10 |
| BAALC | -2,899 | 1,45E-26 |
| ENSG00000288658 | -2,892 | 8,78E-17 |
| ENSG00000271749 | -2,884 | 1,19E-02 |
| APCDD1L-DT | -2,867 | 2,26E-15 |
| PRKD1 | -2,851 | 1,56E-21 |
| CBFA2T3 | -2,847 | 3,76E-02 |
| EFEMP1 | -2,843 | 6,77E-10 |
| KCNMB2-AS1 | -2,821 | 5,35E-03 |
| LINC02302 | -2,800 | 1,90E-02 |
| ZFP82 | -2,787 | 1,40E-14 |
| RGS16 | -2,787 | 1,80E-02 |
| ACSL5 | -2,786 | 1,07E-02 |
| LINC01881 | -2,785 | 1,73E-02 |
| SLN | -2,784 | 8,18E-03 |
| ELF3 | -2,781 | 3,91E-02 |
| LDB3 | -2,778 | 3,03E-16 |
| GSPT2 | -2,778 | 6,12E-04 |
| RASIP1 | -2,777 | 7,90E-07 |
| CT75 | -2,773 | 1,04E-24 |
| HLA-F | -2,770 | 5,49E-29 |
| PGAM2 | -2,756 | 4,24E-02 |
| P2RX6P | -2,750 | 1,94E-02 |
| COL24A1 | -2,746 | 7,07E-39 |
| HTR1A | -2,741 | 1,99E-02 |
| TENT5B | -2,726 | 1,31E-08 |
| OLFML2B | -2,722 | 7,92E-07 |
| NINJ2 | -2,720 | 1,93E-05 |
| FLVCR2 | -2,716 | 5,07E-07 |
| SPTSSB | -2,695 | 4,31E-02 |
| PLIN2 | -2,680 | 1,70E-04 |
| BEAN1 | -2,679 | 4,06E-06 |
| APELA | -2,673 | 3,74E-03 |
| ENSG00000266718 | -2,664 | 3,63E-04 |
| WNT9A | -2,657 | 1,67E-08 |
| RGCC | -2,654 | 2,29E-03 |
| ENSG00000238279 | -2,653 | 7,79E-03 |
| GGT4P | -2,653 | 3,78E-02 |
| MVP | -2,628 | 9,23E-15 |
| ENSG00000256029 | -2,625 | 3,75E-02 |
| MELTF | -2,624 | 4,13E-02 |
| NME4 | -2,623 | 7,05E-12 |
| FSCN1 | -2,619 | 3,18E-21 |
| NLRP1 | -2,616 | 1,49E-21 |
| SERPINE1 | -2,614 | 5,42E-13 |
| FOXS1 | -2,612 | 3,94E-02 |
| NEBL | -2,604 | 1,19E-06 |
| C1S | -2,599 | 5,78E-06 |
| BEGAIN | -2,598 | 1,02E-10 |
| CDH12 | -2,594 | 1,17E-17 |
| ENSG00000244137 | -2,584 | 4,68E-02 |
| ENTPD3 | -2,583 | 1,81E-05 |
| GREM1-AS1 | -2,578 | 1,53E-04 |
| FCGBP | -2,571 | 7,45E-03 |
| CSF1 | -2,567 | 5,81E-03 |
| ENSG00000253508 | -2,565 | 9,92E-03 |
| DDX53 | -2,562 | 2,85E-02 |
| SRPK3 | -2,562 | 4,64E-07 |
| ITGB4 | -2,560 | 7,66E-03 |
| AKR1B10 | -2,558 | 3,03E-02 |
| ATP2A3 | -2,557 | 4,84E-05 |
| ENSG00000228430 | -2,552 | 4,86E-02 |
| RTN4RL1 | -2,545 | 1,78E-10 |
| EHD2 | -2,542 | 1,58E-11 |
| LINC02511 | -2,542 | 3,02E-02 |
| NHLH1 | -2,538 | 2,33E-02 |
| LGALS3 | -2,535 | 1,01E-08 |
| ZNF680 | -2,532 | 2,24E-32 |
| RASGEF1C | -2,531 | 1,15E-04 |
| IFITM10 | -2,520 | 2,03E-05 |
| CAMK1 | -2,518 | 2,66E-02 |
| PITPNM3 | -2,515 | 1,95E-07 |
| MOXD1 | -2,513 | 1,16E-02 |
| TTC9 | -2,509 | 4,74E-02 |
| TINAGL1 | -2,489 | 1,46E-03 |
| CILP | -2,486 | 2,45E-04 |
| GGT5 | -2,475 | 1,37E-03 |
| INSRR | -2,473 | 2,03E-05 |
| MYOCD | -2,457 | 1,73E-08 |
| SOCS2 | -2,452 | 3,14E-10 |
| CFAP45 | -2,451 | 8,34E-06 |
| PRAME | -2,441 | 6,12E-27 |
| ENSG00000250645 | -2,440 | 3,30E-02 |
| SIPA1L2 | -2,437 | 5,49E-29 |
| SLC7A10 | -2,432 | 2,26E-05 |
| SULT1A1 | -2,429 | 1,90E-02 |
| BANCR | -2,415 | 3,79E-02 |
| NGEF | -2,408 | 1,87E-07 |
| ST8SIA6 | -2,405 | 1,27E-03 |
| GAS6-DT | -2,400 | 2,81E-03 |
| CPA4 | -2,398 | 3,76E-02 |
| ENSG00000231698 | -2,394 | 6,08E-03 |
| MAEL | -2,393 | 5,56E-05 |
| OMD | -2,385 | 1,09E-09 |
| CTF1 | -2,384 | 7,70E-04 |
| WNT1 | -2,376 | 2,71E-03 |
| SSTR2 | -2,376 | 1,23E-06 |
| RPP25 | -2,372 | 9,74E-12 |
| CRISPLD2 | -2,369 | 2,66E-08 |
| GUCY1A2 | -2,368 | 3,33E-07 |
| PPARG | -2,358 | 7,92E-05 |
| ALDH1L1 | -2,356 | 1,90E-11 |
| NLRX1 | -2,352 | 1,42E-26 |
| ENSG00000273796 | -2,348 | 3,99E-02 |
| SPTBN5 | -2,347 | 6,14E-04 |
| ASIC2 | -2,345 | 1,51E-03 |
| SVIL | -2,344 | 3,99E-05 |
| JPH2 | -2,336 | 2,02E-06 |
| CACNA1C | -2,331 | 1,32E-10 |
| ENSG00000286190 | -2,325 | 3,90E-08 |
| ADRA1D | -2,317 | 2,87E-09 |
| EPAS1 | -2,307 | 3,52E-09 |
| NRAP | -2,293 | 4,26E-15 |
| AP3B2 | -2,292 | 3,11E-05 |
| S100A4 | -2,289 | 3,43E-09 |
| RAMP1 | -2,284 | 5,32E-04 |
| WNT11 | -2,276 | 1,38E-09 |
| IP6K3 | -2,273 | 2,11E-04 |
| DHRS3 | -2,272 | 2,00E-02 |
| CAV2 | -2,263 | 1,50E-09 |
| MIR3681HG | -2,262 | 2,96E-02 |
| ELFN1 | -2,256 | 1,46E-09 |
| MAN1C1 | -2,255 | 4,60E-06 |
| MAP1B | -2,251 | 4,05E-04 |
| ENSG00000256732 | -2,250 | 1,24E-15 |
| TNNC1 | -2,249 | 4,95E-02 |
| FBLN1 | -2,245 | 5,21E-06 |
| SCARA5 | -2,243 | 2,56E-06 |
| MAGI2-AS3 | -2,238 | 1,36E-10 |
| LY6K | -2,233 | 2,01E-02 |
| C2orf66 | -2,232 | 2,39E-02 |
| LINC00705 | -2,230 | 2,31E-02 |
| SHISAL1 | -2,230 | 6,08E-17 |
| CYTIP | -2,229 | 4,26E-02 |
| FAP | -2,226 | 9,53E-03 |
| SLC38A4 | -2,226 | 1,01E-09 |
| PDGFRB | -2,219 | 4,98E-02 |
| FRMD5 | -2,210 | 1,10E-02 |
| VWF | -2,209 | 6,31E-05 |
| ENSG00000229425 | -2,206 | 2,26E-02 |
| S100A6 | -2,205 | 3,27E-20 |
| ENSG00000244560 | -2,204 | 1,11E-02 |
| CLCNKA | -2,202 | 1,04E-02 |
| ENSG00000225643 | -2,194 | 6,44E-03 |
| PSTPIP2 | -2,190 | 3,18E-04 |
| INHBB | -2,185 | 2,40E-03 |
| ENSG00000223652 | -2,184 | 6,59E-08 |
| LINC02798 | -2,184 | 5,79E-08 |
| TMEM240 | -2,179 | 2,63E-02 |
| PFKP | -2,175 | 3,90E-09 |
| HCN2 | -2,173 | 5,41E-07 |
| AOAH | -2,169 | 1,35E-02 |
| SERPINA9 | -2,166 | 3,63E-02 |
| HIC1 | -2,157 | 4,48E-02 |
| P3H2 | -2,156 | 1,82E-03 |
| ASB5 | -2,147 | 2,02E-06 |
| RASGEF1A | -2,141 | 1,95E-04 |
| FZD9 | -2,138 | 8,51E-06 |
| PCOTH | -2,138 | 1,78E-10 |
| ENSG00000283538 | -2,135 | 7,62E-03 |
| SLC26A11 | -2,131 | 2,30E-04 |
| RASD1 | -2,130 | 1,65E-03 |
| FOXQ1 | -2,125 | 2,41E-03 |
| DDIT4L | -2,124 | 1,97E-02 |
| PCSK9 | -2,122 | 3,60E-02 |
| IFITM1 | -2,121 | 2,51E-07 |
| SOCS2-AS1 | -2,116 | 5,20E-06 |
| GCNT4 | -2,110 | 8,77E-04 |
| STK31 | -2,109 | 1,02E-02 |
| ILDR2 | -2,108 | 2,98E-17 |
| FER1L6 | -2,107 | 4,53E-02 |
| LAMA3 | -2,105 | 1,18E-10 |
| ENSG00000232524 | -2,103 | 8,65E-03 |
| PLXNA2 | -2,101 | 5,50E-11 |
| AFAP1L2 | -2,097 | 1,23E-04 |
| ADRB2 | -2,096 | 1,82E-03 |
| MINDY4 | -2,085 | 3,68E-04 |
| IER3 | -2,072 | 4,44E-05 |
| ECM1 | -2,071 | 1,70E-04 |
| BDKRB2 | -2,069 | 8,24E-04 |
| RASSF4 | -2,069 | 1,27E-03 |
| HEPHL1 | -2,066 | 4,34E-02 |
| ULK4P3 | -2,065 | 8,21E-06 |
| SLC9A3R1 | -2,063 | 5,27E-17 |
| GALNT17 | -2,046 | 1,61E-08 |
| BMERB1 | -2,041 | 7,59E-25 |
| ADAMTS14 | -2,028 | 3,62E-02 |
| CD101 | -2,027 | 6,22E-03 |
| COLEC10 | -2,024 | 9,41E-04 |
| AJM1 | -2,023 | 5,64E-04 |
| PPFIA4 | -2,022 | 1,54E-09 |
| MYL3 | -2,021 | 1,45E-03 |
| PITPNC1 | -2,017 | 1,20E-10 |
| UMODL1-AS1 | -2,016 | 2,37E-02 |
| BDKRB1 | -2,014 | 3,70E-02 |
| HOXA13 | -2,009 | 5,09E-19 |
| ENSG00000289520 | -2,004 | 9,52E-07 |
| CD200R1L | -2,003 | 3,75E-02 |
| P4HA3 | -2,002 | 9,68E-03 |

**Supplementary Table 4. Significantly upregulated genes in Saos-2-ADAR2 compared to Saos2-E/A cells.**

| **Gene Name** | **Fold Change** | **Padj** |
| --- | --- | --- |
| COL4A5 | 10,957 | 2,16E-16 |
| NFIB | 10,202 | 3,25E-15 |
| BEND7 | 9,923 | 5,78E-14 |
| CSMD3 | 9,884 | 1,00E-17 |
| TAC3 | 9,474 | 3,97E-05 |
| C4orf19 | 9,303 | 7,28E-12 |
| GREB1L | 9,134 | 1,05E-11 |
| BCHE | 9,021 | 3,19E-63 |
| ASXL3 | 8,978 | 8,41E-10 |
| COL4A5 | 8,942 | 1,03E-45 |
| ELAVL2 | 8,922 | 3,66E-59 |
| ADAM23 | 8,744 | 2,18E-10 |
| DSP | 8,660 | 3,95E-20 |
| MARCHF1 | 8,556 | 4,30E-09 |
| PRRG4 | 8,507 | 7,74E-10 |
| UGT8 | 8,377 | 3,48E-19 |
| ERG | 8,364 | 1,54E-12 |
| HPN | 8,349 | 5,65E-09 |
| KCNK5 | 8,219 | 2,71E-09 |
| CNTN5 | 8,161 | 1,99E-08 |
| NPY1R | 8,036 | 1,29E-08 |
| CYYR1 | 7,970 | 1,86E-17 |
| ADGRL3 | 7,962 | 3,81E-11 |
| F11R | 7,921 | 4,83E-09 |
| PDE3B | 7,880 | 8,13E-11 |
| CERS4 | 7,879 | 1,02E-07 |
| TRPC4 | 7,872 | 2,46E-06 |
| THNSL2 | 7,744 | 5,02E-08 |
| CFHR3 | 7,734 | 1,26E-06 |
| FXYD5 | 7,619 | 2,48E-52 |
| PRSS16 | 7,573 | 5,69E-07 |
| HTR1F | 7,560 | 5,94E-21 |
| LINC01684 | 7,521 | 2,39E-10 |
| ESR1 | 7,509 | 4,92E-05 |
| ELOVL2 | 7,351 | 3,94E-28 |
| PTPRO | 7,334 | 4,57E-07 |
| TENM1 | 7,316 | 1,94E-103 |
| ENSG00000270977 | 7,268 | 4,68E-06 |
| HOOK1 | 7,262 | 9,90E-52 |
| LIN28B | 7,213 | 2,73E-05 |
| PGR | 7,191 | 3,72E-06 |
| PWWP3B | 7,187 | 8,78E-09 |
| PDZRN3 | 7,115 | 7,77E-06 |
| ENSG00000288765 | 7,099 | 4,27E-06 |
| JAM2 | 7,090 | 8,43E-30 |
| NOVA1 | 7,088 | 2,94E-06 |
| IGF2BP1 | 7,034 | 2,32E-84 |
| HAND2 | 7,019 | 4,23E-06 |
| ENSG00000289090 | 7,009 | 3,02E-08 |
| LINC02830 | 7,005 | 1,94E-05 |
| ADGRB3 | 6,999 | 1,33E-17 |
| ENSG00000225280 | 6,989 | 2,42E-06 |
| ANKRD18DP | 6,952 | 3,84E-05 |
| SULT1B1 | 6,944 | 4,17E-05 |
| EMB | 6,810 | 5,03E-58 |
| TMEM204 | 6,781 | 2,46E-04 |
| ENSG00000260947 | 6,776 | 9,86E-43 |
| RDM1 | 6,739 | 4,18E-06 |
| PCDH17 | 6,719 | 8,55E-22 |
| ENSG00000287063 | 6,707 | 3,11E-05 |
| ZBED9 | 6,690 | 2,25E-19 |
| ENSG00000219445 | 6,647 | 1,38E-04 |
| LINC01012 | 6,642 | 2,17E-05 |
| ABI3 | 6,584 | 6,65E-05 |
| PSG5 | 6,534 | 1,44E-03 |
| NAALAD2 | 6,519 | 3,14E-66 |
| HMSD | 6,511 | 3,12E-03 |
| ZNF521 | 6,502 | 2,26E-28 |
| CHST1 | 6,501 | 7,20E-06 |
| ENSG00000232599 | 6,474 | 7,40E-04 |
| LHX8 | 6,471 | 1,70E-34 |
| SLC7A3 | 6,402 | 1,11E-04 |
| FAM110B | 6,401 | 2,09E-07 |
| BEND4 | 6,371 | 2,51E-14 |
| MAP3K9-DT | 6,325 | 5,95E-05 |
| ZDBF2 | 6,317 | 6,94E-65 |
| ESRP1 | 6,272 | 5,55E-04 |
| ZNF391 | 6,217 | 8,39E-17 |
| SPOCK3 | 6,208 | 6,88E-05 |
| CPXCR1 | 6,199 | 9,07E-04 |
| LINC01788 | 6,195 | 1,33E-03 |
| ZNF804A | 6,178 | 2,90E-28 |
| MAP3K5 | 6,178 | 2,70E-14 |
| POF1B | 6,129 | 1,39E-03 |
| LINC02450 | 6,128 | 1,43E-03 |
| NPY5R | 6,076 | 1,65E-03 |
| SLAIN1 | 6,065 | 2,69E-14 |
| SLITRK3 | 6,064 | 1,51E-11 |
| NTRK2 | 6,058 | 2,81E-22 |
| SFRP1 | 6,054 | 1,66E-09 |
| GABRA2 | 6,003 | 1,56E-03 |
| PLAG1 | 5,974 | 5,86E-26 |
| CCDC170 | 5,951 | 2,36E-03 |
| TRBV26OR9-2 | 5,900 | 3,30E-08 |
| LRAT | 5,860 | 8,12E-03 |
| GATA6-AS1 | 5,815 | 2,37E-03 |
| NMU | 5,770 | 3,41E-23 |
| FUT1 | 5,707 | 9,37E-04 |
| ANK2 | 5,693 | 4,40E-63 |
| KRT81 | 5,683 | 1,40E-02 |
| FAM83B | 5,682 | 4,31E-03 |
| LINC02518 | 5,671 | 9,45E-03 |
| SCUBE3 | 5,667 | 1,20E-08 |
| FGD3 | 5,642 | 3,22E-17 |
| ABCC9 | 5,622 | 5,73E-04 |
| ENSG00000226043 | 5,618 | 8,78E-08 |
| ENSG00000231424 | 5,613 | 4,70E-03 |
| SYK | 5,607 | 9,37E-57 |
| PTGER3 | 5,607 | 5,40E-08 |
| NKX2-4 | 5,581 | 3,96E-12 |
| MSI1 | 5,576 | 3,79E-05 |
| FGG | 5,542 | 1,97E-02 |
| GPRIN3 | 5,542 | 4,89E-06 |
| CD74 | 5,536 | 1,51E-03 |
| TUBB4A | 5,501 | 6,28E-11 |
| DIO2-AS1 | 5,494 | 2,15E-05 |
| LRRC53 | 5,492 | 4,46E-03 |
| H3C6 | 5,488 | 9,06E-25 |
| LINC01807 | 5,460 | 1,81E-02 |
| PTENP1 | 5,457 | 2,87E-11 |
| EPHA7 | 5,443 | 9,39E-04 |
| NPY2R | 5,438 | 8,75E-03 |
| LGALS2 | 5,432 | 3,81E-03 |
| OCA2 | 5,419 | 1,35E-02 |
| SWAP70 | 5,400 | 9,93E-23 |
| ABCA8 | 5,370 | 2,11E-02 |
| HTR2A | 5,348 | 3,73E-02 |
| ARHGAP15 | 5,336 | 7,70E-04 |
| ENSG00000225606 | 5,330 | 3,40E-02 |
| ENSG00000253619 | 5,314 | 2,11E-04 |
| ZNF556 | 5,314 | 4,72E-03 |
| SCN3A | 5,283 | 4,08E-04 |
| LINC01305 | 5,263 | 3,85E-02 |
| PCDH11X | 5,242 | 1,10E-06 |
| GPR27 | 5,239 | 6,02E-03 |
| TBX22 | 5,237 | 1,84E-02 |
| OXGR1 | 5,234 | 1,75E-04 |
| ENSG00000256616 | 5,232 | 3,87E-03 |
| ENSG00000285838 | 5,207 | 4,60E-02 |
| PAK5 | 5,194 | 3,22E-04 |
| MPZL2 | 5,178 | 2,27E-02 |
| ENSG00000253894 | 5,143 | 9,22E-03 |
| EXTL1 | 5,137 | 1,40E-02 |
| LINC00383 | 5,122 | 9,97E-03 |
| LRRC37A4P | 5,102 | 1,81E-32 |
| EDA | 5,089 | 2,73E-20 |
| ADD2 | 5,078 | 2,17E-11 |
| PDE11A | 5,055 | 1,94E-11 |
| FREM2 | 5,050 | 1,66E-02 |
| ADAMTS19 | 5,038 | 9,99E-03 |
| ENSG00000283982 | 5,033 | 5,05E-03 |
| PGAP4 | 4,999 | 2,04E-29 |
| MAPK4 | 4,991 | 1,79E-05 |
| GMFG | 4,975 | 3,03E-10 |
| COL4A6 | 4,972 | 3,61E-12 |
| CCDC198 | 4,952 | 1,92E-16 |
| ENSG00000287382 | 4,942 | 3,29E-02 |
| COL19A1 | 4,921 | 2,73E-03 |
| FGD4 | 4,918 | 5,22E-04 |
| MARCHF4 | 4,906 | 2,38E-02 |
| LGI1 | 4,898 | 2,88E-02 |
| ABCG1 | 4,894 | 4,08E-03 |
| ADAMTS12 | 4,893 | 2,20E-07 |
| ZNF204P | 4,866 | 1,15E-11 |
| SNAP91 | 4,857 | 1,09E-15 |
| HOXC12 | 4,855 | 4,31E-02 |
| FAM107B | 4,849 | 3,96E-17 |
| WAKMAR2 | 4,838 | 1,24E-03 |
| BTC | 4,822 | 1,96E-05 |
| ENSG00000275139 | 4,818 | 1,62E-08 |
| PTENP1-AS | 4,805 | 4,80E-15 |
| ENSG00000236393 | 4,786 | 9,60E-03 |
| FAM162B | 4,781 | 1,25E-03 |
| FOXF1 | 4,779 | 2,85E-02 |
| FBLN5 | 4,765 | 1,69E-02 |
| MIR4432HG | 4,752 | 2,94E-03 |
| MRGPRF | 4,736 | 3,82E-02 |
| MPZL3 | 4,732 | 2,13E-06 |
| RGMA | 4,732 | 2,02E-09 |
| PDE6G | 4,725 | 1,06E-13 |
| GPC3 | 4,717 | 1,91E-18 |
| QRFPR | 4,710 | 2,08E-07 |
| CYP2J2 | 4,702 | 7,37E-09 |
| LRRC37A9P | 4,675 | 8,66E-04 |
| DOK5 | 4,668 | 2,73E-04 |
| KCND2 | 4,667 | 3,98E-03 |
| XKR5 | 4,664 | 1,15E-09 |
| ENSG00000232667 | 4,663 | 2,27E-04 |
| CLIC6 | 4,650 | 9,71E-26 |
| CA8 | 4,646 | 4,98E-03 |
| GLDC | 4,635 | 3,33E-02 |
| GATA6 | 4,617 | 3,62E-08 |
| ENPP7P2 | 4,585 | 4,37E-02 |
| SNCB | 4,576 | 3,92E-02 |
| MMRN2 | 4,570 | 9,32E-03 |
| TRPV4 | 4,567 | 4,18E-03 |
| ENSG00000230910 | 4,564 | 1,69E-04 |
| A2M | 4,549 | 1,43E-08 |
| PAK3 | 4,547 | 1,66E-09 |
| SNORA73 | 4,534 | 2,71E-02 |
| KREMEN2 | 4,526 | 2,41E-02 |
| KCNMB1 | 4,525 | 1,02E-02 |
| WNK2 | 4,523 | 3,34E-11 |
| LINC00987 | 4,520 | 1,35E-02 |
| KIF5C | 4,512 | 1,67E-31 |
| ENSG00000280385 | 4,509 | 1,86E-02 |
| LINC02152 | 4,492 | 1,62E-03 |
| IL18R1 | 4,455 | 1,85E-02 |
| C14orf180 | 4,433 | 3,98E-02 |
| ENSG00000280604 | 4,428 | 8,89E-07 |
| ANGPT4 | 4,423 | 3,87E-09 |
| SMIM10L2A | 4,383 | 2,91E-04 |
| BEX5 | 4,363 | 2,12E-02 |
| GALNT12 | 4,358 | 1,18E-10 |
| ZFPM2 | 4,356 | 1,26E-13 |
| APOL4 | 4,348 | 1,74E-08 |
| ALDH1A3-AS1 | 4,332 | 4,97E-11 |
| LAMB1 | 4,320 | 4,57E-32 |
| LRRK1 | 4,319 | 2,15E-23 |
| PTGIS | 4,310 | 1,33E-09 |
| ENSG00000287233 | 4,305 | 4,22E-02 |
| VIP | 4,293 | 4,94E-02 |
| EPB41L4A | 4,293 | 4,81E-02 |
| SEPTIN4-AS1 | 4,271 | 1,54E-05 |
| LPL | 4,247 | 2,84E-15 |
| NKX2-2 | 4,241 | 3,99E-02 |
| PNMA3 | 4,199 | 1,78E-02 |
| CTSV | 4,193 | 6,50E-29 |
| LMO1 | 4,189 | 2,74E-02 |
| CCSER1 | 4,183 | 9,37E-04 |
| TFAP2A-AS1 | 4,172 | 3,32E-03 |
| COBL | 4,153 | 3,24E-02 |
| BCL11B | 4,131 | 1,63E-02 |
| WFDC1 | 4,128 | 1,02E-09 |
| NLGN1 | 4,128 | 4,44E-05 |
| CERKL | 4,123 | 3,62E-09 |
| PCBP3 | 4,109 | 5,01E-11 |
| TCF15 | 4,106 | 8,50E-03 |
| NXPH2 | 4,104 | 1,05E-04 |
| SATB1-AS1 | 4,075 | 2,76E-04 |
| CYP27C1 | 4,050 | 1,34E-05 |
| F13A1 | 4,038 | 3,73E-04 |
| ENSG00000227733 | 4,038 | 4,39E-02 |
| ADCY2 | 4,037 | 2,22E-02 |
| RBM11 | 4,034 | 3,37E-31 |
| TEX15 | 4,019 | 4,24E-10 |
| DCAF12L2 | 4,018 | 1,41E-06 |
| MAP3K9 | 4,007 | 1,42E-20 |
| RAB3IP | 3,994 | 5,71E-36 |
| ID4 | 3,988 | 1,81E-04 |
| KCNJ8 | 3,979 | 2,15E-20 |
| SOST | 3,971 | 1,45E-03 |
| NAV2 | 3,960 | 9,93E-23 |
| HOXB13 | 3,944 | 8,16E-17 |
| ASB9 | 3,942 | 2,92E-02 |
| L1CAM | 3,940 | 3,61E-03 |
| SLITRK2 | 3,929 | 1,75E-10 |
| CRYBG2 | 3,928 | 3,34E-02 |
| LINC02232 | 3,921 | 9,28E-05 |
| ENSG00000230707 | 3,914 | 1,09E-02 |
| PRSS12 | 3,902 | 9,99E-21 |
| RPGRIP1 | 3,899 | 7,47E-03 |
| EML5 | 3,897 | 4,32E-28 |
| SIM2 | 3,876 | 4,91E-02 |
| HLA-DRA | 3,845 | 3,86E-06 |
| PENK | 3,832 | 2,43E-17 |
| ENSG00000235888 | 3,821 | 3,51E-08 |
| TGFA | 3,814 | 1,50E-11 |
| PRRT4 | 3,806 | 9,15E-04 |
| TMEM249 | 3,792 | 2,09E-02 |
| ZNF385B | 3,788 | 2,84E-15 |
| IL2RB | 3,777 | 3,93E-02 |
| CTTNBP2 | 3,749 | 2,13E-02 |
| GOLGA8IP | 3,747 | 3,85E-02 |
| HPCAL4 | 3,745 | 6,71E-07 |
| KCNT2 | 3,737 | 1,10E-11 |
| HLA-DRB1 | 3,736 | 1,33E-02 |
| COL15A1 | 3,734 | 1,03E-02 |
| SYT2 | 3,732 | 5,22E-03 |
| LINC01414 | 3,722 | 2,59E-02 |
| ABCC6P1 | 3,721 | 4,70E-02 |
| SP9 | 3,706 | 1,89E-03 |
| SLC25A18 | 3,699 | 3,15E-04 |
| ENSG00000225867 | 3,677 | 9,97E-04 |
| LMO3 | 3,675 | 4,13E-03 |
| SHANK3 | 3,666 | 2,88E-02 |
| RBP4 | 3,665 | 1,39E-11 |
| PENK-AS1 | 3,657 | 6,09E-05 |
| HOXB9 | 3,654 | 7,95E-06 |
| SHROOM4 | 3,645 | 5,48E-03 |
| CDH7 | 3,642 | 1,04E-06 |
| FGF10 | 3,606 | 4,44E-05 |
| ENSG00000273091 | 3,606 | 4,27E-02 |
| ADGRG6 | 3,604 | 5,31E-05 |
| KIAA1549L | 3,597 | 4,08E-07 |
| SH2D3C | 3,586 | 1,34E-04 |
| LRP2 | 3,582 | 4,64E-03 |
| RBPMS | 3,578 | 3,55E-12 |
| C2CD6 | 3,576 | 7,77E-06 |
| PRRT1 | 3,575 | 4,51E-02 |
| A2M-AS1 | 3,550 | 2,18E-10 |
| MAK | 3,542 | 5,51E-05 |
| LSP1 | 3,528 | 1,37E-10 |
| ENSG00000223563 | 3,514 | 4,37E-04 |
| AGGF1P1 | 3,509 | 3,38E-04 |
| ENSG00000231466 | 3,505 | 1,24E-03 |
| ENSG00000227482 | 3,503 | 1,24E-03 |
| STK33 | 3,502 | 3,26E-05 |
| OASL | 3,488 | 2,80E-08 |
| ENSG00000250049 | 3,473 | 1,23E-15 |
| SHISA3 | 3,457 | 5,09E-05 |
| SHANK2 | 3,447 | 2,48E-02 |
| AGBL1 | 3,444 | 3,06E-03 |
| ARFGEF3 | 3,430 | 7,70E-28 |
| ENSG00000287059 | 3,423 | 2,66E-02 |
| JAK3 | 3,420 | 3,12E-05 |
| ENSG00000234713 | 3,417 | 9,52E-04 |
| HEYL | 3,386 | 4,66E-06 |
| LRRK2 | 3,384 | 8,03E-10 |
| ENSG00000287175 | 3,383 | 3,10E-02 |
| ATP8A1 | 3,376 | 2,49E-14 |
| ARAP3 | 3,370 | 1,43E-08 |
| DCLK3 | 3,368 | 5,78E-03 |
| NKX6-1 | 3,366 | 2,08E-12 |
| NMNAT3 | 3,364 | 6,63E-08 |
| ANKFN1 | 3,358 | 2,67E-08 |
| ENSG00000235070 | 3,355 | 1,51E-02 |
| LINC01579 | 3,339 | 2,75E-15 |
| CAPN14 | 3,332 | 3,22E-03 |
| ANO3 | 3,328 | 2,25E-06 |
| LPAR3 | 3,316 | 2,79E-05 |
| ENSG00000237609 | 3,313 | 2,81E-02 |
| RYR3 | 3,311 | 2,37E-06 |
| S1PR3 | 3,310 | 7,65E-11 |
| TMTC1 | 3,289 | 1,19E-17 |
| ENSG00000267260 | 3,286 | 1,54E-04 |
| MBL2 | 3,283 | 1,32E-10 |
| ANO1 | 3,271 | 1,13E-04 |
| TMEFF2 | 3,267 | 1,27E-04 |
| MYOZ3 | 3,265 | 2,77E-04 |
| TOX2 | 3,264 | 2,17E-10 |
| ANKRD18CP | 3,263 | 3,17E-02 |
| ENSG00000266602 | 3,262 | 1,14E-04 |
| GSTA4 | 3,251 | 5,97E-60 |
| ENSG00000248538 | 3,248 | 1,93E-02 |
| BEX4 | 3,232 | 1,96E-06 |
| ENSG00000285407 | 3,225 | 2,82E-04 |
| OR8B10P | 3,224 | 1,10E-05 |
| ENSG00000288948 | 3,211 | 1,92E-03 |
| MACROH2A2 | 3,210 | 2,49E-12 |
| GRB14 | 3,204 | 2,90E-02 |
| ENSG00000269896 | 3,203 | 1,19E-02 |
| AGTR1 | 3,203 | 1,70E-04 |
| RAMP2 | 3,203 | 5,00E-03 |
| RYR1 | 3,193 | 1,91E-03 |
| CALB2 | 3,188 | 3,19E-06 |
| ENSG00000237594 | 3,182 | 6,74E-03 |
| PERP | 3,157 | 8,17E-08 |
| RYR2 | 3,155 | 3,35E-04 |
| TMEM100 | 3,152 | 1,43E-02 |
| ENSG00000254812 | 3,148 | 9,72E-03 |
| C12orf56 | 3,137 | 4,39E-02 |
| LINC02872 | 3,121 | 5,51E-03 |
| METTL24 | 3,117 | 4,95E-05 |
| ADAMTSL5 | 3,111 | 1,09E-02 |
| LINC01285 | 3,100 | 4,64E-02 |
| COL2A1 | 3,100 | 2,19E-05 |
| DIO2 | 3,099 | 7,75E-10 |
| PPP1R14C | 3,096 | 2,07E-03 |
| MC4R | 3,095 | 4,31E-03 |
| LYN | 3,082 | 4,24E-10 |
| HAS2 | 3,070 | 8,64E-10 |
| TIAM1 | 3,063 | 2,04E-30 |
| CCDC160 | 3,057 | 4,19E-02 |
| RNF180 | 3,054 | 1,31E-03 |
| MAP7 | 3,038 | 3,41E-02 |
| CTNNA3 | 3,037 | 5,16E-03 |
| ENSG00000279041 | 3,023 | 1,71E-02 |
| AMZ1 | 2,985 | 1,07E-05 |
| LNCOG | 2,976 | 4,32E-02 |
| HSPA12A | 2,960 | 7,66E-05 |
| ENSG00000231482 | 2,958 | 2,33E-04 |
| BGN | 2,947 | 2,38E-02 |
| MAL2 | 2,900 | 6,41E-06 |
| MIR1-1HG-AS1 | 2,897 | 1,19E-02 |
| EDNRA | 2,896 | 2,14E-02 |
| JAM3 | 2,892 | 4,73E-05 |
| DPEP1 | 2,883 | 3,03E-03 |
| BEX1 | 2,876 | 2,14E-04 |
| USP43 | 2,873 | 4,44E-05 |
| GALNT6 | 2,866 | 1,62E-07 |
| ENSG00000239219 | 2,864 | 1,15E-02 |
| GLUD2 | 2,852 | 7,00E-10 |
| ENSG00000288885 | 2,848 | 3,51E-03 |
| PSKH2 | 2,845 | 3,36E-02 |
| KCNMB4 | 2,835 | 1,30E-19 |
| FAM181B | 2,831 | 6,01E-03 |
| SLC8A1-AS1 | 2,821 | 2,73E-04 |
| SCN2A | 2,819 | 7,07E-05 |
| ANKRD20A11P | 2,818 | 2,12E-02 |
| ENSG00000261211 | 2,791 | 2,12E-02 |
| ENSG00000285517 | 2,791 | 1,56E-05 |
| LINC02542 | 2,789 | 1,94E-03 |
| NUPR1 | 2,780 | 4,84E-07 |
| TAMALIN | 2,776 | 1,51E-11 |
| MYL4 | 2,774 | 8,25E-04 |
| TSPAN33 | 2,773 | 2,11E-06 |
| SPARCL1 | 2,770 | 3,92E-05 |
| MAGEC2 | 2,768 | 1,87E-05 |
| PKIB | 2,764 | 7,56E-04 |
| PCDH10-DT | 2,763 | 4,33E-02 |
| AGGF1P2 | 2,754 | 2,53E-03 |
| GUSBP5 | 2,754 | 1,26E-03 |
| ENSG00000261120 | 2,753 | 4,91E-02 |
| KCNMB2 | 2,749 | 1,45E-02 |
| ICAM2 | 2,745 | 6,64E-04 |
| ENSG00000276170 | 2,739 | 2,39E-04 |
| DCHS2 | 2,736 | 1,26E-11 |
| SCIN | 2,736 | 3,68E-14 |
| GNGT2 | 2,730 | 3,74E-02 |
| ARHGAP24 | 2,704 | 2,94E-11 |
| ARAP2 | 2,704 | 2,48E-08 |
| RNF150 | 2,692 | 4,99E-07 |
| RIPOR3 | 2,686 | 6,56E-08 |
| ASTN1 | 2,678 | 2,35E-03 |
| NEGR1 | 2,674 | 2,71E-12 |
| CLSTN2 | 2,673 | 4,25E-05 |
| NFATC1 | 2,671 | 1,75E-10 |
| ENSG00000272991 | 2,660 | 2,77E-02 |
| SHC2 | 2,659 | 1,15E-05 |
| LGR5 | 2,656 | 1,95E-03 |
| ZNF385D | 2,654 | 6,98E-03 |
| OR8B9P | 2,647 | 8,13E-08 |
| COL4A4 | 2,643 | 1,54E-02 |
| FSD1 | 2,643 | 7,90E-04 |
| CHL1 | 2,642 | 2,73E-02 |
| PRL | 2,642 | 2,80E-03 |
| TRIM14 | 2,642 | 9,30E-08 |
| ENSG00000249001 | 2,633 | 1,06E-05 |
| ENSG00000289612 | 2,630 | 5,41E-04 |
| ENSG00000289591 | 2,630 | 3,64E-08 |
| TENT5C | 2,619 | 1,67E-07 |
| CGB5 | 2,615 | 1,07E-02 |
| C17orf113 | 2,612 | 4,74E-04 |
| IGFBP2 | 2,604 | 1,55E-07 |
| CD36 | 2,604 | 3,44E-13 |
| ANGPTL1 | 2,598 | 1,44E-02 |
| PLXNA4 | 2,597 | 2,12E-02 |
| SCN1B | 2,586 | 3,76E-14 |
| ENSG00000233817 | 2,582 | 4,94E-02 |
| BEX2 | 2,571 | 6,22E-07 |
| IFI44L | 2,561 | 5,09E-16 |
| ZBTB16 | 2,550 | 3,33E-02 |
| TRPM6 | 2,543 | 1,86E-04 |
| CCM2L | 2,540 | 8,81E-03 |
| LYPD6B | 2,539 | 2,72E-09 |
| ENSG00000203279 | 2,512 | 1,19E-08 |
| MTARC1 | 2,502 | 1,82E-03 |
| PTPRN | 2,489 | 1,41E-10 |
| DPY19L2P4 | 2,487 | 7,14E-04 |
| RND2 | 2,478 | 4,05E-02 |
| LRRC63 | 2,475 | 1,43E-02 |
| RERGL | 2,474 | 4,29E-02 |
| IL21R | 2,466 | 4,72E-03 |
| MYO3B | 2,462 | 1,33E-02 |
| RNF144B | 2,458 | 3,68E-09 |
| PRDM13 | 2,455 | 8,76E-09 |
| PLBD1 | 2,446 | 1,18E-10 |
| ENSG00000287038 | 2,446 | 3,57E-06 |
| TMEM150C | 2,445 | 3,80E-05 |
| ARHGAP20 | 2,442 | 4,93E-04 |
| NYNRIN | 2,442 | 9,36E-04 |
| ENSG00000272425 | 2,436 | 3,61E-05 |
| ABCB1 | 2,429 | 2,53E-03 |
| RFPL3S | 2,427 | 3,79E-02 |
| MEGF10 | 2,427 | 4,39E-02 |
| ZNF618 | 2,417 | 3,18E-27 |
| SPNS3 | 2,411 | 2,58E-03 |
| ENSG00000229116 | 2,409 | 1,94E-02 |
| ENSG00000225806 | 2,408 | 1,84E-03 |
| ENSG00000198580 | 2,401 | 1,96E-02 |
| LYPD3 | 2,399 | 1,22E-02 |
| HMGCLL1 | 2,396 | 4,04E-02 |
| GBP4 | 2,393 | 1,06E-04 |
| TFAP2C | 2,371 | 4,07E-03 |
| CCDC88C | 2,371 | 2,29E-08 |
| ACSS1 | 2,370 | 7,25E-08 |
| DCX | 2,368 | 1,74E-08 |
| CHGA | 2,367 | 1,33E-02 |
| TP63 | 2,366 | 7,02E-04 |
| ZNF883 | 2,360 | 1,22E-05 |
| EFNB3 | 2,353 | 1,03E-05 |
| LINC00639 | 2,349 | 2,95E-02 |
| AMH | 2,347 | 6,95E-04 |
| ENSG00000265702 | 2,345 | 3,21E-03 |
| BCL11A | 2,339 | 3,73E-07 |
| KLC3 | 2,325 | 2,46E-09 |
| HTRA3 | 2,325 | 3,73E-02 |
| TRMT9B | 2,321 | 5,40E-06 |
| PNMA6A | 2,316 | 3,59E-04 |
| ENSG00000261572 | 2,296 | 2,68E-02 |
| PALD1 | 2,295 | 4,55E-12 |
| BMP7 | 2,293 | 7,04E-03 |
| UBASH3B | 2,292 | 8,85E-08 |
| AMPH | 2,292 | 1,70E-09 |
| PTCHD4 | 2,290 | 3,76E-02 |
| CIITA | 2,274 | 1,07E-02 |
| ENSG00000230606 | 2,272 | 4,80E-03 |
| PLCG2 | 2,270 | 7,18E-09 |
| ADAM11 | 2,264 | 4,11E-05 |
| RHOV | 2,261 | 9,60E-03 |
| ZNF853 | 2,256 | 2,29E-11 |
| S1PR1 | 2,256 | 1,47E-05 |
| ADCY5 | 2,250 | 8,31E-05 |
| RGS9BP | 2,246 | 4,72E-06 |
| PLBD1-AS1 | 2,244 | 2,07E-03 |
| RORB | 2,234 | 1,58E-09 |
| C1orf115 | 2,220 | 3,12E-05 |
| MB | 2,218 | 1,75E-04 |
| ADPRH | 2,201 | 9,44E-04 |
| ENSG00000287097 | 2,192 | 3,44E-03 |
| NUDT10 | 2,184 | 1,02E-03 |
| ENSG00000254602 | 2,179 | 1,12E-02 |
| FCHO1 | 2,179 | 1,96E-03 |
| GSC | 2,173 | 1,02E-02 |
| GYPA | 2,173 | 1,09E-02 |
| ENSG00000261815 | 2,168 | 4,70E-02 |
| ITGBL1 | 2,140 | 1,20E-17 |
| DHRS2 | 2,134 | 3,59E-02 |
| ROBO3 | 2,130 | 1,96E-04 |
| PRR5 | 2,127 | 8,73E-11 |
| LONRF2 | 2,114 | 1,35E-02 |
| TMEM121 | 2,114 | 6,94E-04 |
| INPP5D | 2,114 | 3,42E-02 |
| LINC01429 | 2,107 | 3,34E-02 |
| CBLN2 | 2,105 | 1,90E-03 |
| ENSG00000259807 | 2,104 | 2,54E-02 |
| UNC5C | 2,102 | 2,58E-05 |
| SPTBN4 | 2,099 | 3,53E-03 |
| NCAM2 | 2,095 | 3,05E-19 |
| PPP1R1C | 2,095 | 4,26E-02 |
| MATN4 | 2,095 | 1,21E-02 |
| ENSG00000268926 | 2,068 | 2,06E-02 |
| FAM53A | 2,065 | 2,66E-03 |
| LINC01036 | 2,062 | 1,01E-03 |
| ENSG00000285783 | 2,057 | 3,00E-03 |
| ARHGAP27 | 2,050 | 2,96E-02 |
| GNG7 | 2,045 | 1,39E-04 |
| CLGN | 2,040 | 7,46E-06 |
| VGLL3 | 2,038 | 5,64E-18 |
| NTNG1 | 2,027 | 1,01E-08 |
| LINC02525 | 2,027 | 1,07E-02 |
| ENSG00000231829 | 2,023 | 2,24E-04 |
| HAS2-AS1 | 2,014 | 9,92E-05 |
| ITGA2 | 2,010 | 8,67E-06 |
| KCNK6 | 2,009 | 7,72E-05 |
| MAB21L1 | 2,004 | 1,10E-06 |

**Supplementary Table 5. Significant Biological Process Term associated with transcripts found to be downregulated between Saos-2-ADAR2 and Saos2-E/A cells.**

| **GO Biological Process** | **p value** |
| --- | --- |
| hepatic immune response (GO:0002384) | 9.84E-04 |
| apelin receptor signaling pathway (GO:0060183) | 9.84E-04 |
| regulation of synaptic transmission, glycinergic (GO:0060092) | 9.84E-04 |
| trans-synaptic signaling by nitric oxide, modulating synaptic transmission (GO:0099555) | 9.84E-04 |
| trans-synaptic signaling by soluble gas, modulating synaptic transmission (GO:0099554) | 9.84E-04 |
| trans-synaptic signaling by nitric oxide (GO:0099548) | 9.84E-04 |
| trans-synaptic signaling by soluble gas (GO:0099543) | 9.84E-04 |
| negative regulation of intracellular cholesterol transport (GO:0032384) | 9.84E-04 |
| negative regulation of intracellular sterol transport (GO:0032381) | 9.84E-04 |
| negative regulation of intracellular lipid transport (GO:0032378) | 9.84E-04 |
| negative regulation of receptor-mediated endocytosis involved in cholesterol transport (GO:1905601) | 9.84E-04 |
| synaptic signaling by nitric oxide (GO:0099163) | 9.84E-04 |
| regulation of peptidyl-tyrosine autophosphorylation (GO:1900084) | 9.84E-04 |
| positive regulation of G protein-coupled receptor internalization (GO:1904022) | 9.84E-04 |
| pulmonary myocardium development (GO:0003350) | 9.84E-04 |
| positive regulation of dopamine secretion (GO:0033603) | 5.74E-04 |
| regulation of serotonin secretion (GO:0014062) | 9.80E-04 |
| positive regulation of odontoblast differentiation (GO:1901331) | 9.80E-04 |
| regulation of bone mineralization involved in bone maturation (GO:1900157) | 9.80E-04 |
| negative regulation of smooth muscle contraction (GO:0045986) | 3.89E-04 |
| extracellular matrix assembly (GO:0085029) | 2.22E-07 |
| membrane depolarization during cardiac muscle cell action potential (GO:0086012) | 1.36E-04 |
| negative regulation of muscle contraction (GO:0045932) | 3.39E-05 |
| cardiac myofibril assembly (GO:0055003) | 1.83E-04 |
| negative regulation of platelet activation (GO:0010544) | 1.83E-04 |
| regulation of presynaptic cytosolic calcium ion concentration (GO:0099509) | 9.96E-04 |
| regulation of extracellular matrix disassembly (GO:0010715) | 9.96E-04 |
| negative regulation of calcium ion transmembrane transporter activity (GO:1901020) | 7.76E-05 |
| positive regulation of extracellular matrix organization (GO:1903055) | 3.15E-05 |
| regulation of renal system process (GO:0098801) | 5.07E-04 |
| membrane depolarization during action potential (GO:0086010) | 5.07E-04 |
| regulation of smooth muscle contraction (GO:0006940) | 1.60E-07 |
| negative regulation of chondrocyte differentiation (GO:0032331) | 7.77E-04 |
| negative regulation of cardiac muscle cell apoptotic process (GO:0010667) | 7.77E-04 |
| forebrain regionalization (GO:0021871) | 7.77E-04 |
| basement membrane organization (GO:0071711) | 2.40E-04 |
| regulation of cardiac muscle cell contraction (GO:0086004) | 7.47E-05 |
| monocyte chemotaxis (GO:0002548) | 9.46E-04 |
| regulation of actin filament-based movement (GO:1903115) | 2.85E-05 |
| negative regulation of intrinsic apoptotic signaling pathway in response to DNA damage (GO:1902230) | 2.92E-04 |
| regulation of dopamine secretion (GO:0014059) | 9.46E-04 |
| positive chemotaxis (GO:0050918) | 2.86E-06 |
| regulation of cardiocyte differentiation (GO:1905207) | 9.46E-04 |
| negative regulation of cartilage development (GO:0061037) | 2.92E-04 |
| myoblast fusion (GO:0007520) | 3.52E-04 |
| positive regulation of receptor internalization (GO:0002092) | 1.14E-03 |
| negative regulation of smooth muscle cell proliferation (GO:0048662) | 1.87E-05 |
| vasodilation (GO:0042311) | 1.87E-05 |
| negative regulation of ion transmembrane transporter activity (GO:0032413) | 7.08E-06 |
| vasculogenesis (GO:0001570) | 3.29E-07 |
| regulation of extracellular matrix organization (GO:1903053) | 3.74E-06 |
| positive regulation of release of sequestered calcium ion into cytosol (GO:0051281) | 3.01E-04 |
| regulation of cardiac muscle cell apoptotic process (GO:0010665) | 8.15E-04 |
| collagen fibril organization (GO:0030199) | 1.84E-05 |
| regulation of collagen metabolic process (GO:0010712) | 9.47E-04 |
| negative regulation of transporter activity (GO:0032410) | 2.14E-05 |
| regulation of blood coagulation (GO:0030193) | 9.34E-06 |
| regulation of intrinsic apoptotic signaling pathway in response to DNA damage (GO:1902229) | 1.10E-03 |
| myofibril assembly (GO:0030239) | 1.08E-05 |
| negative regulation of blood coagulation (GO:0030195) | 4.67E-04 |
| regulation of hemostasis (GO:1900046) | 1.24E-05 |
| cell-cell fusion (GO:0140253) | 1.26E-03 |
| syncytium formation by plasma membrane fusion (GO:0000768) | 1.26E-03 |
| fear response (GO:0042596) | 1.26E-03 |
| regulation of striated muscle cell apoptotic process (GO:0010662) | 1.26E-03 |
| regulation of muscle contraction (GO:0006937) | 5.33E-11 |
| striated muscle cell development (GO:0055002) | 1.42E-05 |
| smooth muscle contraction (GO:0006939) | 8.65E-05 |
| negative regulation of hemostasis (GO:1900047) | 5.36E-04 |
| regulation of platelet activation (GO:0010543) | 2.30E-04 |
| regulation of coagulation (GO:0050818) | 1.63E-05 |
| regulation of cardiac muscle contraction (GO:0055117) | 1.63E-05 |
| regulation of vasoconstriction (GO:0019229) | 4.28E-05 |
| regulation of striated muscle contraction (GO:0006942) | 1.54E-06 |
| excitatory postsynaptic potential (GO:0060079) | 4.88E-05 |
| regulation of smooth muscle cell proliferation (GO:0048660) | 1.08E-08 |
| negative regulation of coagulation (GO:0050819) | 6.99E-04 |
| positive regulation of wound healing (GO:0090303) | 1.28E-04 |
| positive regulation of osteoblast differentiation (GO:0045669) | 5.55E-05 |
| negative regulation of blood pressure (GO:0045776) | 7.94E-04 |
| G protein-coupled receptor signaling pathway, coupled to cyclic nucleotide second messenger (GO:0007187) | 3.39E-04 |
| regulation of wound healing (GO:0061041) | 1.34E-07 |
| positive regulation of calcium ion transport (GO:0051928) | 6.97E-07 |
| regulation of cytosolic calcium ion concentration (GO:0051480) | 4.33E-04 |
| chemical synaptic transmission, postsynaptic (GO:0099565) | 1.02E-04 |
| regulation of release of sequestered calcium ion into cytosol (GO:0051279) | 4.96E-05 |
| artery morphogenesis (GO:0048844) | 2.64E-04 |
| negative regulation of wound healing (GO:0061045) | 2.64E-04 |
| positive regulation of endothelial cell proliferation (GO:0001938) | 3.03E-05 |
| regulation of epidermal growth factor receptor signaling pathway (GO:0042058) | 6.85E-04 |
| cellular anatomical entity morphogenesis (GO:0032989) | 1.93E-06 |
| cellular component assembly involved in morphogenesis (GO:0010927) | 1.93E-06 |
| blood vessel diameter maintenance (GO:0097746) | 2.27E-07 |
| regulation of tube diameter (GO:0035296) | 2.27E-07 |
| extracellular matrix organization (GO:0030198) | 2.03E-12 |
| collagen metabolic process (GO:0032963) | 7.63E-04 |
| extracellular structure organization (GO:0043062) | 2.26E-12 |
| regulation of tube size (GO:0035150) | 2.52E-07 |
| external encapsulating structure organization (GO:0045229) | 2.51E-12 |
| regulation of blood circulation (GO:1903522) | 6.66E-11 |
| positive regulation of calcium ion transmembrane transport (GO:1904427) | 2.19E-04 |
| canonical Wnt signaling pathway (GO:0060070) | 2.78E-05 |
| regulation of muscle system process (GO:0090257) | 8.52E-10 |
| cardiac muscle cell differentiation (GO:0055007) | 1.17E-04 |
| positive regulation of response to wounding (GO:1903036) | 5.01E-04 |
| receptor internalization (GO:0031623) | 5.01E-04 |
| regulation of calcium ion transmembrane transporter activity (GO:1901019) | 1.04E-03 |
| positive regulation of chemotaxis (GO:0050921) | 2.37E-06 |
| animal organ regeneration (GO:0031100) | 1.15E-03 |
| regulation of response to wounding (GO:1903034) | 3.41E-07 |
| chondrocyte differentiation (GO:0002062) | 2.96E-04 |
| positive regulation of smooth muscle cell proliferation (GO:0048661) | 2.96E-04 |
| positive regulation of leukocyte chemotaxis (GO:0002690) | 1.58E-04 |
| positive regulation of monoatomic ion transport (GO:0043270) | 3.21E-08 |
| regulation of heart contraction (GO:0008016) | 6.53E-08 |
| morphogenesis of a branching epithelium (GO:0061138) | 1.11E-06 |
| negative regulation of monoatomic ion transmembrane transport (GO:0034766) | 3.94E-04 |
| negative regulation of cation transmembrane transport (GO:1904063) | 8.13E-04 |
| morphogenesis of a branching structure (GO:0001763) | 7.23E-07 |
| blood vessel morphogenesis (GO:0048514) | 6.94E-15 |
| negative regulation of monoatomic ion transport (GO:0043271) | 6.61E-05 |
| actomyosin structure organization (GO:0031032) | 3.55E-05 |
| regulation of endothelial cell proliferation (GO:0001936) | 1.91E-05 |
| striated muscle cell differentiation (GO:0051146) | 3.62E-08 |
| regulation of postsynaptic membrane potential (GO:0060078) | 1.35E-04 |
| heart contraction (GO:0060047) | 2.76E-04 |
| branching morphogenesis of an epithelial tube (GO:0048754) | 2.49E-05 |
| negative regulation of response to wounding (GO:1903035) | 5.66E-04 |
| cardiac muscle contraction (GO:0060048) | 1.16E-03 |
| positive regulation of vasculature development (GO:1904018) | 5.03E-06 |
| blood circulation (GO:0008015) | 1.30E-12 |
| fatty acid transport (GO:0015908) | 7.32E-04 |
| heart process (GO:0003015) | 2.27E-04 |
| muscle contraction (GO:0006936) | 1.21E-07 |
| negative regulation of intrinsic apoptotic signaling pathway (GO:2001243) | 4.61E-04 |
| positive regulation of angiogenesis (GO:0045766) | 1.54E-05 |
| regulation of blood pressure (GO:0008217) | 5.70E-06 |
| blood vessel development (GO:0001568) | 2.03E-14 |
| angiogenesis (GO:0001525) | 9.19E-10 |
| muscle cell differentiation (GO:0042692) | 2.70E-08 |
| regulation of signaling receptor activity (GO:0010469) | 1.01E-03 |
| regulation of calcium ion transmembrane transport (GO:1903169) | 2.11E-05 |
| negative regulation of lipid metabolic process (GO:0045833) | 1.10E-03 |
| tissue remodeling (GO:0048771) | 6.34E-04 |
| vasculature development (GO:0001944) | 2.75E-14 |
| striated muscle contraction (GO:0006941) | 3.97E-04 |
| regulation of leukocyte chemotaxis (GO:0002688) | 3.97E-04 |
| positive regulation of leukocyte migration (GO:0002687) | 7.81E-05 |
| regeneration (GO:0031099) | 4.91E-05 |
| regulation of system process (GO:0044057) | 7.52E-14 |
| regulation of calcium ion transport (GO:0051924) | 7.80E-07 |
| regulation of vasculature development (GO:1901342) | 5.77E-08 |
| regulation of chemotaxis (GO:0050920) | 6.65E-06 |
| circulatory system process (GO:0003013) | 1.96E-12 |
| positive regulation of DNA-binding transcription factor activity (GO:0051091) | 4.51E-06 |
| regulation of leukocyte migration (GO:0002685) | 2.84E-06 |
| regulation of angiogenesis (GO:0045765) | 1.32E-07 |
| negative regulation of secretion by cell (GO:1903531) | 1.81E-04 |
| muscle system process (GO:0003012) | 1.42E-07 |
| positive regulation of ERK1 and ERK2 cascade (GO:0070374) | 2.43E-05 |
| negative regulation of cell adhesion (GO:0007162) | 1.64E-07 |
| negative regulation of cell motility (GO:2000146) | 1.19E-07 |
| negative regulation of cell activation (GO:0050866) | 1.10E-05 |
| cardiac chamber morphogenesis (GO:0003206) | 6.18E-04 |
| positive regulation of monoatomic ion transmembrane transport (GO:0034767) | 2.41E-04 |
| cell chemotaxis (GO:0060326) | 1.27E-05 |
| regulation of mononuclear cell migration (GO:0071675) | 4.14E-04 |
| cell fate commitment (GO:0045165) | 1.85E-06 |
| locomotion (GO:0040011) | 1.53E-08 |
| negative regulation of cell migration (GO:0030336) | 4.91E-07 |
| regulation of osteoblast differentiation (GO:0045667) | 7.63E-04 |
| regulation of metal ion transport (GO:0010959) | 1.26E-08 |
| cartilage development (GO:0051216) | 1.16E-04 |
| tube morphogenesis (GO:0035239) | 1.61E-14 |
| negative regulation of cell-cell adhesion (GO:0022408) | 4.55E-05 |
| regulation of sequestering of calcium ion (GO:0051282) | 8.75E-04 |
| connective tissue development (GO:0061448) | 1.37E-05 |
| chemotaxis (GO:0006935) | 1.30E-07 |
| taxis (GO:0042330) | 1.48E-07 |
| negative regulation of locomotion (GO:0040013) | 4.02E-07 |
| vascular process in circulatory system (GO:0003018) | 4.69E-06 |
| regulation of monoatomic ion transport (GO:0043269) | 3.45E-09 |
| positive regulation of cation transmembrane transport (GO:1904064) | 1.14E-03 |
| muscle cell development (GO:0055001) | 3.34E-04 |
| regulation of body fluid levels (GO:0050878) | 2.98E-07 |
| muscle tissue development (GO:0060537) | 6.07E-07 |
| positive regulation of cytosolic calcium ion concentration (GO:0007204) | 6.44E-04 |
| striated muscle tissue development (GO:0014706) | 1.89E-04 |
| negative regulation of secretion (GO:0051048) | 6.84E-04 |
| muscle structure development (GO:0061061) | 2.17E-09 |
| positive regulation of epithelial cell proliferation (GO:0050679) | 2.13E-04 |
| monocarboxylic acid transport (GO:0015718) | 1.23E-03 |
| cardiac chamber development (GO:0003205) | 8.68E-04 |
| cardiac muscle tissue development (GO:0048738) | 4.05E-04 |
| negative regulation of apoptotic signaling pathway (GO:2001234) | 1.26E-04 |
| transforming growth factor beta receptor superfamily signaling pathway (GO:0141091) | 9.74E-04 |
| Wnt signaling pathway (GO:0016055) | 3.13E-05 |
| regulation of ERK1 and ERK2 cascade (GO:0070372) | 4.69E-05 |
| morphogenesis of an epithelium (GO:0002009) | 1.50E-07 |
| tissue morphogenesis (GO:0048729) | 4.49E-09 |
| negative regulation of hydrolase activity (GO:0051346) | 7.65E-04 |
| regulation of cell migration (GO:0030334) | 3.45E-14 |
| negative regulation of response to external stimulus (GO:0032102) | 1.71E-06 |
| circulatory system development (GO:0072359) | 2.07E-13 |
| positive regulation of MAPK cascade (GO:0043410) | 4.07E-07 |
| positive regulation of nervous system development (GO:0051962) | 5.10E-05 |
| regulation of cell motility (GO:2000145) | 4.13E-14 |
| tube development (GO:0035295) | 9.87E-13 |
| positive regulation of cell migration (GO:0030335) | 5.70E-08 |
| regulation of monoatomic ion transmembrane transport (GO:0034765) | 2.08E-05 |
| regulation of locomotion (GO:0040012) | 3.82E-14 |
| anatomical structure formation involved in morphogenesis (GO:0048646) | 1.86E-12 |
| regulation of peptidyl-tyrosine phosphorylation (GO:0050730) | 1.06E-03 |
| negative regulation of cell population proliferation (GO:0008285) | 3.40E-09 |
| positive regulation of cell motility (GO:2000147) | 1.43E-07 |
| regulation of protein serine/threonine kinase activity (GO:0071900) | 4.47E-04 |
| regulation of phosphatidylinositol 3-kinase/protein kinase B signal transduction (GO:0051896) | 7.49E-04 |
| regulation of monoatomic cation transmembrane transport (GO:1904062) | 2.60E-04 |
| muscle organ development (GO:0007517) | 1.90E-04 |
| positive regulation of locomotion (GO:0040017) | 3.76E-07 |
| calcium ion transport (GO:0006816) | 8.76E-04 |
| regulation of membrane potential (GO:0042391) | 8.78E-06 |
| cell migration (GO:0016477) | 3.18E-10 |
| trans-synaptic signaling (GO:0099537) | 1.94E-05 |
| negative regulation of transport (GO:0051051) | 3.92E-05 |
| modulation of chemical synaptic transmission (GO:0050804) | 1.25E-05 |
| regulation of trans-synaptic signaling (GO:0099177) | 1.28E-05 |
| regulation of MAPK cascade (GO:0043408) | 3.88E-07 |
| regulation of anatomical structure size (GO:0090066) | 9.71E-06 |
| cell junction assembly (GO:0034329) | 8.26E-04 |
| regulation of epithelial cell proliferation (GO:0050678) | 1.81E-04 |
| negative regulation of catalytic activity (GO:0043086) | 7.80E-05 |
| skeletal system development (GO:0001501) | 1.13E-05 |
| regulation of DNA-binding transcription factor activity (GO:0051090) | 2.78E-04 |
| regulation of peptidase activity (GO:0052547) | 9.83E-04 |
| regulation of nervous system development (GO:0051960) | 4.82E-05 |
| positive regulation of secretion by cell (GO:1903532) | 1.02E-03 |
| cell-cell signaling (GO:0007267) | 3.51E-08 |
| gland development (GO:0048732) | 9.79E-05 |
| positive regulation of transport (GO:0051050) | 3.94E-08 |
| regulation of actin filament-based process (GO:0032970) | 2.62E-04 |
| negative regulation of molecular function (GO:0044092) | 9.50E-07 |
| response to wounding (GO:0009611) | 1.14E-04 |
| regulation of protein kinase activity (GO:0045859) | 8.54E-05 |
| chemical synaptic transmission (GO:0007268) | 1.57E-04 |
| anterograde trans-synaptic signaling (GO:0098916) | 1.57E-04 |
| positive regulation of developmental process (GO:0051094) | 8.03E-12 |
| enzyme-linked receptor protein signaling pathway (GO:0007167) | 5.11E-06 |
| synaptic signaling (GO:0099536) | 7.13E-05 |
| cell surface receptor protein tyrosine kinase signaling pathway (GO:0007169) | 1.83E-04 |
| embryonic organ development (GO:0048568) | 1.64E-04 |
| positive regulation of phosphorylation (GO:0042327) | 1.33E-05 |
| cellular response to growth factor stimulus (GO:0071363) | 1.31E-04 |
| response to growth factor (GO:0070848) | 7.37E-05 |
| heart development (GO:0007507) | 2.40E-05 |
| regulation of cell population proliferation (GO:0042127) | 1.26E-13 |
| regulation of kinase activity (GO:0043549) | 7.98E-05 |
| anatomical structure morphogenesis (GO:0009653) | 2.19E-17 |
| regulation of multicellular organismal development (GO:2000026) | 8.13E-11 |
| positive regulation of protein phosphorylation (GO:0001934) | 5.99E-05 |
| cell junction organization (GO:0034330) | 1.17E-04 |
| regulation of cell adhesion (GO:0030155) | 2.87E-06 |
| positive regulation of multicellular organismal process (GO:0051240) | 4.10E-12 |
| positive regulation of cell differentiation (GO:0045597) | 6.96E-07 |
| regulation of anatomical structure morphogenesis (GO:0022603) | 2.04E-06 |
| cell adhesion (GO:0007155) | 3.46E-07 |
| negative regulation of multicellular organismal process (GO:0051241) | 8.63E-08 |
| regulation of hydrolase activity (GO:0051336) | 2.62E-05 |
| cell motility (GO:0048870) | 9.42E-08 |
| endocytosis (GO:0006897) | 3.82E-04 |
| animal organ morphogenesis (GO:0009887) | 6.98E-07 |
| negative regulation of immune system process (GO:0002683) | 3.98E-04 |
| negative regulation of programmed cell death (GO:0043069) | 2.16E-06 |
| regulation of transmembrane transport (GO:0034762) | 9.42E-04 |
| regulation of protein phosphorylation (GO:0001932) | 4.14E-06 |
| regulation of transferase activity (GO:0051338) | 1.31E-04 |
| supramolecular fiber organization (GO:0097435) | 1.82E-04 |
| regulation of secretion by cell (GO:1903530) | 4.03E-04 |
| regulation of phosphorylation (GO:0042325) | 2.48E-06 |
| positive regulation of phosphorus metabolic process (GO:0010562) | 7.58E-05 |
| positive regulation of phosphate metabolic process (GO:0045937) | 7.58E-05 |
| negative regulation of apoptotic process (GO:0043066) | 6.83E-06 |
| regulation of response to external stimulus (GO:0032101) | 7.80E-07 |
| cell population proliferation (GO:0008283) | 6.89E-05 |
| embryonic morphogenesis (GO:0048598) | 4.11E-04 |
| import into cell (GO:0098657) | 1.27E-04 |
| negative regulation of signaling (GO:0023057) | 1.87E-08 |
| regulation of vesicle-mediated transport (GO:0060627) | 1.10E-03 |
| positive regulation of programmed cell death (GO:0043068) | 8.55E-04 |
| metal ion transport (GO:0030001) | 2.08E-04 |
| negative regulation of cell communication (GO:0010648) | 4.43E-08 |
| positive regulation of cell population proliferation (GO:0008284) | 8.98E-06 |
| regulation of transport (GO:0051049) | 6.92E-09 |
| regulation of multicellular organismal process (GO:0051239) | 1.51E-16 |
| regulation of secretion (GO:0051046) | 4.78E-04 |
| cell-cell adhesion (GO:0098609) | 1.07E-03 |
| positive regulation of intracellular signal transduction (GO:1902533) | 1.95E-06 |
| negative regulation of response to stimulus (GO:0048585) | 4.04E-09 |
| positive regulation of molecular function (GO:0044093) | 1.74E-06 |
| cell surface receptor signaling pathway (GO:0007166) | 3.61E-11 |
| inflammatory response (GO:0006954) | 8.73E-04 |
| tissue development (GO:0009888) | 4.01E-09 |
| negative regulation of developmental process (GO:0051093) | 4.76E-05 |
| sensory organ development (GO:0007423) | 1.07E-03 |
| positive regulation of catalytic activity (GO:0043085) | 1.37E-04 |
| regulation of catalytic activity (GO:0050790) | 1.20E-06 |
| negative regulation of signal transduction (GO:0009968) | 1.61E-06 |
| system development (GO:0048731) | 4.80E-17 |
| negative regulation of intracellular signal transduction (GO:1902532) | 1.20E-03 |
| regulation of phosphate metabolic process (GO:0019220) | 1.36E-05 |
| regulation of phosphorus metabolic process (GO:0051174) | 1.38E-05 |
| regulation of developmental process (GO:0050793) | 5.97E-11 |
| positive regulation of cell communication (GO:0010647) | 5.13E-08 |
| regulation of molecular function (GO:0065009) | 1.71E-08 |
| regulation of programmed cell death (GO:0043067) | 9.86E-07 |
| positive regulation of signaling (GO:0023056) | 1.18E-07 |
| animal organ development (GO:0048513) | 7.32E-12 |
| positive regulation of cellular component organization (GO:0051130) | 4.31E-05 |
| regulation of apoptotic process (GO:0042981) | 3.57E-06 |
| positive regulation of signal transduction (GO:0009967) | 2.24E-06 |
| regulation of intracellular signal transduction (GO:1902531) | 1.05E-07 |
| chemical homeostasis (GO:0048878) | 5.06E-04 |
| multicellular organism development (GO:0007275) | 5.82E-15 |
| monoatomic ion transport (GO:0006811) | 4.59E-04 |
| response to external stimulus (GO:0009605) | 7.43E-07 |
| positive regulation of gene expression (GO:0010628) | 1.37E-04 |
| neurogenesis (GO:0022008) | 4.40E-05 |
| response to endogenous stimulus (GO:0009719) | 3.84E-05 |
| regulation of biological quality (GO:0065008) | 1.07E-09 |
| apoptotic process (GO:0006915) | 4.57E-04 |
| epithelium development (GO:0060429) | 3.27E-04 |
| regulation of cell communication (GO:0010646) | 1.29E-11 |
| regulation of localization (GO:0032879) | 9.30E-07 |
| regulation of cell differentiation (GO:0045595) | 2.46E-05 |
| regulation of signaling (GO:0023051) | 1.91E-11 |
| programmed cell death (GO:0012501) | 5.61E-04 |
| cellular response to oxygen-containing compound (GO:1901701) | 8.09E-04 |
| response to oxygen-containing compound (GO:1901700) | 4.19E-05 |
| cell death (GO:0008219) | 5.90E-04 |
| cellular response to chemical stimulus (GO:0070887) | 3.33E-06 |
| regulation of signal transduction (GO:0009966) | 2.26E-09 |
| regulation of protein modification process (GO:0031399) | 4.33E-04 |
| embryo development (GO:0009790) | 9.72E-04 |
| nervous system development (GO:0007399) | 1.03E-06 |
| positive regulation of response to stimulus (GO:0048584) | 9.39E-07 |
| cellular response to endogenous stimulus (GO:0071495) | 9.85E-04 |
| generation of neurons (GO:0048699) | 9.12E-04 |
| system process (GO:0003008) | 1.03E-05 |
| positive regulation of protein metabolic process (GO:0051247) | 9.26E-04 |
| regulation of response to stimulus (GO:0048583) | 2.18E-10 |
| signal transduction (GO:0007165) | 1.28E-12 |
| signaling (GO:0023052) | 1.72E-13 |
| cell communication (GO:0007154) | 2.09E-13 |
| anatomical structure development (GO:0048856) | 3.51E-13 |
| defense response (GO:0006952) | 7.44E-04 |
| intracellular signal transduction (GO:0035556) | 1.01E-03 |
| cell differentiation (GO:0030154) | 4.28E-07 |
| cellular developmental process (GO:0048869) | 4.32E-07 |
| regulation of cellular component organization (GO:0051128) | 8.49E-05 |
| negative regulation of biological process (GO:0048519) | 3.88E-10 |
| developmental process (GO:0032502) | 1.04E-10 |
| negative regulation of cellular process (GO:0048523) | 1.83E-08 |
| multicellular organismal process (GO:0032501) | 8.58E-12 |
| response to chemical (GO:0042221) | 1.66E-05 |
| cellular response to stimulus (GO:0051716) | 7.97E-10 |
| positive regulation of cellular process (GO:0048522) | 2.24E-08 |
| positive regulation of biological process (GO:0048518) | 5.63E-08 |
| response to stimulus (GO:0050896) | 4.12E-10 |
| regulation of biological process (GO:0050789) | 1.48E-10 |
| regulation of cellular process (GO:0050794) | 8.57E-09 |
| biological regulation (GO:0065007) | 2.46E-10 |
| biological_process (GO:0008150) | 5.57E-04 |
| nucleobase-containing compound metabolic process (GO:0006139) | 1.10E-03 |
| Unclassified (UNCLASSIFIED) | 5.57E-04 |
| protein-containing complex organization (GO:0043933) | 4.85E-04 |
| nucleic acid metabolic process (GO:0090304) | 1.05E-04 |
| protein-DNA complex organization (GO:0071824) | 4.72E-04 |
| protein localization to organelle (GO:0033365) | 1.13E-03 |
| RNA processing (GO:0006396) | 1.98E-04 |
| sensory perception of chemical stimulus (GO:0007606) | 4.98E-05 |
| detection of chemical stimulus (GO:0009593) | 1.73E-05 |
| sensory perception of smell (GO:0007608) | 1.00E-05 |
| detection of chemical stimulus involved in sensory perception of smell (GO:0050911) | 1.21E-06 |
| detection of chemical stimulus involved in sensory perception (GO:0050907) | 2.47E-07 |

**Supplementary Table 6. Significant Biological Process Term associated with transcripts found to be downregulated between Saos-2-ADAR2 and Saos2-Empty cells.**

| **GO biological process complete** | **P value** |
| --- | --- |
| glomerular mesangial cell proliferation (GO:0072110) | 1.05E-03 |
| positive regulation of tongue muscle cell differentiation (GO:2001037) | 1.05E-03 |
| regulation of tongue muscle cell differentiation (GO:2001035) | 1.05E-03 |
| cellular response to mycophenolic acid (GO:0071506) | 1.05E-03 |
| response to mycophenolic acid (GO:0071505) | 1.05E-03 |
| cellular response to vitamin K (GO:0071307) | 1.05E-03 |
| trans-synaptic signaling by nitric oxide, modulating synaptic transmission (GO:0099555) | 1.05E-03 |
| trans-synaptic signaling by soluble gas, modulating synaptic transmission (GO:0099554) | 1.05E-03 |
| trans-synaptic signaling by nitric oxide (GO:0099548) | 1.05E-03 |
| trans-synaptic signaling by soluble gas (GO:0099543) | 1.05E-03 |
| vascular endothelial cell proliferation (GO:0101023) | 1.05E-03 |
| synaptic signaling by nitric oxide (GO:0099163) | 1.05E-03 |
| pulmonary myocardium development (GO:0003350) | 1.05E-03 |
| positive regulation of skeletal muscle fiber differentiation (GO:1902811) | 3.37E-05 |
| regulation of skeletal muscle fiber differentiation (GO:1902809) | 1.32E-04 |
| osteoclast proliferation (GO:0002158) | 3.21E-04 |
| B cell chemotaxis (GO:0035754) | 6.27E-04 |
| regulation of fever generation (GO:0031620) | 1.20E-04 |
| positive regulation of glomerular mesangial cell proliferation (GO:0072126) | 1.07E-03 |
| regulation of serotonin secretion (GO:0014062) | 1.07E-03 |
| regulation of fibroblast growth factor production (GO:0090270) | 1.07E-03 |
| positive regulation of odontoblast differentiation (GO:1901331) | 1.07E-03 |
| positive regulation of fever generation (GO:0031622) | 1.07E-03 |
| response to nematode (GO:0009624) | 1.07E-03 |
| cellular response to erythropoietin (GO:0036018) | 1.07E-03 |
| response to erythropoietin (GO:0036017) | 1.07E-03 |
| regulation of bone mineralization involved in bone maturation (GO:1900157) | 1.07E-03 |
| chorio-allantoic fusion (GO:0060710) | 1.07E-03 |
| negative regulation of smooth muscle contraction (GO:0045986) | 2.29E-05 |
| regulation of glomerular mesangial cell proliferation (GO:0072124) | 1.95E-04 |
| cell proliferation involved in kidney development (GO:0072111) | 1.95E-04 |
| glomerular mesangium development (GO:0072109) | 1.67E-03 |
| positive regulation of skeletal muscle cell differentiation (GO:2001016) | 1.67E-03 |
| negative regulation of synaptic transmission, glutamatergic (GO:0051967) | 1.67E-03 |
| negative regulation of plasminogen activation (GO:0010757) | 1.67E-03 |
| kidney vasculature morphogenesis (GO:0061439) | 1.67E-03 |
| renal system vasculature morphogenesis (GO:0061438) | 1.67E-03 |
| positive regulation of neuroepithelial cell differentiation (GO:1902913) | 1.67E-03 |
| regulation of presynaptic cytosolic calcium ion concentration (GO:0099509) | 8.02E-05 |
| negative regulation of muscle contraction (GO:0045932) | 2.82E-06 |
| negative regulation of catecholamine secretion (GO:0033604) | 6.15E-04 |
| negative regulation of extracellular matrix organization (GO:1903054) | 6.15E-04 |
| regulation of heat generation (GO:0031650) | 6.15E-04 |
| membrane depolarization during cardiac muscle cell action potential (GO:0086012) | 1.57E-04 |
| regulation of cell proliferation involved in kidney development (GO:1901722) | 8.40E-04 |
| interneuron migration (GO:1904936) | 8.40E-04 |
| extracellular matrix assembly (GO:0085029) | 3.59E-06 |
| parasympathetic nervous system development (GO:0048486) | 2.79E-04 |
| branching involved in salivary gland morphogenesis (GO:0060445) | 1.45E-03 |
| adenylate cyclase-activating adrenergic receptor signaling pathway (GO:0071880) | 3.62E-04 |
| negative regulation of calcium ion transmembrane transporter activity (GO:1901020) | 9.18E-05 |
| kidney vasculature development (GO:0061440) | 9.18E-05 |
| renal system vasculature development (GO:0061437) | 9.18E-05 |
| tongue development (GO:0043586) | 4.62E-04 |
| negative regulation of SMAD protein signal transduction (GO:0060392) | 5.82E-04 |
| glomerulus vasculature development (GO:0072012) | 5.82E-04 |
| regulation of renal system process (GO:0098801) | 5.82E-04 |
| sympathetic nervous system development (GO:0048485) | 5.82E-04 |
| cyclic nucleotide biosynthetic process (GO:0009190) | 5.82E-04 |
| membrane depolarization during action potential (GO:0086010) | 5.82E-04 |
| negative regulation of chondrocyte differentiation (GO:0032331) | 8.91E-04 |
| forebrain regionalization (GO:0021871) | 8.91E-04 |
| positive regulation of smooth muscle cell migration (GO:0014911) | 2.92E-05 |
| monocyte chemotaxis (GO:0002548) | 1.08E-03 |
| adrenergic receptor signaling pathway (GO:0071875) | 1.08E-03 |
| branching involved in blood vessel morphogenesis (GO:0001569) | 3.43E-04 |
| negative regulation of cartilage development (GO:0061037) | 3.43E-04 |
| dopamine metabolic process (GO:0042417) | 4.14E-04 |
| regulation of smooth muscle contraction (GO:0006940) | 1.79E-06 |
| metanephric nephron development (GO:0072210) | 4.96E-04 |
| aorta morphogenesis (GO:0035909) | 4.96E-04 |
| negative regulation of amine transport (GO:0051953) | 1.56E-03 |
| negative regulation of striated muscle cell apoptotic process (GO:0010664) | 1.56E-03 |
| regulation of smooth muscle cell migration (GO:0014910) | 1.00E-06 |
| vasodilation (GO:0042311) | 2.38E-05 |
| temperature homeostasis (GO:0001659) | 6.97E-04 |
| artery morphogenesis (GO:0048844) | 1.66E-06 |
| autonomic nervous system development (GO:0048483) | 1.01E-04 |
| positive regulation of osteoblast differentiation (GO:0045669) | 1.95E-06 |
| embryonic eye morphogenesis (GO:0048048) | 8.18E-04 |
| cyclic purine nucleotide metabolic process (GO:0052652) | 3.10E-04 |
| catecholamine metabolic process (GO:0006584) | 1.19E-04 |
| catechol-containing compound metabolic process (GO:0009712) | 1.19E-04 |
| positive regulation of synaptic transmission, glutamatergic (GO:0051968) | 9.55E-04 |
| cell adhesion mediated by integrin (GO:0033627) | 3.62E-04 |
| cyclic nucleotide metabolic process (GO:0009187) | 3.62E-04 |
| negative regulation of insulin secretion (GO:0046676) | 3.62E-04 |
| regulation of vasoconstriction (GO:0019229) | 9.28E-06 |
| negative regulation of ion transmembrane transporter activity (GO:0032413) | 6.19E-05 |
| semaphorin-plexin signaling pathway (GO:0071526) | 4.21E-04 |
| collagen metabolic process (GO:0032963) | 3.20E-05 |
| regulation of synaptic transmission, glutamatergic (GO:0051966) | 5.57E-06 |
| negative regulation of blood coagulation (GO:0030195) | 5.60E-04 |
| fear response (GO:0042596) | 1.47E-03 |
| regulation of striated muscle cell apoptotic process (GO:0010662) | 1.47E-03 |
| smooth muscle contraction (GO:0006939) | 1.09E-04 |
| negative regulation of hemostasis (GO:1900047) | 6.43E-04 |
| negative regulation of peptide hormone secretion (GO:0090278) | 7.35E-04 |
| collagen fibril organization (GO:0030199) | 1.42E-04 |
| negative regulation of coagulation (GO:0050819) | 8.36E-04 |
| negative regulation of peptide secretion (GO:0002792) | 8.36E-04 |
| negative regulation of transporter activity (GO:0032410) | 1.62E-04 |
| regulation of blood coagulation (GO:0030193) | 7.15E-05 |
| regulation of extracellular matrix organization (GO:1903053) | 1.83E-04 |
| regulation of signaling receptor activity (GO:0010469) | 3.20E-06 |
| blood vessel diameter maintenance (GO:0097746) | 1.50E-08 |
| regulation of tube diameter (GO:0035296) | 1.50E-08 |
| regulation of hemostasis (GO:1900046) | 9.17E-05 |
| regulation of tube size (GO:0035150) | 1.69E-08 |
| negative regulation of leukocyte migration (GO:0002686) | 1.07E-03 |
| vasculogenesis (GO:0001570) | 1.03E-04 |
| negative regulation of BMP signaling pathway (GO:0030514) | 5.30E-04 |
| regulation of endothelial cell apoptotic process (GO:2000351) | 1.21E-03 |
| regulation of coagulation (GO:0050818) | 1.16E-04 |
| regulation of muscle contraction (GO:0006937) | 1.53E-08 |
| protein localization to synapse (GO:0035418) | 6.68E-04 |
| morphogenesis of a branching epithelium (GO:0061138) | 1.92E-08 |
| branching morphogenesis of an epithelial tube (GO:0048754) | 4.18E-07 |
| response to nicotine (GO:0035094) | 1.52E-03 |
| sprouting angiogenesis (GO:0002040) | 7.48E-04 |
| morphogenesis of a branching structure (GO:0001763) | 1.35E-08 |
| negative regulation of hormone secretion (GO:0046888) | 3.69E-04 |
| positive regulation of ossification (GO:0045778) | 1.70E-03 |
| positive regulation of wound healing (GO:0090303) | 8.35E-04 |
| regulation of epidermal growth factor receptor signaling pathway (GO:0042058) | 8.35E-04 |
| myofibril assembly (GO:0030239) | 4.12E-04 |
| positive regulation of epithelial cell differentiation (GO:0030858) | 9.30E-04 |
| nephron morphogenesis (GO:0072028) | 5.09E-04 |
| ureteric bud development (GO:0001657) | 1.25E-04 |
| striated muscle cell development (GO:0055002) | 5.09E-04 |
| renal tubule morphogenesis (GO:0061333) | 5.09E-04 |
| positive regulation of synapse assembly (GO:0051965) | 1.03E-03 |
| regulation of smooth muscle cell proliferation (GO:0048660) | 2.17E-06 |
| mesonephric tubule development (GO:0072164) | 1.39E-04 |
| mesonephric epithelium development (GO:0072163) | 1.39E-04 |
| kidney morphogenesis (GO:0060993) | 1.39E-04 |
| renal tubule development (GO:0061326) | 1.53E-04 |
| nephron development (GO:0072006) | 2.93E-06 |
| regulation of calcium ion transmembrane transporter activity (GO:1901019) | 1.27E-03 |
| artery development (GO:0060840) | 9.32E-05 |
| positive regulation of endothelial cell proliferation (GO:0001938) | 1.87E-04 |
| positive regulation of muscle cell differentiation (GO:0051149) | 1.40E-03 |
| chondrocyte differentiation (GO:0002062) | 3.76E-04 |
| positive regulation of smooth muscle cell proliferation (GO:0048661) | 3.76E-04 |
| BMP signaling pathway (GO:0030509) | 7.60E-04 |
| regulation of systemic arterial blood pressure (GO:0003073) | 2.06E-04 |
| mesonephros development (GO:0001823) | 2.06E-04 |
| positive regulation of ERK1 and ERK2 cascade (GO:0070374) | 1.69E-07 |
| nephron tubule development (GO:0072080) | 4.14E-04 |
| nephron tubule morphogenesis (GO:0072078) | 1.54E-03 |
| negative regulation of protein secretion (GO:0050709) | 1.54E-03 |
| negative regulation of wound healing (GO:0061045) | 1.54E-03 |
| extracellular matrix organization (GO:0030198) | 4.12E-10 |
| regulation of ERBB signaling pathway (GO:1901184) | 1.69E-03 |
| extracellular structure organization (GO:0043062) | 4.52E-10 |
| regulation of striated muscle contraction (GO:0006942) | 2.49E-04 |
| negative regulation of extrinsic apoptotic signaling pathway (GO:2001237) | 2.49E-04 |
| negative regulation of transmembrane receptor protein serine/threonine kinase signaling pathway (GO:0090101) | 5.73E-06 |
| external encapsulating structure organization (GO:0045229) | 4.96E-10 |
| cellular response to ketone (GO:1901655) | 1.49E-04 |
| positive regulation of protein serine/threonine kinase activity (GO:0071902) | 4.51E-05 |
| regulation of muscle system process (GO:0090257) | 3.02E-08 |
| negative regulation of monoatomic ion transport (GO:0043271) | 8.98E-05 |
| positive regulation of chemotaxis (GO:0050921) | 1.49E-05 |
| neuron projection extension (GO:1990138) | 1.10E-03 |
| regulation of bone mineralization (GO:0030500) | 1.10E-03 |
| positive regulation of synaptic transmission (GO:0050806) | 1.63E-05 |
| negative regulation of secretion by cell (GO:1903531) | 1.78E-05 |
| blood vessel morphogenesis (GO:0048514) | 9.12E-14 |
| cardiac muscle cell differentiation (GO:0055007) | 6.56E-04 |
| positive regulation of cell junction assembly (GO:1901890) | 2.13E-04 |
| regulation of release of sequestered calcium ion into cytosol (GO:0051279) | 1.32E-03 |
| regulation of blood pressure (GO:0008217) | 2.33E-06 |
| primary alcohol metabolic process (GO:0034308) | 7.80E-04 |
| developmental growth involved in morphogenesis (GO:0060560) | 4.19E-05 |
| regulation of osteoblast differentiation (GO:0045667) | 7.63E-05 |
| regulation of blood circulation (GO:1903522) | 4.33E-08 |
| regulation of wound healing (GO:0061041) | 8.30E-05 |
| phenol-containing compound metabolic process (GO:0018958) | 8.49E-04 |
| negative regulation of transforming growth factor beta receptor signaling pathway (GO:0030512) | 8.49E-04 |
| positive regulation of leukocyte chemotaxis (GO:0002690) | 8.49E-04 |
| mononuclear cell migration (GO:0071674) | 2.76E-04 |
| blood circulation (GO:0008015) | 3.63E-12 |
| regulation of ossification (GO:0030278) | 1.64E-04 |
| kidney epithelium development (GO:0072073) | 5.39E-05 |
| metanephros development (GO:0001656) | 1.70E-03 |
| negative regulation of cellular response to growth factor stimulus (GO:0090288) | 5.47E-04 |
| gland morphogenesis (GO:0022612) | 5.94E-04 |
| canonical Wnt signaling pathway (GO:0060070) | 6.44E-04 |
| blood vessel development (GO:0001568) | 7.08E-14 |
| regulation of MAP kinase activity (GO:0043405) | 2.45E-04 |
| angiogenesis (GO:0001525) | 2.04E-09 |
| regulation of leukocyte migration (GO:0002685) | 1.32E-06 |
| regulation of mononuclear cell migration (GO:0071675) | 1.58E-04 |
| tissue remodeling (GO:0048771) | 8.14E-04 |
| negative regulation of T cell activation (GO:0050868) | 2.86E-04 |
| vasculature development (GO:0001944) | 9.71E-14 |
| negative regulation of cell migration (GO:0030336) | 7.61E-08 |
| positive regulation of leukocyte migration (GO:0002687) | 1.09E-04 |
| nephron epithelium development (GO:0072009) | 8.79E-04 |
| response to hexose (GO:0009746) | 1.84E-04 |
| regulation of leukocyte chemotaxis (GO:0002688) | 5.21E-04 |
| locomotion (GO:0040011) | 2.80E-09 |
| striated muscle cell differentiation (GO:0051146) | 3.46E-06 |
| cell chemotaxis (GO:0060326) | 5.78E-06 |
| regulation of biomineral tissue development (GO:0070167) | 1.60E-03 |
| regulation of chemotaxis (GO:0050920) | 1.04E-05 |
| positive regulation of angiogenesis (GO:0045766) | 8.16E-05 |
| regulation of endothelial cell proliferation (GO:0001936) | 3.86E-04 |
| negative regulation of secretion (GO:0051048) | 8.77E-05 |
| positive regulation of monoatomic ion transport (GO:0043270) | 1.20E-05 |
| bone development (GO:0060348) | 2.16E-05 |
| positive regulation of cell-substrate adhesion (GO:0010811) | 7.00E-04 |
| tube morphogenesis (GO:0035239) | 1.67E-15 |
| positive regulation of vasculature development (GO:1904018) | 1.01E-04 |
| response to glucose (GO:0009749) | 4.46E-04 |
| regulation of ERK1 and ERK2 cascade (GO:0070372) | 7.92E-07 |
| cellular anatomical entity morphogenesis (GO:0032989) | 7.52E-04 |
| cellular component assembly involved in morphogenesis (GO:0010927) | 7.52E-04 |
| olefinic compound metabolic process (GO:0120254) | 2.85E-04 |
| regulation of system process (GO:0044057) | 3.11E-12 |
| ameboidal-type cell migration (GO:0001667) | 2.67E-05 |
| negative regulation of cell motility (GO:2000146) | 2.25E-07 |
| negative regulation of cell activation (GO:0050866) | 1.72E-05 |
| connective tissue development (GO:0061448) | 6.60E-06 |
| cardiac chamber morphogenesis (GO:0003206) | 8.07E-04 |
| response to monosaccharide (GO:0034284) | 3.06E-04 |
| muscle cell differentiation (GO:0042692) | 6.28E-07 |
| positive regulation of calcium ion transport (GO:0051928) | 1.36E-03 |
| positive regulation of MAPK cascade (GO:0043410) | 3.59E-10 |
| placenta development (GO:0001890) | 3.52E-04 |
| hemostasis (GO:0007599) | 8.58E-05 |
| circulatory system process (GO:0003013) | 6.82E-11 |
| regulation of angiogenesis (GO:0045765) | 8.26E-07 |
| regulation of body fluid levels (GO:0050878) | 5.76E-08 |
| negative regulation of locomotion (GO:0040013) | 2.33E-07 |
| positive regulation of DNA-binding transcription factor activity (GO:0051091) | 2.42E-05 |
| vascular process in circulatory system (GO:0003018) | 2.46E-06 |
| negative regulation of leukocyte cell-cell adhesion (GO:1903038) | 6.32E-04 |
| cartilage development (GO:0051216) | 1.64E-04 |
| regulation of nervous system process (GO:0031644) | 1.67E-03 |
| cardiocyte differentiation (GO:0035051) | 1.67E-03 |
| negative regulation of cell-cell adhesion (GO:0022408) | 6.73E-05 |
| regulation of vasculature development (GO:1901342) | 1.23E-06 |
| neuron migration (GO:0001764) | 7.23E-04 |
| blood coagulation (GO:0007596) | 1.88E-04 |
| regulation of monoatomic ion transport (GO:0043269) | 2.57E-09 |
| chemotaxis (GO:0006935) | 2.54E-07 |
| regulation of protein serine/threonine kinase activity (GO:0071900) | 9.45E-06 |
| coagulation (GO:0050817) | 2.14E-04 |
| taxis (GO:0042330) | 2.89E-07 |
| cell fate commitment (GO:0045165) | 1.01E-05 |
| regulation of endothelial cell migration (GO:0010594) | 3.57E-04 |
| negative regulation of cell adhesion (GO:0007162) | 3.22E-06 |
| regulation of response to wounding (GO:1903034) | 3.81E-04 |
| renal system development (GO:0072001) | 1.41E-06 |
| negative regulation of response to external stimulus (GO:0032102) | 1.26E-07 |
| transforming growth factor beta receptor superfamily signaling pathway (GO:0141091) | 4.32E-04 |
| response to glucocorticoid (GO:0051384) | 1.57E-03 |
| tube development (GO:0035295) | 5.69E-16 |
| response to carbohydrate (GO:0009743) | 4.60E-04 |
| regulation of metal ion transport (GO:0010959) | 2.67E-07 |
| limb morphogenesis (GO:0035108) | 1.20E-03 |
| appendage morphogenesis (GO:0035107) | 1.20E-03 |
| regulation of calcium ion transport (GO:0051924) | 4.56E-05 |
| regulation of heart contraction (GO:0008016) | 2.40E-04 |
| positive regulation of protein kinase activity (GO:0045860) | 4.84E-05 |
| positive regulation of nervous system development (GO:0051962) | 9.84E-06 |
| regulation of monoatomic ion transmembrane transporter activity (GO:0032412) | 2.55E-04 |
| kidney development (GO:0001822) | 7.11E-06 |
| regulation of peptidyl-tyrosine phosphorylation (GO:0050730) | 1.84E-04 |
| myeloid leukocyte differentiation (GO:0002573) | 1.44E-03 |
| regulation of epithelial cell differentiation (GO:0030856) | 1.44E-03 |
| muscle contraction (GO:0006936) | 9.01E-05 |
| positive regulation of epithelial cell proliferation (GO:0050679) | 3.04E-04 |
| regulation of cell-substrate adhesion (GO:0010810) | 2.32E-04 |
| muscle tissue development (GO:0060537) | 3.37E-06 |
| regulation of peptide transport (GO:0090087) | 3.61E-04 |
| regulation of peptide secretion (GO:0002791) | 3.61E-04 |
| morphogenesis of an epithelium (GO:0002009) | 1.12E-07 |
| regulation of MAPK cascade (GO:0043408) | 3.25E-10 |
| cardiac chamber development (GO:0003205) | 1.16E-03 |
| epithelial tube morphogenesis (GO:0060562) | 1.13E-05 |
| regulation of transmembrane receptor protein serine/threonine kinase signaling pathway (GO:0090092) | 5.25E-05 |
| muscle system process (GO:0003012) | 2.58E-05 |
| regulation of cell migration (GO:0030334) | 1.99E-14 |
| positive regulation of kinase activity (GO:0033674) | 2.72E-05 |
| negative regulation of apoptotic signaling pathway (GO:2001234) | 1.87E-04 |
| regulation of transmembrane transporter activity (GO:0022898) | 4.05E-04 |
| cell surface receptor protein serine/threonine kinase signaling pathway (GO:0007178) | 8.80E-04 |
| positive regulation of cell migration (GO:0030335) | 1.10E-08 |
| regulation of transporter activity (GO:0032409) | 2.91E-04 |
| muscle cell development (GO:0055001) | 1.37E-03 |
| regulation of hormone secretion (GO:0046883) | 1.08E-04 |
| negative regulation of hydrolase activity (GO:0051346) | 1.04E-03 |
| regulation of protein kinase activity (GO:0045859) | 4.68E-07 |
| limb development (GO:0060173) | 1.10E-03 |
| appendage development (GO:0048736) | 1.10E-03 |
| epithelial cell proliferation (GO:0050673) | 1.62E-03 |
| striated muscle tissue development (GO:0014706) | 7.85E-04 |
| axon guidance (GO:0007411) | 3.82E-04 |
| positive regulation of cell motility (GO:2000147) | 2.08E-08 |
| ossification (GO:0001503) | 6.54E-05 |
| neuron projection guidance (GO:0097485) | 4.03E-04 |
| regulation of cell motility (GO:2000145) | 6.60E-14 |
| circulatory system development (GO:0072359) | 6.45E-13 |
| negative regulation of leukocyte activation (GO:0002695) | 1.22E-03 |
| positive regulation of cell differentiation (GO:0045597) | 4.58E-12 |
| regulation of peptide hormone secretion (GO:0090276) | 8.74E-04 |
| regulation of neuron apoptotic process (GO:0043523) | 3.05E-04 |
| muscle structure development (GO:0061061) | 1.63E-07 |
| tissue morphogenesis (GO:0048729) | 3.82E-08 |
| modulation of chemical synaptic transmission (GO:0050804) | 5.07E-07 |
| regulation of trans-synaptic signaling (GO:0099177) | 5.24E-07 |
| positive regulation of secretion by cell (GO:1903532) | 8.07E-05 |
| lung development (GO:0030324) | 1.36E-03 |
| sensory system development (GO:0048880) | 7.28E-06 |
| regulation of locomotion (GO:0040012) | 8.92E-14 |
| positive regulation of locomotion (GO:0040017) | 3.60E-08 |
| respiratory system development (GO:0060541) | 7.32E-04 |
| eye development (GO:0001654) | 1.13E-05 |
| cell-cell signaling (GO:0007267) | 6.68E-11 |
| hormone metabolic process (GO:0042445) | 1.13E-03 |
| visual system development (GO:0150063) | 1.28E-05 |
| camera-type eye development (GO:0043010) | 5.50E-05 |
| respiratory tube development (GO:0030323) | 1.67E-03 |
| response to mechanical stimulus (GO:0009612) | 8.53E-04 |
| positive regulation of developmental process (GO:0051094) | 1.86E-16 |
| regulation of kinase activity (GO:0043549) | 6.15E-07 |
| response to wounding (GO:0009611) | 5.03E-06 |
| positive regulation of secretion (GO:0051047) | 1.23E-04 |
| regulation of epithelial cell proliferation (GO:0050678) | 5.02E-05 |
| regulation of nervous system development (GO:0051960) | 4.96E-06 |
| regulation of hormone levels (GO:0010817) | 7.83E-07 |
| anatomical structure formation involved in morphogenesis (GO:0048646) | 1.90E-11 |
| adenylate cyclase-modulating G protein-coupled receptor signaling pathway (GO:0007188) | 1.25E-03 |
| regulation of phosphatidylinositol 3-kinase/protein kinase B signal transduction (GO:0051896) | 8.94E-04 |
| regulation of synapse structure or activity (GO:0050803) | 8.94E-04 |
| Wnt signaling pathway (GO:0016055) | 4.53E-04 |
| regulation of leukocyte differentiation (GO:1902105) | 1.77E-04 |
| skeletal system development (GO:0001501) | 4.05E-06 |
| muscle organ development (GO:0007517) | 2.58E-04 |
| sensory organ development (GO:0007423) | 5.88E-07 |
| positive regulation of protein phosphorylation (GO:0001934) | 1.26E-06 |
| regulation of secretion by cell (GO:1903530) | 1.77E-06 |
| gland development (GO:0048732) | 3.48E-05 |
| regulation of multicellular organismal development (GO:2000026) | 8.92E-15 |
| negative regulation of cell population proliferation (GO:0008285) | 8.25E-08 |
| regulation of small GTPase mediated signal transduction (GO:0051056) | 4.22E-04 |
| sensory organ morphogenesis (GO:0090596) | 8.10E-04 |
| learning or memory (GO:0007611) | 8.10E-04 |
| negative regulation of multicellular organismal process (GO:0051241) | 2.44E-11 |
| regulation of synapse organization (GO:0050807) | 1.57E-03 |
| negative regulation of catalytic activity (GO:0043086) | 5.40E-05 |
| regulation of monoatomic ion transmembrane transport (GO:0034765) | 2.50E-04 |
| positive regulation of transferase activity (GO:0051347) | 2.15E-04 |
| enzyme-linked receptor protein signaling pathway (GO:0007167) | 1.03E-06 |
| cognition (GO:0050890) | 3.50E-04 |
| cell surface receptor protein tyrosine kinase signaling pathway (GO:0007169) | 6.12E-05 |
| synapse organization (GO:0050808) | 4.53E-04 |
| positive regulation of phosphorylation (GO:0042327) | 1.72E-06 |
| positive regulation of cell development (GO:0010720) | 3.59E-05 |
| regulation of anatomical structure size (GO:0090066) | 1.61E-05 |
| regulation of cellular response to growth factor stimulus (GO:0090287) | 9.50E-04 |
| neuron projection morphogenesis (GO:0048812) | 2.42E-05 |
| regulation of secretion (GO:0051046) | 3.04E-06 |
| cell migration (GO:0016477) | 7.78E-09 |
| response to growth factor (GO:0070848) | 2.88E-05 |
| trans-synaptic signaling (GO:0099537) | 8.91E-05 |
| plasma membrane bounded cell projection morphogenesis (GO:0120039) | 4.07E-05 |
| regulation of DNA-binding transcription factor activity (GO:0051090) | 5.41E-04 |
| negative regulation of immune system process (GO:0002683) | 3.00E-05 |
| negative regulation of transport (GO:0051051) | 1.74E-04 |
| cell adhesion (GO:0007155) | 9.95E-09 |
| cell projection morphogenesis (GO:0048858) | 4.52E-05 |
| wound healing (GO:0042060) | 1.10E-03 |
| positive regulation of multicellular organismal process (GO:0051240) | 1.91E-14 |
| regulation of cell population proliferation (GO:0042127) | 2.94E-14 |
| negative regulation of molecular function (GO:0044092) | 2.34E-06 |
| cellular response to growth factor stimulus (GO:0071363) | 9.57E-05 |
| animal organ morphogenesis (GO:0009887) | 1.50E-08 |
| cell junction organization (GO:0034330) | 3.23E-05 |
| regulation of transferase activity (GO:0051338) | 1.13E-05 |
| response to oxygen levels (GO:0070482) | 1.28E-03 |
| cell morphogenesis (GO:0000902) | 4.41E-06 |
| negative regulation of developmental process (GO:0051093) | 1.06E-07 |
| regulation of protein phosphorylation (GO:0001932) | 1.61E-07 |
| positive regulation of programmed cell death (GO:0043068) | 7.93E-05 |
| leukocyte differentiation (GO:0002521) | 4.04E-04 |
| positive regulation of transport (GO:0051050) | 6.32E-07 |
| anatomical structure morphogenesis (GO:0009653) | 3.43E-17 |
| chemical synaptic transmission (GO:0007268) | 6.19E-04 |
| anterograde trans-synaptic signaling (GO:0098916) | 6.19E-04 |
| negative regulation of cell communication (GO:0010648) | 1.83E-10 |
| negative regulation of signaling (GO:0023057) | 1.86E-10 |
| synaptic signaling (GO:0099536) | 4.25E-04 |
| positive regulation of apoptotic process (GO:0043065) | 1.82E-04 |
| inflammatory response (GO:0006954) | 6.32E-05 |
| negative regulation of programmed cell death (GO:0043069) | 4.34E-07 |
| negative regulation of apoptotic process (GO:0043066) | 7.85E-07 |
| positive regulation of phosphorus metabolic process (GO:0010562) | 1.42E-05 |
| positive regulation of phosphate metabolic process (GO:0045937) | 1.42E-05 |
| regulation of phosphorylation (GO:0042325) | 2.96E-07 |
| cell-cell adhesion (GO:0098609) | 1.89E-04 |
| regulation of hydrolase activity (GO:0051336) | 3.18E-05 |
| system development (GO:0048731) | 2.15E-25 |
| tissue development (GO:0009888) | 9.24E-12 |
| negative regulation of response to stimulus (GO:0048585) | 5.68E-11 |
| regulation of anatomical structure morphogenesis (GO:0022603) | 4.94E-06 |
| regulation of response to external stimulus (GO:0032101) | 2.72E-07 |
| cell population proliferation (GO:0008283) | 2.35E-05 |
| inorganic ion homeostasis (GO:0098771) | 1.30E-03 |
| regulation of phosphate metabolic process (GO:0019220) | 1.28E-07 |
| regulation of cell adhesion (GO:0030155) | 1.18E-05 |
| regulation of phosphorus metabolic process (GO:0051174) | 1.31E-07 |
| regulation of developmental process (GO:0050793) | 1.46E-15 |
| regulation of transport (GO:0051049) | 2.88E-10 |
| heart development (GO:0007507) | 2.45E-04 |
| embryonic organ development (GO:0048568) | 1.07E-03 |
| negative regulation of signal transduction (GO:0009968) | 1.66E-08 |
| regulation of neuron projection development (GO:0010975) | 1.42E-03 |
| regulation of membrane potential (GO:0042391) | 1.17E-03 |
| regulation of cell development (GO:0060284) | 1.68E-05 |
| regulation of cell differentiation (GO:0045595) | 1.83E-09 |
| embryonic morphogenesis (GO:0048598) | 3.15E-04 |
| regulation of multicellular organismal process (GO:0051239) | 4.73E-18 |
| cell motility (GO:0048870) | 6.41E-07 |
| regulation of transmembrane transport (GO:0034762) | 1.26E-03 |
| regulation of cell activation (GO:0050865) | 2.02E-04 |
| positive regulation of protein modification process (GO:0031401) | 7.35E-05 |
| regulation of biological quality (GO:0065008) | 8.40E-17 |
| positive regulation of cell population proliferation (GO:0008284) | 7.05E-06 |
| animal organ development (GO:0048513) | 6.58E-17 |
| regulation of catalytic activity (GO:0050790) | 7.70E-08 |
| cell surface receptor signaling pathway (GO:0007166) | 6.78E-12 |
| cellular response to cytokine stimulus (GO:0071345) | 1.39E-04 |
| positive regulation of molecular function (GO:0044093) | 1.07E-06 |
| regulation of apoptotic process (GO:0042981) | 3.35E-08 |
| multicellular organism development (GO:0007275) | 2.87E-23 |
| regulation of lymphocyte activation (GO:0051249) | 1.31E-03 |
| cellular response to oxygen-containing compound (GO:1901701) | 6.07E-06 |
| positive regulation of response to external stimulus (GO:0032103) | 8.88E-04 |
| regulation of programmed cell death (GO:0043067) | 2.26E-08 |
| neuron projection development (GO:0031175) | 2.58E-04 |
| response to external stimulus (GO:0009605) | 3.30E-10 |
| positive regulation of intracellular signal transduction (GO:1902533) | 2.77E-06 |
| generation of neurons (GO:0048699) | 2.04E-06 |
| positive regulation of cell communication (GO:0010647) | 4.15E-09 |
| epithelium development (GO:0060429) | 4.06E-06 |
| positive regulation of catalytic activity (GO:0043085) | 7.35E-05 |
| neurogenesis (GO:0022008) | 3.62E-07 |
| nervous system development (GO:0007399) | 2.73E-11 |
| regulation of molecular function (GO:0065009) | 8.89E-10 |
| behavior (GO:0007610) | 7.11E-04 |
| neuron differentiation (GO:0030182) | 7.99E-06 |
| intracellular chemical homeostasis (GO:0055082) | 1.55E-03 |
| regulation of leukocyte activation (GO:0002694) | 1.55E-03 |
| response to peptide (GO:1901652) | 1.15E-04 |
| positive regulation of signaling (GO:0023056) | 7.08E-09 |
| response to cytokine (GO:0034097) | 1.56E-04 |
| cellular response to endogenous stimulus (GO:0071495) | 7.34E-06 |
| response to endogenous stimulus (GO:0009719) | 7.55E-07 |
| response to abiotic stimulus (GO:0009628) | 1.01E-05 |
| negative regulation of cell differentiation (GO:0045596) | 9.24E-04 |
| response to hormone (GO:0009725) | 2.76E-04 |
| cellular response to chemical stimulus (GO:0070887) | 3.60E-09 |
| hemopoiesis (GO:0030097) | 6.23E-04 |
| response to oxygen-containing compound (GO:1901700) | 4.68E-07 |
| response to organic cyclic compound (GO:0014070) | 2.91E-04 |
| central nervous system development (GO:0007417) | 6.94E-05 |
| regulation of localization (GO:0032879) | 9.85E-09 |
| neuron development (GO:0048666) | 2.37E-04 |
| regulation of protein modification process (GO:0031399) | 1.53E-05 |
| embryo development (GO:0009790) | 6.58E-05 |
| regulation of cell communication (GO:0010646) | 8.53E-15 |
| regulation of signaling (GO:0023051) | 1.30E-14 |
| positive regulation of signal transduction (GO:0009967) | 1.03E-06 |
| head development (GO:0060322) | 8.56E-04 |
| regulation of signal transduction (GO:0009966) | 1.37E-12 |
| brain development (GO:0007420) | 1.26E-03 |
| response to lipid (GO:0033993) | 6.36E-04 |
| regulation of intracellular signal transduction (GO:1902531) | 7.61E-08 |
| chemical homeostasis (GO:0048878) | 6.12E-04 |
| anatomical structure development (GO:0048856) | 1.23E-21 |
| cell differentiation (GO:0030154) | 8.48E-14 |
| cellular developmental process (GO:0048869) | 8.74E-14 |
| defense response (GO:0006952) | 1.40E-05 |
| intracellular signaling cassette (GO:0141124) | 1.22E-03 |
| programmed cell death (GO:0012501) | 3.53E-04 |
| cell death (GO:0008219) | 4.93E-04 |
| positive regulation of response to stimulus (GO:0048584) | 2.56E-07 |
| cell development (GO:0048468) | 3.29E-07 |
| apoptotic process (GO:0006915) | 1.07E-03 |
| positive regulation of cellular component organization (GO:0051130) | 6.41E-04 |
| system process (GO:0003008) | 2.82E-06 |
| positive regulation of protein metabolic process (GO:0051247) | 4.30E-04 |
| regulation of response to stimulus (GO:0048583) | 6.40E-12 |
| developmental process (GO:0032502) | 6.64E-18 |
| multicellular organismal process (GO:0032501) | 4.21E-20 |
| signal transduction (GO:0007165) | 5.41E-14 |
| signaling (GO:0023052) | 3.70E-15 |
| cell communication (GO:0007154) | 3.00E-15 |
| plasma membrane bounded cell projection organization (GO:0120036) | 1.40E-03 |
| intracellular signal transduction (GO:0035556) | 7.62E-05 |
| regulation of immune system process (GO:0002682) | 1.97E-04 |
| cell projection organization (GO:0030030) | 1.30E-03 |
| negative regulation of biological process (GO:0048519) | 5.87E-14 |
| negative regulation of cellular process (GO:0048523) | 3.11E-12 |
| homeostatic process (GO:0042592) | 1.42E-03 |
| response to chemical (GO:0042221) | 9.41E-08 |
| regulation of protein metabolic process (GO:0051246) | 1.55E-04 |
| regulation of cellular component organization (GO:0051128) | 1.38E-04 |
| positive regulation of cellular process (GO:0048522) | 2.68E-10 |
| cellular response to stimulus (GO:0051716) | 1.11E-11 |
| positive regulation of biological process (GO:0048518) | 3.76E-10 |
| response to stress (GO:0006950) | 8.44E-05 |
| response to stimulus (GO:0050896) | 4.00E-12 |
| negative regulation of metabolic process (GO:0009892) | 1.31E-03 |
| regulation of cellular process (GO:0050794) | 1.51E-12 |
| regulation of biological process (GO:0050789) | 9.79E-14 |
| biological regulation (GO:0065007) | 3.42E-14 |
| cellular process (GO:0009987) | 3.30E-06 |
| biological_process (GO:0008150) | 8.81E-07 |
| organelle organization (GO:0006996) | 6.21E-04 |
| nucleobase-containing compound metabolic process (GO:0006139) | 6.88E-04 |
| protein-containing complex organization (GO:0043933) | 1.55E-03 |
| nucleic acid metabolic process (GO:0090304) | 3.41E-05 |
| Unclassified (UNCLASSIFIED) | 8.81E-07 |
| protein-containing complex assembly (GO:0065003) | 1.00E-03 |
| cell cycle (GO:0007049) | 1.10E-03 |
| sexual reproduction (GO:0019953) | 6.17E-04 |
| RNA processing (GO:0006396) | 3.58E-04 |
| DNA metabolic process (GO:0006259) | 6.27E-04 |
| mRNA metabolic process (GO:0016071) | 1.51E-03 |
| protein localization to organelle (GO:0033365) | 1.88E-04 |
| cell division (GO:0051301) | 1.01E-03 |
| sensory perception of chemical stimulus (GO:0007606) | 7.59E-04 |
| ribonucleoprotein complex biogenesis (GO:0022613) | 1.77E-03 |
| mRNA processing (GO:0006397) | 1.79E-03 |
| chromosome organization (GO:0051276) | 1.29E-03 |
| DNA repair (GO:0006281) | 3.46E-04 |
| sensory perception of smell (GO:0007608) | 5.06E-05 |
| detection of chemical stimulus (GO:0009593) | 1.24E-05 |
| establishment of protein localization to organelle (GO:0072594) | 2.91E-04 |
| detection of chemical stimulus involved in sensory perception of smell (GO:0050911) | 1.46E-05 |
| detection of chemical stimulus involved in sensory perception (GO:0050907) | 3.19E-06 |
| double-strand break repair (GO:0006302) | 1.50E-03 |

**Supplementary Table 7. Significant Biological Process Term associated with transcripts found to be upregulated between Saos-2-ADAR2 and Saos2-E/A cells.**

| **GO Biological Process** | **p value** |
| --- | --- |
| negative regulation of integrin biosynthetic process (GO:0045720) | 6.85E-04 |
| antigen processing and presentation of endogenous peptide antigen via MHC class II (GO:0002491) | 6.85E-04 |
| fibroblast growth factor receptor apoptotic signaling pathway (GO:1902178) | 6.85E-04 |
| female genitalia morphogenesis (GO:0048807) | 1.79E-05 |
| synaptic signaling via neuropeptide (GO:0099538) | 1.72E-04 |
| regulation of branching involved in prostate gland morphogenesis (GO:0060687) | 1.72E-04 |
| neural crest cell fate commitment (GO:0014034) | 3.37E-04 |
| regulation of removal of superoxide radicals (GO:2000121) | 3.37E-04 |
| bundle of His cell-Purkinje myocyte adhesion involved in cell communication (GO:0086073) | 3.37E-04 |
| prostate epithelial cord arborization involved in prostate glandular acinus morphogenesis (GO:0060527) | 3.37E-04 |
| prostate glandular acinus morphogenesis (GO:0060526) | 3.37E-04 |
| type II pneumocyte differentiation (GO:0060510) | 3.37E-04 |
| fatty acid elongation, unsaturated fatty acid (GO:0019368) | 5.78E-04 |
| hematopoietic stem cell migration (GO:0035701) | 5.78E-04 |
| cardiac muscle cell-cardiac muscle cell adhesion (GO:0086042) | 5.78E-04 |
| fatty acid elongation, polyunsaturated fatty acid (GO:0034626) | 5.78E-04 |
| fatty acid elongation, monounsaturated fatty acid (GO:0034625) | 5.78E-04 |
| mammary gland branching involved in pregnancy (GO:0060745) | 5.78E-04 |
| pancreatic A cell differentiation (GO:0003310) | 8.62E-05 |
| prostate glandular acinus development (GO:0060525) | 8.62E-05 |
| detection of calcium ion (GO:0005513) | 1.31E-05 |
| fatty acid elongation, saturated fatty acid (GO:0019367) | 9.07E-04 |
| ventricular compact myocardium morphogenesis (GO:0003223) | 9.07E-04 |
| lateral sprouting from an epithelium (GO:0060601) | 9.07E-04 |
| collagen-activated tyrosine kinase receptor signaling pathway (GO:0038063) | 1.33E-04 |
| collagen-activated signaling pathway (GO:0038065) | 2.92E-05 |
| regulation of ventricular cardiac muscle cell action potential (GO:0098911) | 1.95E-04 |
| genitalia morphogenesis (GO:0035112) | 1.95E-04 |
| cardiac left ventricle morphogenesis (GO:0003214) | 4.16E-05 |
| positive regulation of phagocytosis, engulfment (GO:0060100) | 2.76E-04 |
| positive regulation of membrane invagination (GO:1905155) | 2.76E-04 |
| prostate gland epithelium morphogenesis (GO:0060740) | 1.38E-06 |
| cellular response to purine-containing compound (GO:0071415) | 3.78E-04 |
| regulation of thymocyte apoptotic process (GO:0070243) | 3.78E-04 |
| prostate gland morphogenesis (GO:0060512) | 2.59E-06 |
| neuron projection arborization (GO:0140058) | 7.81E-05 |
| morphogenesis of an epithelial fold (GO:0060571) | 7.81E-05 |
| regulation of phagocytosis, engulfment (GO:0060099) | 5.05E-04 |
| sarcoplasmic reticulum calcium ion transport (GO:0070296) | 5.05E-04 |
| regulation of membrane invagination (GO:1905153) | 5.05E-04 |
| positive regulation of neuroinflammatory response (GO:0150078) | 5.05E-04 |
| female genitalia development (GO:0030540) | 1.04E-04 |
| preganglionic parasympathetic fiber development (GO:0021783) | 8.44E-04 |
| vascular associated smooth muscle cell differentiation (GO:0035886) | 1.06E-03 |
| transmission of nerve impulse (GO:0019226) | 3.96E-09 |
| smooth muscle cell differentiation (GO:0051145) | 3.51E-05 |
| synaptic membrane adhesion (GO:0099560) | 5.04E-04 |
| protein localization to cell surface (GO:0034394) | 1.59E-04 |
| epithelial cell maturation (GO:0002070) | 6.06E-04 |
| heart trabecula morphogenesis (GO:0061384) | 1.90E-04 |
| glandular epithelial cell development (GO:0002068) | 7.22E-04 |
| regulation of cardiac muscle cell action potential (GO:0098901) | 7.22E-04 |
| enteroendocrine cell differentiation (GO:0035883) | 7.22E-04 |
| regulation of mast cell degranulation (GO:0043304) | 7.22E-04 |
| urogenital system development (GO:0001655) | 6.45E-07 |
| prostate gland development (GO:0030850) | 2.28E-05 |
| regulation of platelet aggregation (GO:0090330) | 8.54E-04 |
| regulation of presynaptic membrane potential (GO:0099505) | 8.54E-04 |
| cardiac ventricle morphogenesis (GO:0003208) | 2.46E-07 |
| negative regulation of synaptic transmission (GO:0050805) | 8.44E-05 |
| genitalia development (GO:0048806) | 2.69E-05 |
| neuronal action potential (GO:0019228) | 1.00E-03 |
| glandular epithelial cell differentiation (GO:0002067) | 5.13E-06 |
| cellular response to BMP stimulus (GO:0071773) | 7.61E-08 |
| response to BMP (GO:0071772) | 7.61E-08 |
| cardiac muscle cell contraction (GO:0086003) | 4.97E-05 |
| outflow tract morphogenesis (GO:0003151) | 7.20E-07 |
| cardiac muscle cell action potential involved in contraction (GO:0086002) | 1.56E-04 |
| autonomic nervous system development (GO:0048483) | 1.79E-04 |
| trabecula morphogenesis (GO:0061383) | 1.79E-04 |
| cardiac chamber morphogenesis (GO:0003206) | 7.61E-10 |
| ventricular cardiac muscle tissue morphogenesis (GO:0055010) | 2.06E-04 |
| coronary vasculature development (GO:0060976) | 2.06E-04 |
| cell communication involved in cardiac conduction (GO:0086065) | 7.39E-04 |
| gland morphogenesis (GO:0022612) | 9.82E-08 |
| regulation of bone resorption (GO:0045124) | 8.40E-04 |
| regulation of inflammatory response to antigenic stimulus (GO:0002861) | 8.40E-04 |
| regulation of bone remodeling (GO:0046850) | 3.47E-04 |
| cardiac chamber development (GO:0003205) | 9.27E-11 |
| regulation of dendritic spine morphogenesis (GO:0061001) | 9.51E-04 |
| neuromuscular junction development (GO:0007528) | 9.51E-04 |
| adenylate cyclase-inhibiting G protein-coupled receptor signaling pathway (GO:0007193) | 2.51E-05 |
| cardiac muscle tissue morphogenesis (GO:0055008) | 1.62E-04 |
| regulation of morphogenesis of a branching structure (GO:0060688) | 4.41E-04 |
| odontogenesis of dentin-containing tooth (GO:0042475) | 1.19E-05 |
| muscle organ morphogenesis (GO:0048644) | 3.56E-05 |
| ventricular cardiac muscle tissue development (GO:0003229) | 2.30E-04 |
| cardiac septum morphogenesis (GO:0060411) | 9.54E-05 |
| cardiac ventricle development (GO:0003231) | 2.20E-07 |
| cardiac muscle cell action potential (GO:0086001) | 6.21E-04 |
| positive regulation of synapse assembly (GO:0051965) | 2.56E-04 |
| homotypic cell-cell adhesion (GO:0034109) | 2.86E-04 |
| columnar/cuboidal epithelial cell differentiation (GO:0002065) | 4.52E-06 |
| cardiac conduction (GO:0061337) | 3.53E-04 |
| regulation of lymphocyte apoptotic process (GO:0070228) | 8.54E-04 |
| actin-mediated cell contraction (GO:0070252) | 1.63E-04 |
| BMP signaling pathway (GO:0030509) | 1.63E-04 |
| response to calcium ion (GO:0051592) | 1.80E-06 |
| pancreas development (GO:0031016) | 4.77E-04 |
| regulation of postsynapse organization (GO:0099175) | 1.97E-05 |
| muscle tissue morphogenesis (GO:0060415) | 5.25E-04 |
| cardiac septum development (GO:0003279) | 2.39E-05 |
| action potential (GO:0001508) | 5.16E-06 |
| cellular response to calcium ion (GO:0071277) | 6.34E-04 |
| synapse assembly (GO:0007416) | 1.47E-05 |
| heart morphogenesis (GO:0003007) | 2.27E-10 |
| cardiac muscle contraction (GO:0060048) | 3.19E-04 |
| regulation of tissue remodeling (GO:0034103) | 6.95E-04 |
| regulation of synapse assembly (GO:0051963) | 1.76E-05 |
| heart process (GO:0003015) | 4.13E-05 |
| heart contraction (GO:0060047) | 2.70E-04 |
| regulation of heart rate (GO:0002027) | 2.93E-04 |
| actin filament-based movement (GO:0030048) | 3.18E-04 |
| detection of external stimulus (GO:0009581) | 4.44E-05 |
| odontogenesis (GO:0042476) | 8.77E-05 |
| female sex differentiation (GO:0046660) | 1.73E-04 |
| adenylate cyclase-activating G protein-coupled receptor signaling pathway (GO:0007189) | 1.24E-05 |
| regulation of fat cell differentiation (GO:0045598) | 4.80E-05 |
| locomotory behavior (GO:0007626) | 1.13E-06 |
| cell-cell adhesion (GO:0098609) | 1.01E-15 |
| cell-matrix adhesion (GO:0007160) | 7.05E-05 |
| positive regulation of inflammatory response (GO:0050729) | 3.86E-05 |
| positive regulation of cytosolic calcium ion concentration (GO:0007204) | 2.63E-05 |
| striated muscle contraction (GO:0006941) | 3.18E-04 |
| adenylate cyclase-modulating G protein-coupled receptor signaling pathway (GO:0007188) | 4.23E-07 |
| neuropeptide signaling pathway (GO:0007218) | 6.76E-04 |
| detection of abiotic stimulus (GO:0009582) | 2.30E-04 |
| regulation of synapse organization (GO:0050807) | 7.38E-07 |
| developmental growth involved in morphogenesis (GO:0060560) | 2.82E-04 |
| regulation of synapse structure or activity (GO:0050803) | 1.10E-06 |
| morphogenesis of a branching structure (GO:0001763) | 6.42E-05 |
| morphogenesis of a branching epithelium (GO:0061138) | 1.17E-04 |
| regulation of blood circulation (GO:1903522) | 2.77E-06 |
| positive regulation of peptidyl-tyrosine phosphorylation (GO:0050731) | 2.28E-04 |
| epithelial cell development (GO:0002064) | 4.55E-05 |
| synapse organization (GO:0050808) | 8.47E-08 |
| renal system development (GO:0072001) | 2.63E-07 |
| calcium-mediated signaling (GO:0019722) | 3.11E-04 |
| regulation of heart contraction (GO:0008016) | 6.60E-05 |
| cell-substrate adhesion (GO:0031589) | 1.13E-04 |
| cell junction assembly (GO:0034329) | 2.00E-06 |
| axon guidance (GO:0007411) | 2.71E-05 |
| neuron projection guidance (GO:0097485) | 2.88E-05 |
| limb morphogenesis (GO:0035108) | 6.02E-04 |
| appendage morphogenesis (GO:0035107) | 6.02E-04 |
| extracellular matrix organization (GO:0030198) | 3.83E-06 |
| regulation of neuron differentiation (GO:0045664) | 1.35E-04 |
| extracellular structure organization (GO:0043062) | 4.06E-06 |
| external encapsulating structure organization (GO:0045229) | 4.31E-06 |
| central nervous system neuron differentiation (GO:0021953) | 2.45E-04 |
| regulation of epithelial cell differentiation (GO:0030856) | 7.18E-04 |
| positive regulation of neuron projection development (GO:0010976) | 8.53E-04 |
| axonogenesis (GO:0007409) | 4.63E-07 |
| muscle system process (GO:0003012) | 8.49E-06 |
| cell-cell adhesion via plasma-membrane adhesion molecules (GO:0098742) | 2.20E-05 |
| kidney development (GO:0001822) | 5.89E-06 |
| regulation of neuron projection development (GO:0010975) | 5.91E-08 |
| gland development (GO:0048732) | 9.53E-08 |
| cell adhesion (GO:0007155) | 6.28E-16 |
| cell junction organization (GO:0034330) | 2.69E-09 |
| cell projection morphogenesis (GO:0048858) | 1.20E-08 |
| neuron projection morphogenesis (GO:0048812) | 2.27E-08 |
| epithelial tube morphogenesis (GO:0060562) | 9.01E-06 |
| plasma membrane bounded cell projection morphogenesis (GO:0120039) | 2.95E-08 |
| animal organ morphogenesis (GO:0009887) | 5.40E-15 |
| negative regulation of cell projection organization (GO:0031345) | 9.02E-04 |
| tissue morphogenesis (GO:0048729) | 4.97E-09 |
| positive regulation of nervous system development (GO:0051962) | 3.01E-05 |
| muscle contraction (GO:0006936) | 1.72E-04 |
| anatomical structure maturation (GO:0071695) | 1.72E-04 |
| regulation of peptidyl-tyrosine phosphorylation (GO:0050730) | 4.70E-04 |
| reproductive structure development (GO:0048608) | 3.86E-05 |
| vascular process in circulatory system (GO:0003018) | 9.46E-05 |
| tube morphogenesis (GO:0035239) | 5.61E-10 |
| morphogenesis of an epithelium (GO:0002009) | 4.89E-07 |
| reproductive system development (GO:0061458) | 4.69E-05 |
| cell morphogenesis involved in neuron differentiation (GO:0048667) | 7.85E-07 |
| mesenchyme development (GO:0060485) | 2.56E-04 |
| axon development (GO:0061564) | 2.33E-06 |
| regulation of cell junction assembly (GO:1901888) | 6.92E-04 |
| blood circulation (GO:0008015) | 4.48E-06 |
| circulatory system process (GO:0003013) | 4.65E-07 |
| sex differentiation (GO:0007548) | 1.67E-04 |
| regulation of nervous system development (GO:0051960) | 3.17E-06 |
| regulation of ERK1 and ERK2 cascade (GO:0070372) | 2.92E-04 |
| response to hypoxia (GO:0001666) | 2.19E-04 |
| heart development (GO:0007507) | 3.32E-07 |
| negative regulation of cell development (GO:0010721) | 5.81E-04 |
| tube development (GO:0035295) | 2.74E-10 |
| regulation of epithelial cell proliferation (GO:0050678) | 7.47E-05 |
| positive regulation of cell-cell adhesion (GO:0022409) | 1.52E-04 |
| regulation of system process (GO:0044057) | 1.53E-06 |
| cell morphogenesis (GO:0000902) | 5.66E-08 |
| neuron projection development (GO:0031175) | 6.87E-08 |
| regulation of cell projection organization (GO:0031344) | 2.33E-07 |
| developmental maturation (GO:0021700) | 5.57E-04 |
| response to decreased oxygen levels (GO:0036293) | 3.98E-04 |
| behavior (GO:0007610) | 4.85E-07 |
| blood vessel morphogenesis (GO:0048514) | 4.01E-05 |
| positive regulation of cell projection organization (GO:0031346) | 2.55E-04 |
| neuron development (GO:0048666) | 8.94E-09 |
| regulation of plasma membrane bounded cell projection organization (GO:0120035) | 8.16E-07 |
| anatomical structure morphogenesis (GO:0009653) | 2.66E-21 |
| blood vessel development (GO:0001568) | 1.11E-05 |
| regulation of anatomical structure size (GO:0090066) | 2.27E-05 |
| vasculature development (GO:0001944) | 9.35E-06 |
| regulation of actin filament-based process (GO:0032970) | 2.39E-04 |
| generation of neurons (GO:0048699) | 1.56E-10 |
| circulatory system development (GO:0072359) | 1.78E-08 |
| positive regulation of MAPK cascade (GO:0043410) | 7.96E-05 |
| regulation of inflammatory response (GO:0050727) | 2.85E-04 |
| regulation of membrane potential (GO:0042391) | 8.22E-05 |
| regulation of cell-cell adhesion (GO:0022407) | 7.06E-05 |
| neuron differentiation (GO:0030182) | 9.90E-10 |
| negative regulation of cell differentiation (GO:0045596) | 3.87E-06 |
| angiogenesis (GO:0001525) | 8.51E-04 |
| cell population proliferation (GO:0008283) | 1.24E-06 |
| neurogenesis (GO:0022008) | 6.06E-11 |
| positive regulation of defense response (GO:0031349) | 5.07E-04 |
| regulation of secretion (GO:0051046) | 3.81E-05 |
| embryonic morphogenesis (GO:0048598) | 5.68E-05 |
| positive regulation of cell adhesion (GO:0045785) | 2.26E-04 |
| anatomical structure formation involved in morphogenesis (GO:0048646) | 2.45E-07 |
| trans-synaptic signaling (GO:0099537) | 6.02E-04 |
| regulation of anatomical structure morphogenesis (GO:0022603) | 1.85E-06 |
| epithelial cell differentiation (GO:0030855) | 3.52E-05 |
| regulation of hormone levels (GO:0010817) | 1.70E-04 |
| metal ion transport (GO:0030001) | 4.92E-05 |
| enzyme-linked receptor protein signaling pathway (GO:0007167) | 8.90E-05 |
| epithelium development (GO:0060429) | 1.45E-07 |
| positive regulation of response to external stimulus (GO:0032103) | 2.08E-04 |
| positive regulation of developmental process (GO:0051094) | 7.17E-09 |
| negative regulation of developmental process (GO:0051093) | 2.83E-06 |
| regulation of cell adhesion (GO:0030155) | 1.72E-05 |
| regulation of MAPK cascade (GO:0043408) | 1.13E-04 |
| cell-cell signaling (GO:0007267) | 1.07E-05 |
| muscle structure development (GO:0061061) | 6.67E-04 |
| intracellular signaling cassette (GO:0141124) | 1.85E-05 |
| skeletal system development (GO:0001501) | 9.47E-04 |
| inorganic ion transmembrane transport (GO:0098660) | 7.60E-05 |
| secretion (GO:0046903) | 8.87E-04 |
| positive regulation of cell differentiation (GO:0045597) | 2.76E-05 |
| regulation of multicellular organismal development (GO:2000026) | 1.11E-07 |
| monoatomic cation transmembrane transport (GO:0098655) | 3.91E-04 |
| system development (GO:0048731) | 9.44E-20 |
| monoatomic ion transmembrane transport (GO:0034220) | 7.05E-05 |
| cell migration (GO:0016477) | 2.73E-05 |
| regulation of cell differentiation (GO:0045595) | 4.12E-08 |
| inorganic cation transmembrane transport (GO:0098662) | 5.46E-04 |
| actin filament-based process (GO:0030029) | 7.29E-04 |
| monoatomic cation transport (GO:0006812) | 1.43E-04 |
| nervous system development (GO:0007399) | 6.91E-11 |
| monoatomic ion transport (GO:0006811) | 3.27E-05 |
| regulation of developmental process (GO:0050793) | 6.00E-11 |
| regulation of cell development (GO:0060284) | 2.95E-04 |
| positive regulation of cell population proliferation (GO:0008284) | 9.73E-05 |
| response to organic cyclic compound (GO:0014070) | 3.46E-04 |
| multicellular organism development (GO:0007275) | 3.69E-18 |
| tissue development (GO:0009888) | 1.09E-07 |
| regulation of biological quality (GO:0065008) | 5.81E-12 |
| plasma membrane bounded cell projection organization (GO:0120036) | 3.48E-05 |
| cell projection organization (GO:0030030) | 2.30E-05 |
| positive regulation of cellular component organization (GO:0051130) | 6.33E-05 |
| cell development (GO:0048468) | 8.39E-09 |
| regulation of cellular component biogenesis (GO:0044087) | 2.84E-04 |
| response to endogenous stimulus (GO:0009719) | 1.51E-05 |
| regulation of cell population proliferation (GO:0042127) | 4.04E-06 |
| central nervous system development (GO:0007417) | 4.87E-04 |
| cell differentiation (GO:0030154) | 4.70E-13 |
| cellular developmental process (GO:0048869) | 4.90E-13 |
| positive regulation of multicellular organismal process (GO:0051240) | 7.38E-06 |
| positive regulation of cell communication (GO:0010647) | 4.67E-06 |
| cellular response to endogenous stimulus (GO:0071495) | 4.51E-04 |
| regulation of response to external stimulus (GO:0032101) | 5.16E-04 |
| cell motility (GO:0048870) | 6.02E-04 |
| positive regulation of signaling (GO:0023056) | 9.74E-06 |
| system process (GO:0003008) | 1.33E-06 |
| positive regulation of intracellular signal transduction (GO:1902533) | 6.17E-04 |
| regulation of locomotion (GO:0040012) | 9.57E-04 |
| embryo development (GO:0009790) | 9.67E-04 |
| intracellular signal transduction (GO:0035556) | 2.26E-05 |
| regulation of multicellular organismal process (GO:0051239) | 9.75E-09 |
| animal organ development (GO:0048513) | 1.97E-08 |
| anatomical structure development (GO:0048856) | 3.49E-15 |
| cellular response to chemical stimulus (GO:0070887) | 2.25E-05 |
| regulation of transport (GO:0051049) | 1.45E-04 |
| multicellular organismal process (GO:0032501) | 5.19E-18 |
| positive regulation of signal transduction (GO:0009967) | 3.54E-04 |
| developmental process (GO:0032502) | 1.54E-14 |
| positive regulation of response to stimulus (GO:0048584) | 5.98E-05 |
| cell communication (GO:0007154) | 2.99E-11 |
| cell surface receptor signaling pathway (GO:0007166) | 3.38E-04 |
| regulation of cellular component organization (GO:0051128) | 1.34E-04 |
| signaling (GO:0023052) | 1.06E-09 |
| regulation of cell communication (GO:0010646) | 2.49E-06 |
| regulation of localization (GO:0032879) | 8.26E-04 |
| signal transduction (GO:0007165) | 1.48E-08 |
| regulation of signaling (GO:0023051) | 1.02E-05 |
| response to chemical (GO:0042221) | 3.62E-05 |
| regulation of signal transduction (GO:0009966) | 6.61E-04 |
| positive regulation of cellular process (GO:0048522) | 1.79E-06 |
| regulation of response to stimulus (GO:0048583) | 2.13E-04 |
| cellular response to stimulus (GO:0051716) | 7.68E-07 |
| positive regulation of biological process (GO:0048518) | 2.22E-06 |
| negative regulation of biological process (GO:0048519) | 5.40E-05 |
| response to stimulus (GO:0050896) | 9.51E-08 |
| regulation of cellular process (GO:0050794) | 1.35E-06 |
| regulation of biological process (GO:0050789) | 1.45E-06 |
| biological regulation (GO:0065007) | 6.09E-07 |
| cellular process (GO:0009987) | 8.06E-04 |
| biological_process (GO:0008150) | 5.55E-05 |
| macromolecule biosynthetic process (GO:0009059) | 7.45E-04 |
| nucleobase-containing compound metabolic process (GO:0006139) | 9.90E-05 |
| Unclassified (UNCLASSIFIED) | 5.55E-05 |
| gene expression (GO:0010467) | 7.28E-05 |
| nucleic acid biosynthetic process (GO:0141187) | 6.08E-04 |
| RNA biosynthetic process (GO:0032774) | 4.24E-04 |
| RNA metabolic process (GO:0016070) | 1.08E-04 |
| nucleic acid metabolic process (GO:0090304) | 1.39E-06 |
| macromolecule catabolic process (GO:0009057) | 6.09E-04 |
| RNA processing (GO:0006396) | 4.58E-04 |
| mRNA metabolic process (GO:0016071) | 6.71E-04 |
| sensory perception of chemical stimulus (GO:0007606) | 1.04E-04 |
| sensory perception of smell (GO:0007608) | 9.53E-05 |
| detection of chemical stimulus involved in sensory perception of smell (GO:0050911) | 1.32E-05 |
| detection of chemical stimulus involved in sensory perception (GO:0050907) | 4.14E-06 |
| ribosome biogenesis (GO:0042254) | 4.69E-04 |
| ribonucleoprotein complex biogenesis (GO:0022613) | 8.87E-06 |

**Supplementary Table 8. Significant Biological Process Term associated with transcripts found to be upregulated between Saos-2-ADAR2 and Saos2-Empty cells.**

| **GO biological process complete** | **P value** |
| --- | --- |
| antigen processing and presentation of endogenous peptide antigen via MHC class II (GO:0002491) | 4.00E-04 |
| generation of ovulation cycle rhythm (GO:0060112) | 4.00E-04 |
| positive regulation of macrophage inflammatory protein 1 alpha production (GO:0071642) | 4.00E-04 |
| paranodal junction assembly (GO:0030913) | 1.52E-04 |
| prostate epithelial cord arborization involved in prostate glandular acinus morphogenesis (GO:0060527) | 1.52E-04 |
| prostate glandular acinus morphogenesis (GO:0060526) | 1.52E-04 |
| prostate glandular acinus development (GO:0060525) | 3.02E-05 |
| collagen-activated tyrosine kinase receptor signaling pathway (GO:0038063) | 4.67E-05 |
| collagen-activated signaling pathway (GO:0038065) | 7.99E-06 |
| detection of calcium ion (GO:0005513) | 9.81E-05 |
| detection of mechanical stimulus involved in sensory perception of pain (GO:0050966) | 1.81E-04 |
| sarcoplasmic reticulum calcium ion transport (GO:0070296) | 1.81E-04 |
| ventricular cardiac muscle cell action potential (GO:0086005) | 4.90E-05 |
| neuron projection arborization (GO:0140058) | 3.88E-04 |
| prostate gland epithelium morphogenesis (GO:0060740) | 7.84E-05 |
| detection of stimulus involved in sensory perception of pain (GO:0062149) | 1.61E-05 |
| prostate gland morphogenesis (GO:0060512) | 1.20E-04 |
| detection of mechanical stimulus involved in sensory perception (GO:0050974) | 6.19E-06 |
| synaptic membrane adhesion (GO:0099560) | 1.46E-04 |
| cardiac muscle cell action potential involved in contraction (GO:0086002) | 2.72E-06 |
| regulation of presynaptic membrane potential (GO:0099505) | 2.51E-04 |
| transmission of nerve impulse (GO:0019226) | 2.86E-07 |
| cardiac muscle cell contraction (GO:0086003) | 7.23E-06 |
| ovulation cycle process (GO:0022602) | 3.86E-05 |
| cardiac muscle cell action potential (GO:0086001) | 1.48E-05 |
| regulation of bone resorption (GO:0045124) | 2.02E-04 |
| regulation of bone remodeling (GO:0046850) | 6.63E-05 |
| neuromuscular junction development (GO:0007528) | 2.30E-04 |
| cardiac muscle contraction (GO:0060048) | 8.03E-07 |
| prostate gland development (GO:0030850) | 2.95E-04 |
| calcium-dependent cell-cell adhesion via plasma membrane cell adhesion molecules (GO:0016339) | 2.95E-04 |
| detection of mechanical stimulus (GO:0050982) | 9.64E-05 |
| synapse assembly (GO:0007416) | 1.60E-08 |
| heart contraction (GO:0060047) | 6.79E-07 |
| actin-mediated cell contraction (GO:0070252) | 2.06E-05 |
| heart process (GO:0003015) | 4.31E-07 |
| ovulation cycle (GO:0042698) | 7.69E-05 |
| gland morphogenesis (GO:0022612) | 1.29E-06 |
| action potential (GO:0001508) | 2.17E-07 |
| urogenital system development (GO:0001655) | 2.58E-04 |
| regulation of heart rate (GO:0002027) | 5.20E-06 |
| striated muscle contraction (GO:0006941) | 8.66E-07 |
| regulation of tissue remodeling (GO:0034103) | 1.15E-04 |
| sensory perception of pain (GO:0019233) | 3.14E-04 |
| cardiac conduction (GO:0061337) | 3.79E-04 |
| outflow tract morphogenesis (GO:0003151) | 1.52E-04 |
| odontogenesis of dentin-containing tooth (GO:0042475) | 2.79E-04 |
| actin filament-based movement (GO:0030048) | 1.95E-04 |
| cell junction assembly (GO:0034329) | 7.28E-10 |
| cell-cell adhesion via plasma-membrane adhesion molecules (GO:0098742) | 3.74E-09 |
| cardiac chamber morphogenesis (GO:0003206) | 4.69E-05 |
| synapse organization (GO:0050808) | 4.87E-11 |
| homophilic cell adhesion via plasma membrane adhesion molecules (GO:0007156) | 7.12E-06 |
| cardiac chamber development (GO:0003205) | 7.63E-06 |
| calcium-mediated signaling (GO:0019722) | 2.11E-05 |
| cell-cell adhesion (GO:0098609) | 4.82E-15 |
| extracellular matrix organization (GO:0030198) | 4.12E-08 |
| extracellular structure organization (GO:0043062) | 4.40E-08 |
| external encapsulating structure organization (GO:0045229) | 4.69E-08 |
| response to calcium ion (GO:0051592) | 3.11E-04 |
| regulation of blood circulation (GO:1903522) | 7.18E-07 |
| regulation of heart contraction (GO:0008016) | 1.09E-05 |
| regulation of fat cell differentiation (GO:0045598) | 3.51E-04 |
| cell junction organization (GO:0034330) | 7.59E-13 |
| regulation of muscle contraction (GO:0006937) | 1.13E-04 |
| calcium ion transmembrane transport (GO:0070588) | 2.59E-05 |
| heart morphogenesis (GO:0003007) | 1.92E-05 |
| muscle system process (GO:0003012) | 6.48E-06 |
| potassium ion transport (GO:0006813) | 3.49E-04 |
| muscle contraction (GO:0006936) | 8.84E-05 |
| cell adhesion (GO:0007155) | 1.32E-14 |
| calcium ion transport (GO:0006816) | 8.32E-05 |
| blood circulation (GO:0008015) | 9.58E-07 |
| regulation of system process (GO:0044057) | 1.80E-08 |
| ossification (GO:0001503) | 7.33E-05 |
| adenylate cyclase-modulating G protein-coupled receptor signaling pathway (GO:0007188) | 3.80E-04 |
| regulation of membrane potential (GO:0042391) | 6.07E-06 |
| circulatory system process (GO:0003013) | 3.54E-06 |
| animal organ morphogenesis (GO:0009887) | 1.14E-09 |
| behavior (GO:0007610) | 1.12E-05 |
| metal ion transport (GO:0030001) | 8.96E-06 |
| inorganic ion transmembrane transport (GO:0098660) | 2.33E-06 |
| monoatomic ion transmembrane transport (GO:0034220) | 1.35E-06 |
| heart development (GO:0007507) | 8.69E-05 |
| monoatomic cation transmembrane transport (GO:0098655) | 2.48E-05 |
| tissue morphogenesis (GO:0048729) | 9.33E-05 |
| inorganic cation transmembrane transport (GO:0098662) | 1.25E-04 |
| regulation of secretion (GO:0051046) | 2.68E-04 |
| tube morphogenesis (GO:0035239) | 1.37E-04 |
| generation of neurons (GO:0048699) | 7.50E-07 |
| monoatomic cation transport (GO:0006812) | 6.99E-05 |
| monoatomic ion transport (GO:0006811) | 9.91E-06 |
| anatomical structure morphogenesis (GO:0009653) | 9.66E-12 |
| circulatory system development (GO:0072359) | 3.14E-05 |
| neuron differentiation (GO:0030182) | 9.23E-06 |
| neurogenesis (GO:0022008) | 8.10E-07 |
| neuron development (GO:0048666) | 1.55E-04 |
| central nervous system development (GO:0007417) | 1.70E-04 |
| regulation of multicellular organismal development (GO:2000026) | 1.45E-05 |
| nervous system development (GO:0007399) | 4.43E-08 |
| system development (GO:0048731) | 4.41E-13 |
| multicellular organism development (GO:0007275) | 5.72E-14 |
| regulation of biological quality (GO:0065008) | 2.95E-09 |
| regulation of multicellular organismal process (GO:0051239) | 5.32E-09 |
| positive regulation of multicellular organismal process (GO:0051240) | 6.84E-05 |
| regulation of developmental process (GO:0050793) | 1.26E-06 |
| tissue development (GO:0009888) | 7.38E-05 |
| regulation of transport (GO:0051049) | 3.46E-04 |
| anatomical structure development (GO:0048856) | 8.53E-12 |
| cell differentiation (GO:0030154) | 1.04E-07 |
| cellular developmental process (GO:0048869) | 1.42E-07 |
| animal organ development (GO:0048513) | 6.87E-06 |
| multicellular organismal process (GO:0032501) | 6.03E-14 |
| system process (GO:0003008) | 2.44E-04 |
| cell development (GO:0048468) | 2.13E-04 |
| developmental process (GO:0032502) | 1.10E-10 |
| cell communication (GO:0007154) | 1.26E-06 |
| signaling (GO:0023052) | 1.87E-05 |
| signal transduction (GO:0007165) | 2.53E-04 |
| biological regulation (GO:0065007) | 4.84E-06 |
| regulation of biological process (GO:0050789) | 6.87E-05 |
| biosynthetic process (GO:0009058) | 2.55E-04 |
| macromolecule biosynthetic process (GO:0009059) | 2.30E-06 |
| gene expression (GO:0010467) | 1.16E-05 |
| nucleobase-containing compound biosynthetic process (GO:0034654) | 3.18E-04 |
| nucleic acid metabolic process (GO:0090304) | 1.29E-05 |
| RNA metabolic process (GO:0016070) | 5.34E-05 |
| nucleic acid biosynthetic process (GO:0141187) | 3.87E-05 |
| RNA biosynthetic process (GO:0032774) | 5.24E-05 |
| detection of chemical stimulus involved in sensory perception of smell (GO:0050911) | 2.10E-04 |
| ribonucleoprotein complex biogenesis (GO:0022613) | 2.26E-04 |
| sensory perception of smell (GO:0007608) | 1.45E-04 |

**Supplementary Table 9. List of primers used for Real-Time RT-PCR**

| **Primers** | **Forward Sequence** | **Reverse Sequence** |
| --- | --- | --- |
| **GAPDH** | 5’-GACAAGCTTCCCGTTCTCAG-3’ | 5’-ACAGTCAGCCGCATCTTCTT-3’ |
| **ADAR2** | 5’-CAATGCGAGCATCCAAACGTGG-3’ | 5’-ATGACCTCAATAGCGGAGTCGC-3’ |
| **RUNX2** | 5’-TTACTTACACCCCGCCAGTC-3’ | 5’-TATGGAGTGCTGCTGGTCTG -3’ |
| **SP7** | 5’-TGCTTGAGGAGGAAGTTCAC-3’ | 5’-AGGTCACTGCCCACAGAGTA-3’ |
| **ALP** | 5’-GGACATGCAGTACGAGCTGA-3’ | 5’-CCACCAAATGTGAAGACGTG-3’ |
| **COL1A2** | 5’-AAAACATCCCAGCCAAGAACTG-3’ | 5’- AAACTGGCTGCCAGCATTG-3’ |
| **PRKCA** | 5’-CGACTGTCTGTAGAAATCTGG-3’ | 5’-CACCATGGTGCACTCCACGTC-3’ |
| **DMP1** | 5’-CAGGAGCACAGGAAAAGGAG-3’ | 5’-CTGGTGGTATCTTGGGCACT-3’ |
| **MEPE** | 5’-CAAAAGCACCCATCGTATTC-3’ | 5’-ATATCGTTGCTGCCCTCTAC-3’ |
| **NANOG** | 5’-TGCCTCACACGGAGACTG-3’ | 5’-GCTATTCTTCGGCCAGTT-3’ |
| **COL4A1** | 5’-CTGCCTGGAGGAGTTTAGAAG-3’ | 5’-GAACATCTCGCTCCTCTCTATG-3’ |
| **SERPINH1** | 5’-AGCTTCGCTGATGACTTCGT-3’ | 5’-TCTCATCCCAGTGTGGCTTG-3’ |
| **TENM1** | 5’-GCATAGTTCCTGTTTGTCCA-3’ | 5’-TCTGCACATCTTGAGTAGAC-3’ |
| **SWAP70** | 5’-TCCCAGCTCAAGGTCCTTTC-3’ | 5’-ACACAGAGGGTCCAACACAT-3’ |

**Supplementary Table 10. List of primers used for sequencing**

| **Primers** | **Forward Sequence** | **Reverse Sequence** |
| --- | --- | --- |
| **COPA** | 5’-TTCACATTGCTTGGGCACTT-3’ | 5’-CGCCAGATCTTCACTTGACG-3’ |
| **COG3** | 5’-AGCATTGTCTGCCTGCATTC-3’ | 5’-CAATGTCACAGGCAGCTTTG-3’ |
| **IGFBP7** | 5’-TCTCCTCTTCCTCCTCTTCGGAC-3’ | 5’-GGTAGCGGCTCTTGCACACG-3’ |
