# Supplementary material for: ADAR2 induces the differentiation of osteosarcoma cells by editing activity on IGFBP7: new implications for therapy
Source: Bone Res. 2026 Apr 3;14:38. doi: 10.1038/s41413-026-00516-6 (PMC13046735; doi:10.1038/s41413-026-00516-6)
Supplement: Supplementary file 8 — Supplementary figure Legend [file 41413_2026_516_MOESM8_ESM.docx]

**Supplementary Figure 1. Correlation between expression levels of ADAR2 and genes associated with genetic signature in Osteosarcoma. a,d)** Correlation plots between *ADAR2* and **a)** *IGF2*, **b)** *VCAM1*, **c)** *ZFP90* and **d)** *FBXL5* in 127 osteosarcoma patients (R2-http://hgserver1.amc.nl, GSE42352).

**Supplementary Figure 2. Overexpression of ADAR2 in OS cell lines. a-b)** Real Time RT-PCR expression analysis of *ADAR2* in **a)** Saos-2 and **b)** 143B cells transfected with ADAR2-pEGFP-C3 (pADAR2), ADAR2 E/A-pEGFP-C3 (pADAR2 E/A) or Empty-pEGFP-C3 (pEmpty) vectors. **c-d)** Western blot analysis of Adar2 protein expression in transfected cells. *Upper panels*: representative blots; *lower panels*: densitometric analysis. Results are expressed as mean±sd and are reported as individual data points of independent experiments. *p<0.05; **p<0.01; ***p<0.001; ****p<0.0001 *vs* pEmpty transfected cells. **e-f)** Confocal microscopy analysis showing the nucleolar localization of ADAR2-EGFP protein in **e)** Saos-2 and **f)** 143B transfected with pADAR2, pADAR2 E/A or pEmpty vectors.

**Supplementary Figure 3. PXD101 and FK228 treatment of Saos-2 cells. a-b)** Cell viability analysis of Saos-2 overexpressing pADAR2, pADAR2 E/A and pEmpty vectors treated with increasing concentrations of PXD101 (0.25, 0.5, 1, 5, 10, 20 and 30 μM, *left panel*) for 2 days and of FK228 (0, 2.5, 5, 10, 20 and 30 nM, *right panel*) for 3 days. The concentration of drugs able to reduce by 50% (GI50) cell viability is reported in the upper part of each graph. Results are expressed as mean±sd of at least three independent experiments. *p<0.05; **p<0.01; ***p<0.001 *vs* pEmpty transfected cells.

**Supplementary Figure 4. *In vivo* experiments with 143B cell lines.** Seven-weeks-old NSG male mice were intratibially injected with 143B cells transfected with pADAR2, pADAR2 E/A or pEmpty vectors; after 3 weeks animals were sacrificed. **a)** Representative X-Rays pictures of primary bone tumors. **b)** Quantification of the tumor volume. Results are expressed as mean±sd. **c)** Number of animals with metastases in liver, lungs and kidneys at sacrifice. **d)** Hematoxylin/Eosin staining of liver (*upper panels*), lung (*middle panels*) and kidney metastases (*lower panels*). Smaller nodules were indicated by black arrowheads. A representative picture for each organ for all the animals is reported. Original magnification: 5X.

**Supplementary Figure 5. Ki67 staining of metastases from animals intratibially injected with 143B cells.** Representative pictures of ki67 immunohistochemistry of liver (*upper panels*), lung (*middle panels*) and kidney metastases (*lower panels*). A representative picture for each organ for all the animals is reported. Original magnification: 40X.

**Supplementary Figure 6. IGFBP7 Editing analysis in transfected 143B cells.** *Lower panels*: percentage of editing in *IGFBP7* transcript of 143B cells overexpressing pADAR2, pADAR2 E/A and pEmpty vectors. *Upper panels*: sequence chromatograms of the endogenous transcript and editing levels of IGFBP7. Arrows indicate editing positions. Results are expressed as mean±sd and are reported as individual data points of independent experiments. *p<0.05 *vs* pEmpty transfected cells. ^##^p<0.01 *vs* pADAR2 transfected cells.
